# Bioprospecting and Structure of Fungal Endophyte Communities Found in the Brazilian Biomes, Pantanal, and Cerrado

**DOI:** 10.3389/fmicb.2018.01526

**Published:** 2018-07-24

**Authors:** Sandriele A. Noriler, Daiani C. Savi, Rodrigo Aluizio, Angela M. Palácio-Cortes, Yvelise M. Possiede, Chirlei Glienke

**Affiliations:** ^1^Department of Basic Pathology, Federal University of Paraná, Curitiba, Brazil; ^2^Department of Genetics, Federal University of Paraná, Curitiba, Brazil; ^3^Department of Zoology, Federal University of Paraná, Curitiba, Brazil; ^4^Department of Biology, Federal University of Mato Grosso do Sul, Campo Grande, Brazil

**Keywords:** *Vochysia divergens*, *Stryphnodendron adstringens*, natural antimicrobials, medicinal plants, *Diaporthe*

## Abstract

Medicinal plants have been recognized as hosts of high diverse endophytic microorganisms, including fungi that produce secondary metabolites with biological activity. Two biomes in Brazil, Pantanal (wetland), and Cerrado (savannah), are known as biodiversity hotspots, and despite their importance as a reservoir for several species, knowledge about the fungal biodiversity in these biomes is very limited. Fungal endophytic communities associated with leaves and petioles of the medicinal plants *Vochysia divergens* (from Pantanal) and *Stryphnodendron adstringens* (from Cerrado) were analyzed and studied for their antimicrobial activity against human and plant pathogens. A total of 1,146 isolates of endophytic fungi were obtained from plants collected in January and June of 2016 and grouped into 124 morphotypes. One isolate of each morphotype was identified by sequencing of internal transcribed spacer (ITS) region of the rDNA gene, which revealed the presence of 24 genera, including 3 possible new genera, and 48 taxa. Differences in the endophytic community according to the biomes were observed concerning the analyzed morphotypes. However, when we analyzed the diversity of genera and richness, they were similar for both plants, with *Diaporthe, Phyllosticta*, and *Neofusicoccum* as dominant genera. In addition, the community composition of *V. divergens* differs according to the analyzed plant tissues (petiole and leaf). These data suggested that both, the plant species and plant tissues play a role in the composition of endophytic community. As regards the biotechnological potential, 5 isolates showed activity against the phytopathogens *Phyllosticta citricarpa, Colletotrichum abscissum*, and *Fusarium verticilioides*, and 8 isolates showed high activity against clinical pathogens and were selected for the production of crude extract in different culture media. Extract from cultivation of *Diaporthe* sp. LGMF1548 and LGMF1583 and *Neofusicoccum brasiliense* LGMF1535 showed activity against methicillin-resistant *Staphylococcus aureus, Klebssiella pneumonia*, and *Candida albicans*. In addition, extracts of *Diaporthe* cf. *heveae* LGMF1631 inhibited 90% of the mycelial growth of the *P. citricarpa* and 70% of *C. abscissum* and may represent an alternative to be used in the biological control of these phytopathogens. Future research will focus on the chemical characterization and structural elucidation of these bioactive compounds.

## Introduction

In the last years, medicinal plants have gained great importance as a reservoir of new endophytic strains with biotechnological potential (Gomes et al., 2013; Köberl et al., 2013; Savi et al., 2018). The endophyte-host interaction can interfere with the plant growth, development, and defense against pathogens and insects (Murali et al., 2007; Santoyo et al., 2016). However, the endophytic community is highly variable and depends on several components, such as host species and environmental conditions (Dudeja and Giri, 2014). In this context, two Brazilian biomes are particularly prominent due to the biodiversity of the flora: the savannah (Cerrado) and the wetland (Pantanal).

The Pantanal is a wetland located in the center of South America, in the Upper Paraguay River Basin, characterized by the change between periods of flood and drought, and the flora diversity found in this biome may be the result of seasonality in this area (Junk et al., 1989). Floods can inundate 80% of the entire Pantanal, and by contrast, during the dry season, most flooded areas will be dry. The biodiversity of Pantanal constitutes a valuable natural resource, and it is recognized as one of the most important freshwater ecosystems in the world. However, the unsustainable land use in this biome has been harming this peculiar biodiversity (Alho and Silva, 2012), and effective alternatives to conservation are necessary, such as bioprospecting studies to associate an economic value to the diversity present in this area.

The Brazilian savannah (Cerrado) is a neighboring biome of Pantanal that biogeographically influences its biodiversity and hosts a huge diversity of flora (Silva and Bates, 2002). The Cerrado is classified as one of the most diverse places in the world (Myers et al., 2000), with an estimated composition of more than 6,000 vascular plant species (Oliveira-Filho and Ratter, 2002; Felfili and Fagg, 2007). The diversity composition is result of the adverse environmental conditions caused by the fire, which selects the vegetation, changing the dominant plant species (Felfili and Fagg, 2007).

As these biomes are located in unexplored transition areas and are subject to adverse climatic changes, they were declared as priority conservation areas by the Brazilian government. Studies in the Pantanal and Cerrado are usually on animal and plant biodiversity (Hyde and Soytong, 2008; Porras-Alfaro and Bayman, 2011). A very limited number of studies in these biomes are conducted on endophytic microorganisms, and most of them are focused on aspects of bioprospecting without community analysis (Carvalho et al., 2012; Glienke et al., 2012; Vieira et al., 2014; Savi et al., 2016; Gos et al., 2017; Hokama et al., 2017; Parpinelli et al., 2017; Sousa et al., 2017).

The environmental conditions and the composition of the plants present in the Pantanal and Cerrado represent an unrivaled variety of habitats, which can have a significant influence on the endophytic community. As there are no studies comparing the species richness and the endophytic community between these two biomes, some questions remain unclear: What is the taxonomic composition and diversity of these endophytic fungi? Is the endophytic community biome-specific? Is the diversity structured or spread among these neighboring biomes? What is the biotechnological potential of these endophytic fungi?

To address these questions, we analyzed and bioprospected the fungal endophytic community from two medicinal plants: *Vochysia divergens* Pohl (Vochysiaceae) and *Stryphnodendron adstringens* (Mart.) Coville (Fabaceae), found in Pantanal and Cerrado biomes, respectively. *Vochysia divergens* is considered as monodominant remaining during dry and flood season in the Pantanal (Junk et al., 2006) and *S. adstringens* is a native Cerrado species widely used in traditional medicine for its biological properties. These plants were selected according to the biome specificity, the medicinal properties and the lack of endophytic community studies.

## Materials and methods

### Plant material

**Figure 1:**
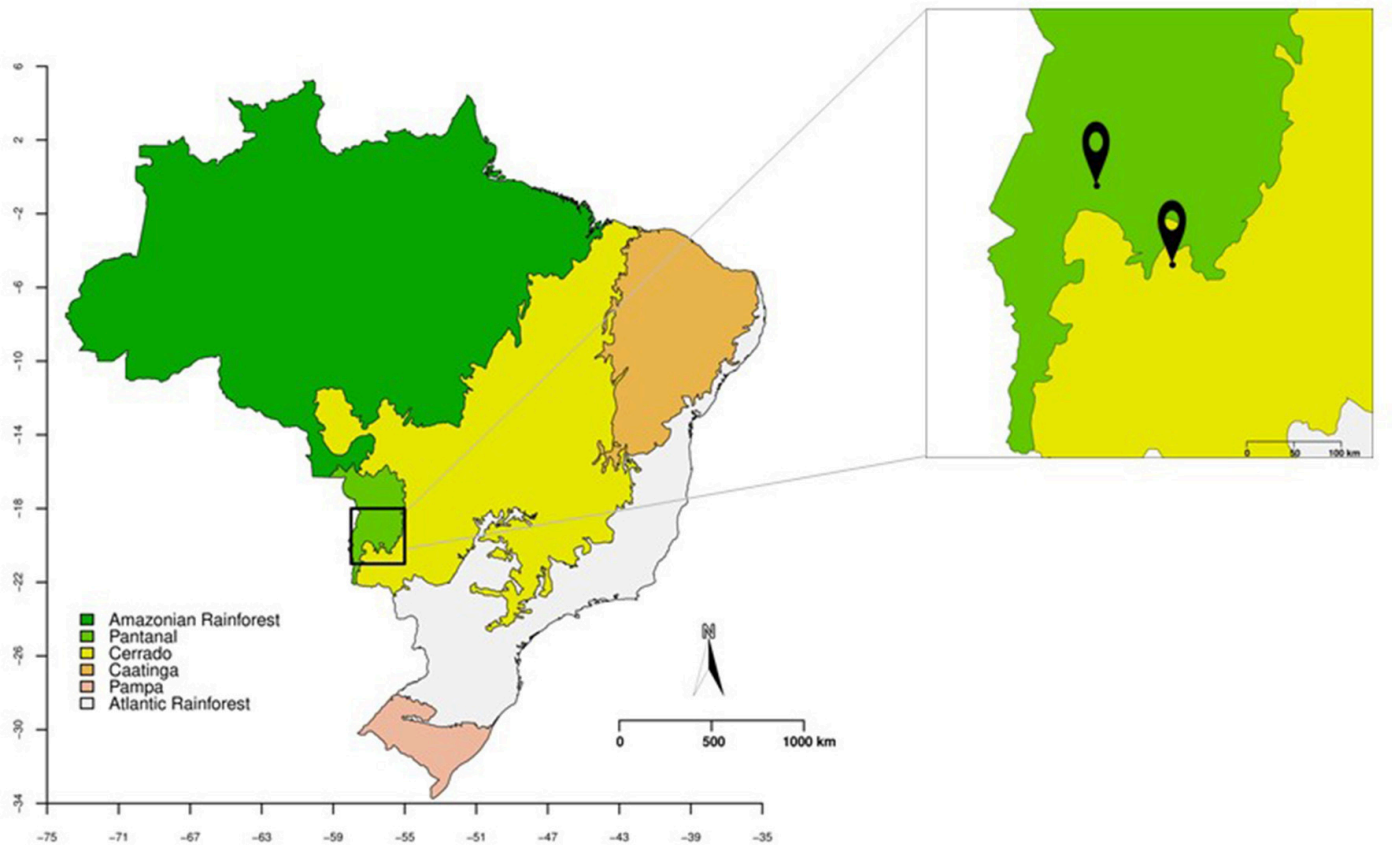
Brazil map showing the Pantanal (in green) and Cerrado (in yellow) biomes. In the magnified box are represented the collecting points of leaves and petioles of *Vochysia divergens* (Pantanal) and *Stryphnodendron adstringens* (Cerrado).

The leaves and petioles of *S. adstringens* and *V. divergens* were collected in January and June 2016 from two Brazilian biomes, Cerrado (Savanna) and Pantanal. The collection of leaves and petioles of *V. divergens* was carried out in Pantanal of Miranda River (19°32′36.9″S 57°02′21.8″W), along of MS184 Road in Corumbá, Mato Grosso do Sul State, Brazil (Figure 1; were performed using R 3.4.4 R Core Team, 2017, source IBGE, 2004). The *S. adstringens* leaves and petioles were collected in Cerrado along the BR262 road (20°18′10.8″S 56°15′44.3″W) in Miranda, Mato Grosso do Sul State, Brazil (Figure 1). The samples of the *S. adstringens* were collected from 10 plants in January and 23 in June, and the samples of *V. divergens* were collected from 8 plants in January and 24 in June. For both species, 10 leaves and 10 petioles were collected for each individual plant. The plant tissues were stored at 4°C and isolation was performed within 72 h.

The January collection was used as a preliminary test for the epiphytic elimination protocol. The isolated endophytes of this collection were used only for bioprospecting and phylogenetic studies and were not included in the community analysis. With the optimized epiphytic elimination protocol, a second collection was performed in June, and the isolates obtained were used for endophytic community evaluation, phylogeny, and bioprospecting studies.

### Isolation and morphotyping

The leaves and petioles, without lesions, from healthy plants, were used for endophytic fungi isolation using the 6 steps protocol described by Petrini (1986), with modifications: the leaves and petioles were submerged in autoclaved water for 1 min, immersion in 70% ethanol (v/v) for 1, 3 min in sodium hypochlorite 3% (v/v), 30 s in 70% ethanol (v/v), and then washed in sterilized distilled water for 1 min. After surface sterilization, the samples were fragmented into 5 pieces of 8 x 8 mm and aseptically transferred to plates containing potato dextrose agar (PDA), pH 5.8. The plates were incubated at 28°C for 30 days, and the endophytic growth was checked daily, the emerging mycelia were transferred to a new plate and stored at 4°C on PDA for further identification.

The isolation frequency (IF) of endophytes was calculated as the number of fragments from which one or more endophytic fungi were isolated, divided by the total number of fragments analyzed (Petrini et al., 1982). The isolates were assigned to morphotypes based on growth rate, colony color, hypha aspects, presence/absence and morphology of spores. One isolate from each morphotype was randomly selected for molecular identification and bioprospecting. For each isolate, a pure culture was obtained through single spore culture approach, according to Gilchrist-Saavedra et al. (2006). The isolates were deposited in the Laboratório de Genética de Microrganismos (LabGeM) culture collection, at the Federal University of Paraná, Brazil (http://www.labgem.ufpr.br/).

### Isolates identification

Isolates were cultivated on PDA for 3 days at 28°C, and genomic DNA extraction was performed according to Raeder and Broda (1985). Primers V9G (de Hoog and Gerrits van den Ende, 1998) and ITS4 (White et al., 1990) were used to amplify the internal transcribed spacer region (ITS) of the nuclear ribosomal RNA gene. PCR reaction was performed using Top Taq Master Mix (QIAGEN), purified with the Exo1 and FastAP enzymes (ThermoFisher Scientific, USA) and sequenced using the BigDye Terminator Cycle Sequencing Kit v 3.1 kit (Applied Biosystems, Foster City, CA, USA). DNA sequences were obtained on an ABI Prism 3500 sequencer (Applied Biosystems, Foster City, CA, USA). Chromatograms were inspected using MEGA 7 software (Kumar et al., 2015) and BioEdit 7.0.5.3 (Hall, 1999) and compared to sequences available in the NCBI / GenBank database (National Center for Biotechnology Information -Http://www.ncbi.nlm.nih.gov/BLAST/) using the tool Blast, and comparing with the types strains found in MycoBank database (http://www.mycobank.org/).

The phylogenetic analyses were performed using sequences from the available type strains of each genus identified in addition to the sequences generated in this study. Alignments of DNA sequences were performed using the Mafft (Katoh and Toh, 2008; https://mafft.cbrc.jp/alignment/server/) and manually corrected when necessary in MEGA version 7 (Tamura et al., 2013). Phylogeny based on Bayesian inference was performed in MrBayes version 3.2.1 (Ronquist et al., 2011), using two parallel runs with one cold and three heated chains each, using the number of generations needed to reach split frequencies of ≤0.01 and a sampling frequency set to every 100 generations. The posterior probability values were calculated after discarding the first 25% of the generated trees as burn-in. Resulting trees were plotted in FigTree v.1.4.2 (http://tree.bio.ed.ac.uk/software/figtree/). The substitution model was selected for each genus using the MEGA software. All sequences obtained were deposited in GenBank, access codes listed in Table S1.

### Analysis of endophytic fungal community

Analyzes of the endophytic community were performed at genera and morphotypes levels, comparing fungal community in both plants, and between leaves and petioles tissues. To analyze the data a matrix of abundance was used, and plants with no endophyte recovered were removed from the analysis, as well as, those rare taxa that accomplished <5 percent of total abundance for genera and for morphotypes. The diversity index, total abundance, and richness were calculated based on the identified morphotypes and genera, without exclusions.

The community structure was assessed using Shannon-Weaver (H′) diversity index (Shannon and Weaver, 1949), Simpson (1-D) index (Simpson, 1949), and Pielou (J) evenness (Pielou, 1975). Absolute abundance (N) and richness (S) also were estimated. Data were compared using two-way ANOVA for medians (Wilcox, 2012) once the data is non-parametric and no transformation normalized it. We also performed a non-metric multidimensional scaling (nMDS) analysis followed by analysis of similarity (ANOSIM) (Clarke, 1993), Permutational multivariate analysis of variance (PERMANOVA) (Anderson, 2001) using Bray-Curtis distance matrix and Indicator Value (IndVal) analysis (Dufrene and Legendre, 1997) to better elucidate taxa distribution and structures amongst plants. The indicator species are defined as the most characteristic species of each group, found mainly in a single group and present in most of the samples belonging to such group.

All the analyses and graphics were performed using R 3.4.4 (R Core Team, 2017) and packages vegan (Oksanen et al., 2018), labdsv (Roberts, 2016), WRS2 (Mair et al., 2017) and ggplot2 (Wickham, 2009). The sampling effort and richness based on both taxonomic levels were evaluated using rarefaction curves (Sanders, 1968).

### Biological activities of endophytic fungi

#### Antifungal activity

The antifungal potential of the endophytic isolates was initially assessed by dual culture method (Badalyan et al., 2002) against the phytopathogens *Colletotrichum abscissum* (CA142), *Phyllosticta citricarpa* (LGMF06), and *Fusarium verticillioides* (LGMF1762). These pathogens were selected because of their importance in citrus (*C. abscissum* and *P. citricarpa*) and maize (*Fusarium verticillioides*) crops (Kotzé, 1981; Leslie and Summerell, 2006; Lima et al., 2011). The endophytic and pathogenic strains were previously cultured on PDA medium pH 5.8, for 7–21 days. One disc (6 mm) from the endophyte and one from phytopathogen were inoculated in opposites sides of the petri dish and incubated at 28°C for 10 days for *F. verticillioides*, 14 days for *C. absissum* and 21 days for *P. citricarpa*. As negative control, only the phytopathogen was inoculated. The experiments were performed in triplicate.

The percentage of inhibition was calculated according to Quiroga et al. (2001), comparing the growth diameter of phytopathogen on pairing plates to control plates. The antifungal activity was classified as low (50–59%), moderate (60–69%), and high (≥70%) according to the percentage of inhibition.

#### Antibacterial activity

The antibacterial activity against clinical pathogens was evaluated using overlap method according to Mapperson et al. (2014) against methicillin-sensitive *Staphylococcus aureus* (MSSA) (ATCC 27213), methicillin-resistant *S. aureus* (MRSA) (ATCC 33591), *Klebsiella pneumoniae* producer of the enzyme KPC (*K. pneumonia* carbapenemase) (BACHC-KPC), *Acinetobacter baumannii* (BACHAC-ABA), and *Pseudomonas aeruginosa* (ATCC 27853). All the pathogens were selected on the basis of their importance in human diseases and antibiotic resistance (CDC, 2013). The bacteria were cultivated for 12 h at 37°C, and diluted according to the McFarland standard 0.5 scales. One 6 mm disk of each endophyte was inoculated into the center of the PDA plate and incubated at 28°C until the endophyte reaches more than 2 cm in diameter. The bacteria were then streaked with a cotton swab on the boards of these plates and incubated at 36°C for 24 h. The experiments were performed in duplicate, and saline (0.85%) was used as negative control. The analysis was based on the spectrum of action of endophytes: the number of pathogens inhibited; and intensity of inhibition classified as no activity (–), low (+), moderate (++), and high (+++) activity. The endophytes with higher activity and a large spectrum of action were selected for extract production.

### Biological activities from extracts of endophytic fungi

#### Extracts production

The endophytes selected in the biological activity assays were used for the preparation of extracts after fermentation in two liquid media, Malt extract (ME) (Schulz et al., 1995) and Czapeck yeast extract (CY) (Wiseman, 1975). The isolates were previously cultivated for 7 days on PDA, pH 5.8 at 28°C. Four mycelial discs (6 mm) were then added to 250 mL (500 mL Erlenmeyer flasks) of ME medium (Schulz et al., 1995) or CY medium (Wiseman, 1975), and incubated under agitation for 10 days (180 rpm, 28°C). The cultures were filtered-off on Whatmann filter and the water fraction was extracted with EtOAc (3 × 100 mL). The combined organics were evaporated in *vacuo* at 45°C and diluted in methanol at 10 mg/mL.

#### Activity of extracts against phytopathogens

The extracts produced by the endophytes were evaluated against phytopathogens *P. citricarpa, C. abscissum*, and *F. verticillioides*. One hundred microliter of each extract were spread over the surface of PDA medium (48 × 12 mm plates), using the Drigalski spatula, and one mycelial disc of each phytopathogen was inoculated in the center of the plates. As the positive control, the fungicide Derosal® (1.0 mg/mL) was used for *P. citricarpa* and *F. verticillioides* and the fungicide Carbendazim (0.1 mg/mL) was used for *C. abscissum*. Methanol was used as the negative control. Plates were incubated in BOD at 28°C for 7 days for *F. verticillioides*, 10 days for *C. abscissum*, and 21 days for *P. citricarpa*. The growth inhibition was assessed comparing the diameter of the colony in the presence of treatment and controls (Savi, 2011). This experiment was performed in triplicate and the data were submitted to analysis of parametric variance (ANOVA) in GraphPad Prims v. 6.01.

#### Activity of extracts against the clinical pathogens

The antimicrobial activity of extracts produced by the endophytes was evaluated using the pathogens *S. aureus* (MSSA) (ATCC 27213) and methicillin-resistant *S. aureus* (MRSA) (ATCC 33591), *P. aeruginosa* (ATCC 27853), *A. baumannii* (BACHAC-ABA), *K. pneumoniae* (KPC) (BACHC-KPC), *Stenotrophomonas maltophilia* (BACHC-SMA), *Enterobacter cloacae* a producer of the enzyme VIM (Verona integron-encoded metallo-b-lactamase) (BACHC-VIM), and *Candida albicans* (ATCC 10231). The pathogens were inoculated previously on 7 mL of LB broth and incubated at 37°C, 180 rpm for 10 h. The culture was inoculated on TSB (Tryptone Soy Broth TM Medium) plate with a cotton swab and on these plates were placed discs impregnated with each 10 μL of extract. The plates were incubated at 37°C for 24 h. As controls, one disc with a standard antibiotic with activity against each of the bacteria and pure methanol were used (CLSI, 2015; Savi et al., 2015). The antibacterial activity was evaluated by the measure of the inhibitory zone.

## Results

### Isolation, morphotyping, and phylogenetic analyses of endophytic fungi from *V. divergens* and *S. adstringens*

A high number (1,146) of cultivable endophytic fungal was recovered from *V. divergens* and *S. adstringens* in collections performed in January and June 2016. Based on the morphological analysis the isolates were grouped into 124 morphotypes, 116 of them were isolated from both plants, seven were isolated exclusively from *V. divergens*, and one was isolated exclusively from *S. adstringens*. Regarding tissue specificity, five morphotypes were recovered exclusively from leaf tissues (morphotypes 29, 31, 111, and 114 were recovered exclusively from *V. divergens* and morphotype 106 was isolated exclusively from *S. adstringens*) and three morphotypes were recovered only from petiole (morphotypes 78 and 80 from *V. divergens* and morphotype 54 from *S. adstringens*) (Table S1).

**Figure 2:**
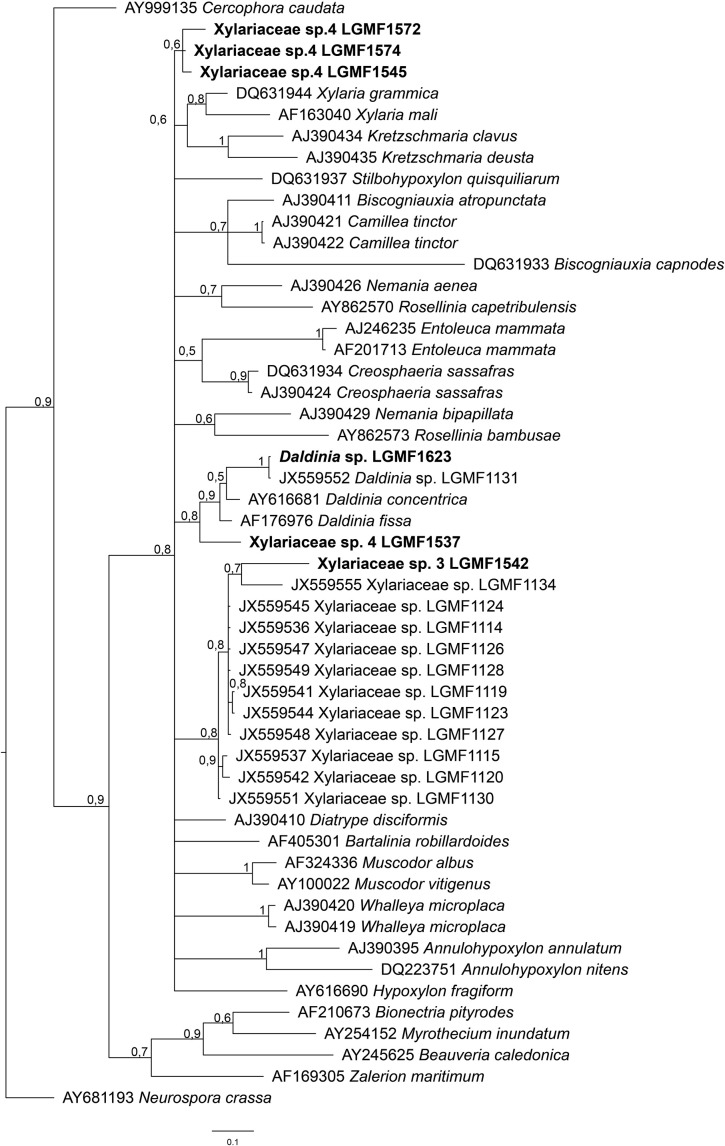
Bayesian phylogenetic tree based on ITS partial sequence of LGMF1572, LGMF1574, LGMF1545, LGMF1613, LGMF1537, and LGMF1542 (bold) sequence of genera of Xylariaceae family. The data matrix had 52 taxa and 416 characters. The tree was rooted to: *Neurospora crassa* (AY681193). Scale bar shows 0.1 changes and Bayesian posterior probability values are indicated at the nodes. T: type strain.

The morphotypes were identified at genus and species level based on Bayesian phylogeny analysis using sequence of ITS region (Supplementary material: Figures S2–S27; Figure 2), and grouped at phylum, class and family level following the classification present at Mycobank and CABI databases (myocbank.org and speciesfungorum.org). The majority of the isolates belong to Phylum Ascomycota (95.6% of isolates from *S. adstringens* and 96.7% from *V. divergens*) within two classes: Dothiomycetes and Sordariomycetes. The dominant class in the phylum Ascomycota was Sordariomycetes corresponding to 61% of the isolates obtained from *S. adstringens* and 63% from *V. divergens*. The dominant orders in Sordariomycetes class were Diaporthales (representing 46.6% of isolates from *S. adstringens* and 43.4% from *V. divergens*), Xylariales (10.9% from *S. adstringens* and 9.8% from *V. divergens*), and Glomerellales (2% from *S. adstringens* and 4% from *V. divergens*) (Table 1). The remaining morphotypes belong to the Phylum Basidiomycota, representing 0.7% of isolates from *S. adstringens* and 0.9% from *V. divergens*, within Agaricomycetes class. Seven morphotypes were identified at the family level because they did not cluster with any type species of Xylariaceae family, and possibly belong to three new genera in this family (Table 1; Figure 2).

**Table 1:** Taxonomic classification of endophytic fungi isolated from *Stryphnodendron adstringens* and *Vochysia divergens*.

**Taxa**					
**Class**	**Order**	**Family**	**Genera**	**Species**	**Morphotype**
Dothideomycetes (36% Sa; 33% Vd)	Pleosporales	Lophiostomataceae	*Acrocalyma*	*Acrocalymma medigans*	106
		Didymellaceae	*Didymella*	*Didymella* sp.	32, 16
			*Epicoccum*	*Epicoccum* sp.1	43, 98,
				*Epicoccum* sp.2	61, 100
				*Epicoccum* sp.	45, 97,
		Corynesporascaceae	*Corynespora*	*Corynespora cambrensis*	20
		Pleosporaceae	*Alternaria*	*Alternaria alternata*	33
			*Curvularia*	*Curvularia* sp.	1
		Didymosphaeriaceae	*Paraphaeosphaeria*	*Paraphaeosphaeria* spp.	110, 111
		Thyridariaceae	*Roussoella*	*Roussoella* sp.1	114
	Capnodiales	Mycosphaerellaceae	*Phaeophleospora*	*Phaeophleospora* sp.1,	82
				*Phaeophleospora* sp.2	109, 118
	Botryosphaeriales	Phyllostictaceae	*Phyllosticta*	*Phyllosticta* sp.1	107, 108,
				*Phyllosticta* sp.	116
		Botryosphaeriaceae	*Pseudofusicoccum*	*Pseudofusicoccum* sp.1	3
				*Pseudofusicoccum stromaticcum*	119
			*Neofusicoccum*	*Neofusicoccum brasiliense*	7, 67
			*Lasiodiplodia*	*Lasiodiplodia* spp.	123, 40, 5
Sordariomycetes (61% Sa; 63% Vd)	Xylariales	Diatrypaceae	Diatrypaceae sp.	Diatrypaceae sp.	24
		Xylariaceae	Xylariacea sp.	Xylariacea sp.3,	18
				Xylariacea sp.4,	25, 64, 66
				Xylariacea sp.5	9
		Hypoxylaceae	*Daldinia*	*Daldinia* sp.1	31
			*Hypoxylon*	*Hypoxylon* sp.1	6
		Apiosporaceae	*Nigrospora*	*Nigrospora hainanensis*	28, 89
	Glomerellales	Glomerellaceae	*Colletotrichum*	*Colletotrichum* spp.	62, 71, 90, 117, 47, 76, 84, 86,
				*Colletotrichum siamense*	22, 36, 74, 93, 104
	Sordariales	Coniochaetaceae	*Coniochaeta*	*Coniochaeta nepalica*	68
	Diaporthales	Diaporthaceae	*Diaporthe*	*Diaporthe* spp.	29, 37, 58, 69, 80, 113, 4, 46, 10, 83, 103, 112, 120, 121, 49, 52, 65, 2, 124, 11, 39
				*Diaporthe* sp.1	122, 101, 102
				*Diaporthe* sp.2	12, 13, 14
				*Diaporthe* sp.3	30, 15, 19
				*Diaporthe* sp.4	21, 81
				*Diaporthe* cf. *heveae* 1	56, 60, 91, 92, 63, 70, 54
				*Diaporthe ocoteae*	55
				*Diaporthe schini*	17, 120
	Hypocreales	Nectriaceae	*Fusarium*	*Fusarium* sp.1	26, 38
				*Fusarium* sp.2	27, 44
				*Fusarium pseudocircinatum*	47
				*Fusarium* spp.	23, 48, 57, 51, 53, 59, 85,87,88,
	Amphisphaeriales	Pestalotiopsidaceae	*Pestalotiopsis*	*Pestalotiopsis* sp.1	115
				*Pestalotiopsis* spp.	34, 75, 77, 35
			*Neopestalotiopsis*	*Neopestalotiopsis* sp.	50
Agaricomycetes (0.7% Sa; 0.9% Vd)	Polyporales	Meruliaceae	*Bjerkandera*	*Bjerkandera* sp.	41, 72, 73
		Phanerochaetaceae	*Efibula*	*Efibula* sp.	78

*Vd, Vochysia divergens; Sa, Stryphnodendron adstringens*.

In addition, we also identified 48 taxa (Table S1), from these, 16 isolates did not cluster with any type strain of *Diaporthe* (Figure S11), *Epicoccum* (Figure S14), *Fusarium* (Figure S16), *Hypoxylon* (Figure S17), *Pestalotiopsis* (Figure S23), *Phyllosticta* (Figure S25), *Pseudofusicoccum* (Figure S26), *Roussoella* (Figure S27), and *Phaeophleospora* genera in the phylogeny analysis and may represent new species into these genera, however multilocus and morphological analyses are required for species description.

Nine morphotypes (~3%) did not grow in different culture media after few replications (morphotypes 8, 42, 72, 94, 95, 96, and 99), probably due to the growth-dependence of host tissues, and were characterized only at Kingdom level (fungi).

### Community analysis of endophytic fungi from *V. divergens* and *S. adstringens*

In June 2016, we isolated 777 (IF: 72%) and 339 (IF: 49%) endophytic fungi from *V. divergens* and *S. adstringens*, respectively (Table S1). Concerning of 777 isolates from *V. divergens*, 367 (IF: 72%) were isolated from leaves and 410 (IF: 77%) were isolated from petioles. For the isolates obtained from *S. adstringens* 174 (IF: 45%) were obtained from leaves and 165 (IF: 43%) were obtained from petioles (Tables S1, S2).

**Figure 3:**
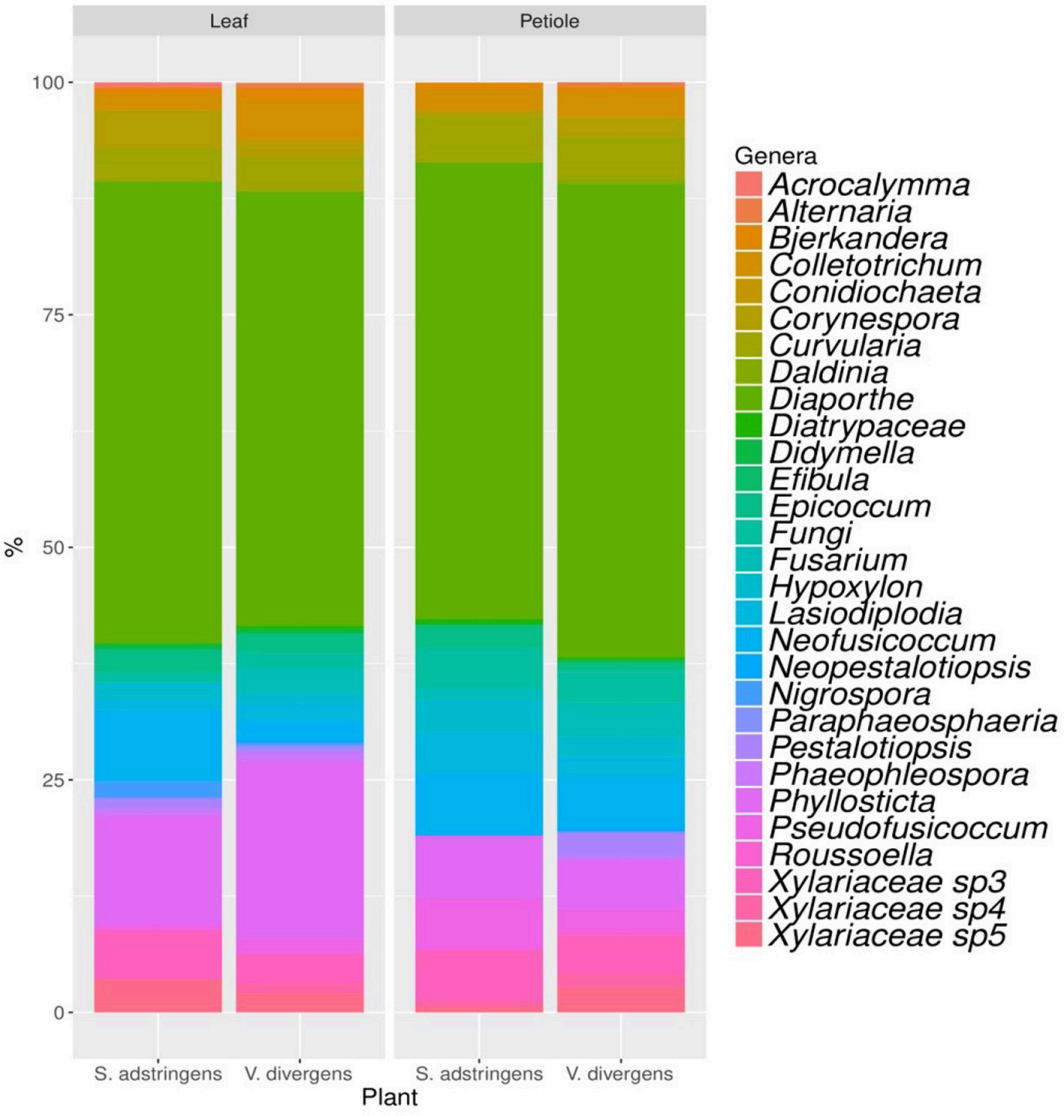
Genera composition of endophytic community obtained from petioles and leaves of *Stryphnodendron adstringens* and *Vochysia divergens*.

The analyses of genera composition showed that *Diaporthe* was the dominant genus in both plants *V. divergens* (51.8% of isolates from petioles and 51.4% from leaves) and *S. adstringens* (49.3% of isolates from petioles and 49.7% from leaves). The second most frequent genus was *Phyllosticta* (5.1 and 19.1% of isolates from petioles and leaves of *V. divergens* and 6.8 and 11.8% from petioles and leaves of *S. adstringens*) followed by *Neofusicoccum* (5.5 and 2.2% from petioles and leaves of *V. divergens* and 6.8 and 7.7% from petioles and leaves of *S. adstringens*). The morphological characteristics from the dominant groups are illustrated in the Figure S1. The genera *Alternaria, Coniochaeta, Neopestalotiopsis*, and *Paraphaeosphaeria* (obtained from leaves and petioles), *Daldinia* and *Efibula* (isolated only from petioles), and *Roussoella* (from leaves) were recovered exclusively from *V. divergens* and *Acrocalymma* were isolated exclusively from leaves of *S. adstringens* (Figure 3).

**Figure 4:**
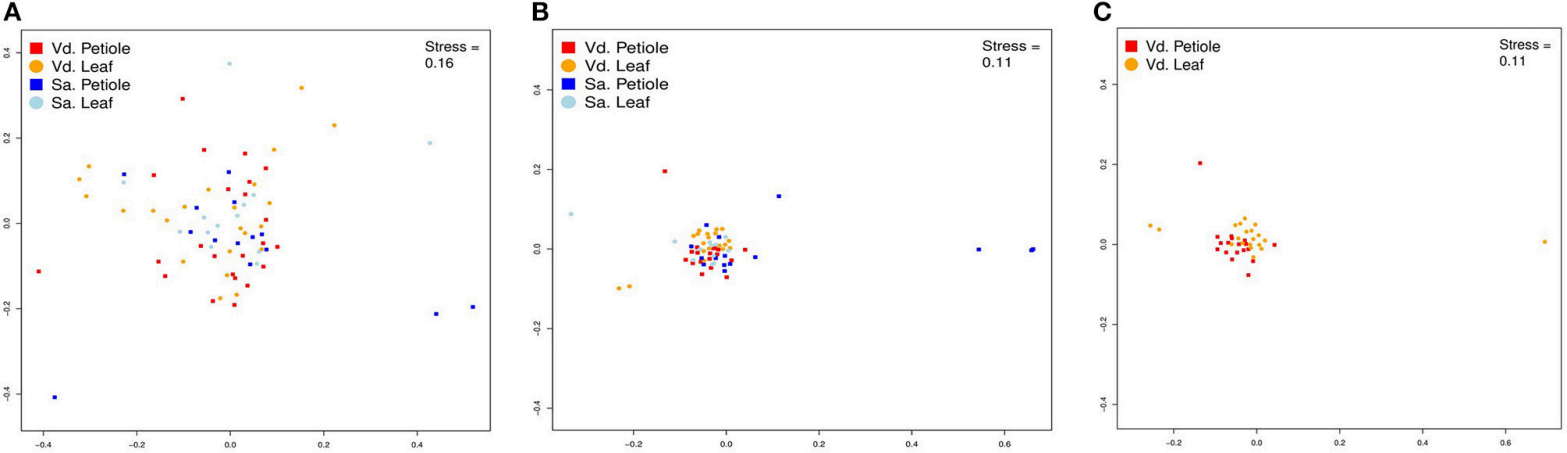
Non-Metric Multidimensional Scaling (nMDS) of plots of fungal endophytic communities from *Vochysia divergens* (Vd) and *Stryphnodendron adstringens* (Sa). **(A)** NMDS plots based in genera level; **(B)** NMDS plots based in morphotype level; **(C)** NMDS plots based in morphotype level.

Community structuring analyses based on genera showed no difference between plant species or plant tissues sampled (Table 2, Figure S28) as seen in PERMANOVA (*p* > 0.05) and nMDS (Figure 4, Tables S3, S4). On the other hand, the analysis of morphotypes composition (a lower taxonomic identification when compared to genera) presented higher abundance and richness for endophytic isolated from *V. divergens* (Table 2; Figures S28–S30) than from *S. adstringens*, and differences in community composition regarding to *V. divergens* plant tissues as shown by PERMANOVA (*p* < 0.05) and nMDS—ANOSIM (Figure 4B). No significance was observed in the diversity indexes (H′, 1-D, J) for genera and morphotypes analyze in *V. divergens* and *S. adstringens* (Table 2).

**Table 2:** Diversity index and descriptive data for morphotypes and genus analyzed.

**Plant**	**Tissues**	**Abundance**	**Richness**	**H′**	**Simpson (1-D)**	**J**
**DIVERSITY INDEX PER MORPHOTYPE**						
*V. divergens*	Petiole	22.56 ± 11.91^*^	14.28 ± 6.35^*^	3.41 ± 1.02	0.85 ± 0.22	1.37 ± 0.04
	Leaf	17.85 ± 11.51^*^	10.60 ± 6.30^*^	2.79 ± 1.25	0.75 ± 0.28	1.33 ± 0.11
*S. adstringens*	Petiole	9.80 ± 5.57	7.13 ± 4.10	2.33 ± 1.23	0.69 ± 0.31	1.38 ± 0.05
	Leaf	13.33 ± 5.60	8.33 ± 3.26	2.66 ± 0.96	0.76 ± 0.25	1.34 ± 0.11
**DIVERSITY INDEX PER GENUS**						
*V. divergens*	Petiole	17.52 ± 11.99	6.35 ± 3.68	1.85 ± 0.88	0.59 ± 0.22	1.12 ± 0.22
	Leaf	13.29 ± 11.24	5.13 ± 3.17	1.64 ± 0.76	0.56 ± 0.21	1.17 ± 0.17
*S. adstringens*	Petiole	10.80 ± 6.79	4.47 ± 2.50	1.51 ± 0.90	0.51 ± 0.28	1.18 ± 0.14
	Leaf	11.27 ± 7.40	4.27 ± 2.55	1.34 ± 0.99	0.44 ± 0.31	1.10 ± 0.17

H′, Shannon-Weaver; J, Pielou evenness;

* *Significate differences between plants*.

### Biological activity of endophytic

#### Screening of antimicrobial activity

Of the 124 morphotypes, 117 isolates were used in the screening for antimicrobial analysis using dual culture, since 7 morphotypes did not grow under the culture conditions used in the present study.

**Figure 5:**
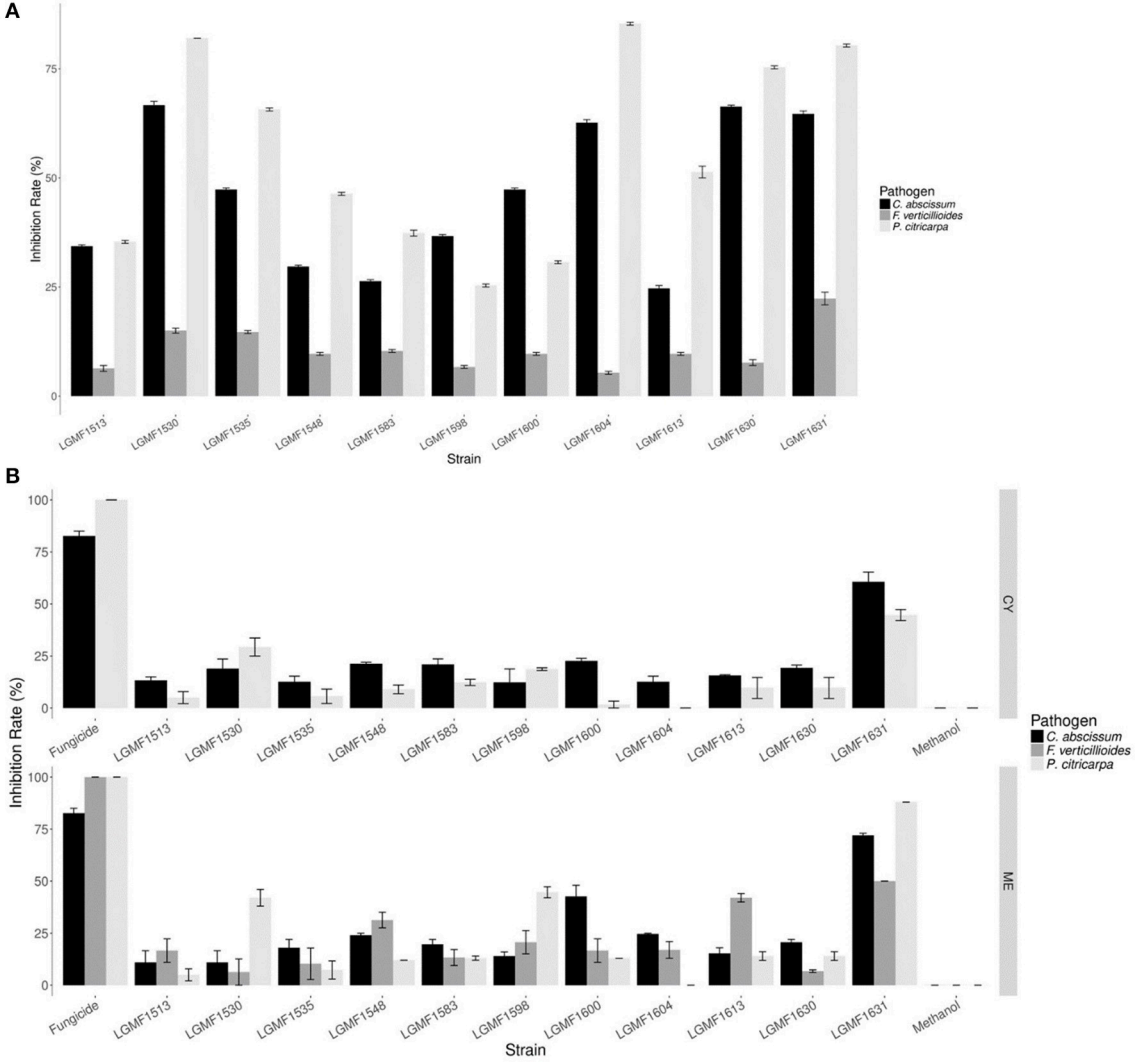
Mean of inhibition growth (in %) of the pathogens *Colletotrichum abscissum* (in black), *Phyllosticta citricarpa* (light gray), and *Fusarium verticillioides* (dark gray) in the dual culture **(A)**. Mean of inhibition growth (in %) of the pathogens *Colletotrichum abscissum* (in black), *Phyllosticta citricarpa* (light gray), and *Fusarium verticillioides* (dark gray) in presence of 100 μL of extracts obtained from cultivation of endophytes in different culture media **(B)**. Fungicide: 100 μL of Carbendazim (0.1 mg/mL) was used from *P. citricarpa* and 100 μL of Derosal (1.0 mg/mL) was used from *C. abscissum* and *F. verticillioides*.

In the screening of antifungal activity among the 117 isolates evaluated *Diaporthe* sp. LGMF1530, *Diaporthe* cf. *heveae* LGMF1631, *Colletotrichum siamense* LGMF1604, *Fusarium* sp. LGMF1630, and *Epicoccum* sp. LGMF1641 inhibited more than 60% the mycelial growth of *P. citricarpa* and *C. abscissum* in the dual culture assay (Figure 5A) and were selected for the extract production. None of the isolates was able to inhibit the fungus *F. verticillioides* (Table S6).

The endophytes were also screened for their antimicrobial activity against clinical pathogens based on the spectrum and intensity of action. The isolates *Phaeophleospora* sp.2 LGMF1513, *Epicoccum* sp.1 LGMF1598, and *Epicoccum* sp.2 LGMF1600 showed high activity (+++) against *S. aureus* (MSSA and MRSA). The isolates *Diaporthe* sp. LGMF1548 and LGMF1614, *Diaporthe* cf. *heveae* LGMF1631, *Fusarium* sp. LGMF1630, and *Hypoxylon* sp.1 LGMF1613 showed broad spectrum with moderate (++) to high (+++) activity against *S. aureus* (MSSA and MRSA), *P. aeruginosa* and *A. baumannii*. The isolates *Neofusicoccum brasiliense* LGMF1535 and *Diaporthe* sp. LGMF1583 showed moderate (++) to high (+++) activity against *S. aureus* (MSSA and MRSA), *P. aeruginosa, A. baumannii*, and *K. Pneumoniae* (KPC) (Table 3; Table S7).

**Table 3:** Selected results of antimicrobial activity of the extracts produced by endophytic fungi in malt extract medium against clinical pathogens.

**Taxon**	**Endophytic strain**		**Pathogens inhibitory zone measurement (mm)**							
		Antibacterial screening^*^	**MRSA**	**MSSA**	***S. maltophilia***	**KPC**	***E. cloacae***	***A. baumannii***	***P. aeruginosa***	***C. albicans***
*Colletotrichum siamense*	LGMF1604	2/55	15 ± 2	9.5 ± 0.7	11 ± 0.7	10 ± 1.4	10 ± 0	0	9.5 ± 0.7	10 ± 2
*Diaporthe* sp.	LGMF1530	2/5	15 ± 2	10 ± 1.4	11 ± 0.7	11 ± 0	14 ± 1.7	0	10 ± 0.7	12 ± 0
	LGMF1548	4/5	14 ± 1.4	**23 ± 0.7**	**17 ± 3.5**	**18 ± 0**	**19 ± 1.4**	0	16 ± 0.7	9.5 ± 0.7
	LGMF1583	5/5	**17 ± 2**	**23 ± 2.8**	14 ± 1.4	14 ± 0.7	14 ± 0.7	0	16 ± 0	**19 ± 1.4**
*Diaporthe* cf hevea	LGMF1631	5/5	10 ± 0	11 ± 1.4	12 ± 0.7	11 ± 1.4	16 ± 2.8	0	0	13 ± 0.7
*Epiccoccum* sp.1	LGMF1598	3/5	11 ± 0.7	11 ± 0.7	**16 ± 0.7**	14 ± 1.4	11 ± 1.4	0	9 ± 0	11 ± 0.7
*Epiccoccum* sp.2	LGMF1600	3/5	9.5 ± 0.7	9.7 ± 0.7	15 ± 1.4	9.5 ± 0.7	10 ± 0	**15 ± 0.7**	10 ± 0	11 ± 1.4
*Neofusicoccum brasiliense*	LGMF1535	5/5	**23 ± 2**	20 ± 0	14 ± 0.7	16 ± 0.7	16 ± 1.4	0	**17 ± 0.7**	14 ± 0
*Fusarium* sp.	LGMF1630	5/5	**5 ± 0.7**	9 **± 0**	0	14 **± 0.1**	12.5 **± 0.7**	0	**0**	5 **± 0.7**
*Hypoxylon* sp.	LGMF1613	4/5	**5 ± 0.9**	10 **± 0**	13 **± 2**	**0**	13 **± 2**	0	**0**	6 **± 8**
*Phaeopleospora* sp.	LGMF1513	2/5	**5 ± 0.7**	11 **± 0.2**	12 **± 0.7**	12 **± 0.7**	11 ± 0	11 **± 0.1**	**0**	13 **± 0.2**

* *The antibacterial screening refer to the number of clinical pathogens inhibited/the number of pathogens evaluated, the full data is in supplementary information Table S6. MRSA, Methicillin-resistant Staphylococcus aureus; MSSA, methicillin-sensitive S. aureus; S. maltophilia, Stenotrophomonas maltophilia; KPC, Klebissiella pneumoniae producer of KPC; E. cloacae VIM, Enterobacter cloacae producer of VIM; A. baumannii, Acinetobacter baumannii; P. aeruginosa, Pseudomonas aeruginosa; C. albicans, Candida albicans. All the highest inhibition zone for each pathogen are underlined. The best result for each pathogen is in bold. 0, No inhibition zone*.

The above-mentioned isolates were selected for secondary metabolites production in different culture media.

#### Antifungal activity of extracts produced by the selected endophytes against phytopathogens

Eleven endophytic fungi were cultured in two different media, and seven of them produced extracts that inhibited the mycelial growth of *P. citricarpa* (Figure 5B), especially the extracts from strain *Diaporthe* cf. *heveae* LGMF1631 cultured in ME (IG: 88%) and CY (IG:50%) media, and extracts of *Diaporthe* sp. LGMF1530 (IG:42%) and *Epicoccum* sp.1 LGMF1598 (IG:45%). Extracts from four isolates inhibited the mycelial growth of *C. abscissum* (Figure 5B), highlighting the extracts from *Diaporthe* cf. *heveae* LGMF1631 produced in ME medium (Inhibition growth—IG: 72%) and in CY medium (IG: 59%) and the extract from *Epicoccum* sp.2 LGMF1600 in ME medium (IG: 40%). In the other hand, the extracts from *Diaporthe* cf. *heveae* LGMF1631 (IG: 50%) and *Hypoxylon* sp.1 LGMF1613 (IG: 45%) showed moderate activity against *F. verticillioides (*Figure 5B).

### Antimicrobial activity of extracts produced by the selected endophytes against clinical pathogens

All extracts inhibited the growth of at least one clinical pathogen and the best results were obtained with extracts produced in ME medium (Table S8). The extracts from *Diaporthe* sp. LGMF1548 caused the highest inhibition zone against four clinical pathogens: *S. aureus* (MSSA) (23 mm), *S. maltophilia* (16 mm), *K. pneumoniae* (KPC) (18 mm), and *E. cloacae* (18 mm). Extract produced by *Epicoccum* sp.1 LGMF1598 showed equivalent inhibition zone against *S. maltophilia* (16 mm), and, curiously, it was the only extract that showed high activity against *A. baumannii* (15 mm) (Table 3; Table S8). The extract produced by *N. brasiliense* LGM1535 presented the largest inhibition zone (23 mm) against Methicillin-resistant *S. aureus* (MRSA) growth. The second best result (17 mm) against MRSA was observed in the treatment with the extract of the isolate *Diaporthe* sp. LGMF1583, that also showed the highest inhibition zone against *C. albicans* (19 mm) and *P. aeruginosa* (17 mm). Table 3 showed only the highest inhibition values against each pathogen, the complete data is in the Table S8.

## Discussion

In order to verify if the peculiar environmental conditions found in Pantanal and Cerrado can influence the endophytic community in these biomes, we evaluated the diversity and composition of endophytic fungi in two resilient and dominant plants found in these regions, *V. divergens* of the Pantanal and *S. adstringens* of the Cerrado.

Our data revealed that *V. divergens* and *S. adstringens* are hosts of high diversity of endophytic fungi, belonging to 23 known genera, and three possible new genera in the Xylariaceae family. The number of isolates (genera and morphotypes) found in our study is larger than previous reports (Carvalho et al., 2012; Hokama et al., 2017), which may be a result of the number of evaluated plants or the region where the collections were carried out. Hokama et al. (2017) studied the endophytes of *V. divergens* found in another region of the Pantanal, denominated Rio Negro. The authors also obtained isolates of genera *Colletotrichum, Diaporthe*, and *Phyllosticta* and strains belonging to possible new genera of the family Xylariaceae. However, in the present work we report for the first time the genera *Acrocalymma, Alternaria, Bjerkandera, Coniochaeta, Corynespora, Curvularia, Didymella, Efibula, Epicoccum, Fusarium, Hypoxylon, Lasiodiplodia, Neopestalotiopsis, Paraphaeosphaeria, Phaeophleospora*, and *Roussoella* as endophytes of *V. divergens* (Table S1; Figures S1–S3, S6–S8, S11–S13, S15–S17, S19, S21, S23, S26). With the results obtained in this work, we are increasing knowledge about the endophytic community of these important medicinal plants.

Considering the limitations of the ITS sequences to identify some isolates at the species level, we analyzed the endophytic community using two matrix data, based on the classification of genera and morphotypes. As expected, the lower taxonomic level (morphotype) resulted in a higher resolution in the community structure. Therefore, correct identification at lower levels is of great importance for the assessment of community structure (Singh et al., 2017). The variation of the composition of the morphotypes among the analyzed hosts, *V. divergens* and *S. adstringens*, may be associated with the analyzed plant or even the environmental conditions. Pantanal plants can be severely damaged by flooding, however, *V. divergens* is highly adapted and only a short leaf falls period is reported (Da cunha Nunes et al., 2000). In this context, the endophytic community of the leaves of *V. divergens* do not change seasonally, providing a longer interaction between the endophytic community and the plant, both of which are adapted to the environmental conditions. In contrast, the leaves of plants found in the Cerrado can be destroyed by flames or damaged by the hot air flow (Silvério et al., 2015; Dodonov et al., 2017), which results in a shorter interaction time between the community and the plant. In addition, the region of analysis in the Cerrado is severely affected by anthropological action. Certainly, these characteristics impact the endophytic community in these biomes and may play a role in the differences observed for the composition of the morphotypes between *V. divergens* and *S. adstringens*. The community also differs among the tissues from *V. divergens* analyzed, not easily visible in the results of nMDS, but confirmed by ANOSIM (Figure 4; Tables S4, S5). The main difference in the community structure comes from a higher abundance and richness of endophytes in petioles, which may be result of a specialized endophytic microbiota (Persoh, 2015; Correia et al., 2017).

*Diaporthe* was the dominant genus in both plants (Figure 3; Table S1). This genus has also been described as dominant in *S. adstringens* by Carvalho et al. (2012) and is widely distributed in different plants of several biomes, such as the Pantanal (Hokama et al., 2017; Pietro-Souza et al., 2017), Mangrove forest (de Souza Sebastianes et al., 2013), Atlantic Rainforest (Correia et al., 2017), Cerrado and Caatinga (Ferreira et al., 2012; Lisboa et al., 2013). The ability of *Diaporthe* species to colonize different plants may result from the produced metabolites that bypass the plants defenses, or even by acting on host development and defense against plant pathogens (Nicoletti and Fiorentino, 2015; Santos et al., 2016; Tahir et al., 2017). Associated with the high frequency of isolation, we also found new species of *Diaporthe*, named as *Diaporthe* sp.1 (clade 1), *Diaporthe* sp. 2 (clade 2), (Figure S11). Gomes et al. (2013) also reported a large number of *Diaporthe* isolates as new species, suggesting medicinal plants in Brazil as a repository for this genus. The description of new species of *Diaporthe* is carried out based on the polyphasic approach, and will be carried out in a future study.

In order to associate economic value with biodiversity, bioprospecting studies have reported the biotechnological potential of endophytes against a large number of pathogens (Schulz et al., 2002; Gathage et al., 2016; Larran et al., 2016; Li et al., 2016; Hokama et al., 2017; Parpinelli et al., 2017). In this context, we investigated the antimicrobial potential of the endophytes of *V. divergens* and *S. adstringens*. Extracts produced by *Diaporthe* cf. *heveae* LGMF1631 inhibited almost 90% the mycelial growth of *P. citricarpa* the causal agent of Citrus Black Spot (CBS), a disease that affects fruits and leaves of several citrus hosts (Kotzé, 1996). The control of CBS in Brazil is based on fungicides applications, such as Derosal, and on cultural management (Kotzé, 1996; Baldassari et al., 2006). However in fruits destined to the production of juice for export, the application of the fungicide Derosal® is restricted (EPA, 2012; Fundecitrus, 2015). *Diaporthe* cf. *heveae* LGMF1631 also caused excellent inhibition (72%) of the mycelial growth of *C. abscissum*, the epidemiological agent of Post-bloom Fruit Drop (PFD) (Pinho et al., 2015; Silva et al., 2016). In favorable climatic conditions, frequent rainy days during the bloom period, PFD can drastically reduce citrus production, reaching approximately 80% (Lima et al., 2011). Thus, the use of secondary metabolites produced by *Diaporthe* endophytic strains, in a biological control scenario, may be an alternative to reduce the use of fungicides in the control of citrus diseases such as CBS and PFD (Santos et al., 2016; Tonial et al., 2017).

The antimicrobial tests also revealed a high potential of endophytes to produce active secondary metabolites with broad spectrum against clinical pathogens, especially those produced by *N. brasiliense* LGMF1535, *Diaporthe* sp. LGMF1548 and LGMF1583 (Table 3). Significant results were obtained against MRSA (a gram-positive) and KPC (a gram-negative), two bacteria with resistance to β-lactam antibiotics, cephalosporins, monobactams, and carbapenems, respectively (Queenan and Bush, 2007). There is an urgency in finding new compounds to contain the global pandemic involving resistance to gram-positive pathogens to several antibiotics (including MRSA as the main species), and which is currently one of the greatest threats to human health. In patients with bloodstream infection, for example, the range of antibiotic resistance can reach up to 82% (CDC, 2013; Ventola, 2015; World Helth Organization, 2018).

The species belonging to the genera *Diaporthe* and *Neopestalotiopsis* are described as producing a large number of compounds with activity against several clinical pathogens, such as *Escherichia coli, S. aureus, Enterococcus hirae, Micrococcus luteus*, and *Salmonella typhi* (Specian et al., 2012). Thus, the exploration of metabolites produced by endophytes belonging to these genera and obtained from unexplored environments may represent a source for new compounds (Monciardini et al., 2014), and the extracts of the strains *N. brasiliense* LGMF1535, *Diaporthe* sp. LGMF1548 and LGMF1583 will be further explored in the chemical characterization.

## Conclusions

This was the first report comparing the endophytic community found in medicinal plants from the Pantanal and Cerrado biomes. Our data revealed high diversity of endophytes from both biomes, and the structure of communities at morphotype level was different between the two medicinal plants, suggesting that host species and the environmental conditions may affect the endophytic diversity. *Diaporthe* was the dominant genus found as endophyte in both plants. In addition, the isolates belonging to *Diaporthe* genus produced secondary metabolites with wide spectrum acting against different phytopathogens and microorganisms of clinical importance. Future studies will be conducted on the isolation and identification of bioactive compounds, and also a multilocus analysis will be performed for the description of the new species.

## Author contributions

All authors listed have made a substantial, direct and intellectual contribution to the work, and approved it for publication.

### Conflict of interest statement

The authors declare that the research was conducted in the absence of any commercial or financial relationships that could be construed as a potential conflict of interest.
