# Supplementary material for: Bioprospecting and Structure of Fungal Endophyte Communities Found in the Brazilian Biomes, Pantanal, and Cerrado
Source: Front Microbiol. 2018 Jul 24;9:1526. doi: 10.3389/fmicb.2018.01526 (PMC6066559; doi:10.3389/fmicb.2018.01526)
Supplement: Supplementary file 1 [file Data_Sheet_1.docx]

Supplementary Material

# Bioprospecting and structure of fungal endophyte communities found in the Brazilian biomes, Pantanal and Cerrado

Sandriele Aparecida Noriler^1^, Daiani Cristina Savi^2^, Rodrigo Aluizio^2^, Angela Maria Palacio-Cortés^3^, Yvelise Maria Possiede^4^, Chirlei Glienke^2*^

^1^Federal University of Paraná, Department of Basic Pathology, Curitiba, Brazil. ^2^Federal University of Paraná, Department of Genetics, Curitiba, PR, Brazil. ^3^ Federal University of Paraná, Department of Zoology, Curitiba, PR, Brazil. ^4^Federal University of Mato Grosso do Sul, Department of Biology, Campo Grande, MS, Brazil.

*Corresponding author: Chirlei Glienke, +554133611562, ch.glienke@gmail.com

**Running title:** Endophytic biodiversity of the Pantanal and Cerrado Biomes

| **Contents** | **Page** | |
| --- | --- | --- |
| **Table S1.** Endophytic fungi isolated from medicinal plants Stryphnodendron adstringens (Sa) and Vochysia divergens (Vd): Identification and number of isolates obtained for each morphotype, source (leave and/or petiole), plant species, selected strain code, Genbank accession number of ITS sequence and phylogeny analysis  **Table S2-**Indicator Species Values table for morphotypes. Highlighted values indicate significant representatives of a group. | | 6  24 |
| **Table S3-**Permanova results for morphotypes. | | 26 |
| **Table S4**-ANOSIMs results per morphotype comparing plants and tissues. | | 26 |
| **Table S5-**Screening of antimicrobial activity of the endophytic fungi against phytopathogens. | | 27 |
| **Table S6-**Screening of antimicrobial activity of the endophytic fungi against clinical pathogens. | | 30 |
| **Table S7**-Extract yield performed in 250 mL in two culture media: ME (Malte extract) and CY (Czapeck). | | 33 |
| **Table S8**-Inhibition zone of the crud extracts produced by endophytic fungi against clinical pathogens. | | 35 |
| **Figure S1**- Morphological aspect of the dominant endophytic fungi found in this study. For details on the source (plant species), see Table 1. A: *Diaporthe schini* (morphotype 17; macro and micromorphological aspect), B: *Phyllosticta* sp. (morphotype 116), C: *Neofusicoccum brasiliense* (morphotype 7). | | 36 |
| **Figure S2-** Bayesian phylogenetic tree based on ITS partial sequence of LGMF1605 (bold) identified as *Acrocalymma medicaginis* and sequences of all accepted species from *Acrocalymma* genus. The data matrix had 25 taxa and 465 characters. The tree was rooted to *Massarina* *eburnea* (AF383959). Scale bar shows 0.03 changes and Bayesian posterior probability values are indicated at the nodes. T: type strain. | | 37 |
| **Figure S3-** Bayesian phylogenetic tree based on ITS partial sequence of LGMF1550 (bold) identified as *Alternaria* sp., and sequence of all species allocated in *Alternaria* sect. *Alternaria*. The data matrix had 12 taxa and 500 characters. The tree was rooted to *Alternaria* *alternantherae* (KC584179). Scale bar shows 0.003 changes and Bayesian posterior probability values are indicated at the nodes. T: type strain. | | 38 |
| **Figure S4-** Bayesian phylogenetic tree based on ITS partial sequence of LGMF1627, LGMF1578 and LGMF1579 (bold) identified as *Bjerkandera* sp. and sequences of all accepted species from *Bjerkandera*. The tree was rooted to *Phanerochaete chrysosporiu* (HQ188436). Scale bar shows 0.03 changes and Bayesian posterior probability values are indicated at the nodes. T: type strain. | | 39 |
| **Figure S5-** Bayesian phylogenetic tree based on ITS partial sequence of LGMF1559, LGMF1580 LGMF1586, LGMF1621, LGMF1625, LGMF1636, LGMF1595 and LGMF1604 (bold) identified as *Colletotrichum gloeosporioides* sensu lato and sequences of all species of *Colletotrichum* *gloeosporioides* species complex. The data matrix had 60 taxa and 407 characters. The tree was rooted to *Colletotrichum boninense* (JX010292). Scale bar shows 0.03 changes and Bayesian posterior probability values are indicated at the nodes. T: type strain. | | 40 |
| **Figure S6**- Bayesian phylogenetic tree based on ITS partial sequence of LGMF1571, LGMF1577, LGMF1592, and LGMF1522 (bold) identified as *Colletotrichum* *boninense* sensu lato and sequences of all accepted species from *Colletotrichum* *boninense* species complex. The data matrix had 26 taxa and 312 characters. The tree was rooted to *Colletotrichum gloeosporioides* (JQ005152). Scale bar shows 0.002 changes and Bayesian posterior probability values are indicated at the nodes. T: type strain. | | 41 |
| **Figure S7-** Bayesian phylogenetic tree based on ITS partial sequence of LGMF1575 (bold) identified as *Coniochaeta nepalica*. The data matrix had 30 taxa and 488 characters. The tree was rooted to *Phialemonium obovatum* (HE610365). Scale bar shows 0.04 changes and Bayesian posterior probability values are indicated at the nodes. T: type strain. | | 42 |
| **Figure S8**- Bayesian phylogenetic tree based on ITS partial sequence of LGMF1619 (bold) identified as *Corynespora cambrensis* and sequences of all accepted species from *Corynespora* genus. The data matrix had 11 taxa and 409 characters. The tree was rooted to *Cyclothyriella rubronotata* (KX650544). Scale bar shows 0.04 changes and Bayesian posterior probability values are indicated at the nodes. T: type strain. | | 43 |
| **Figure S9**- Bayesian phylogenetic tree based on ITS partial sequence of LGMF1533 (bold) identified as *Curvularia* sp. and sequences of all accepted species from *Curvularia* genus. The data matrix had 47 taxa and 491 characters. The tree was rooted to *Setosphaeria turcica* (HF934950). Scale bar shows 0.02 changes and Bayesian posterior probability values are indicated at the nodes. T: type strain. | | 44 |
| **Figure S10-** Bayesian phylogenetic tree based on ITS partial sequence of LGMF1623 (bold) identified as *Daldinia* sp. and sequences of all accepted species from *Daldinia* genus. The data matrix had 69 taxa and 522 characters. The tree was rooted to *Hypoxylon fragiforme* (AY616690). Scale bar shows 0.02 changes and Bayesian posterior probability values are indicated at the nodes. T: type strain. | | 45 |
| **Figure S11**- Bayesian phylogenetic tree based on ITS partial sequence of LGMF1610, LGMF1612, LGMF1614, 1538, LGMF1539, LGMF1540, LGMF1615, LGMF1616, LGMF1541, LGMF1618, LGMF1620, LGMF1548, 1549, LGMF1554, LGMF1629, LGMF 1561, LGMF1563, LGMF1631, LGMF1632, LGMF 1565, LGMF1567, LGMF 1569, LGMF 1633, LGMF1573, LGMF1576, LGMF1635, LGMF1583, LGMF1639, LGMF1593, LGMF1594, LGMF1601, LGMF1602, LGMF1508, LGMF1517, LGMF1606, LGMF1525, LGMF1526, LGMF1530, LGMF1531, LGMF1532, and LGMF1526 (bold) sequences of all accepted species from *Diaporthe* genus. The data matrix had 383 taxa and 537 characters. The tree was rooted to *Diaporthella* *corylina* (KC343004). Scale bar shows 0.04 changes and Bayesian posterior probability values are indicated at the nodes. T: type strain. | | 46 |
| **Figure S12-** Bayesian phylogenetic tree based on ITS partial sequence of LGMF1617 (bold) identified as *Didymella* sp. and sequences of all accepted species from *Didymella*. The data matrix had 60 taxa and 497 characters. The tree was rooted to *Epicoccum* *nigrum* (FJ426996). Scale bar shows 0.003 changes and Bayesian posterior probability values are indicated at the nodes. T: type strain. | | 51 |
| **Figure** **S13**- Bayesian phylogenetic tree based on ITS partial sequence of LGMF1581 (bold) identified as *Efibula* sp. and sequences of all accepted species from *Efibula* genus. The data matrix had 8 taxa and 615 characters. The tree was rooted to *Ceraceomyces* *serpens* (KP135030). Scale bar shows 0.02 changes and Bayesian posterior probability values are indicated at the nodes. T: type strain R: representative strain. | | 52 |
| **Figure S14-** Bayesian phylogenetic tree based on ITS partial sequence of LGMF1603, LGMF1628, LGMF1641, LGMF1598 and LGMF1557 (bold) identified as *Epicoccum* sp. and sequences of all accepted species from *Epicoccum* genus The data matrix had 35 taxa and 455 characters. The tree was rooted to *Didymella americana* (FJ426974). Scale bar shows 0.002 changes and Bayesian posterior probability values are indicated at the nodes. T: type strain. | | 53 |
| **Figure S15-** Bayesian phylogenetic tree based on ITS partial sequence of LGMF1544 (bold) identified as *Diatrypaceae* sp. and sequences of all accepted genera from *Diatrypaceae* family The data matrix had 110 taxa and 641 characters. The tree was rooted to *Whalleya microplaca* (AJ4390420). Scale bar shows 0.002 changes and Bayesian posterior probability values are indicated at the nodes. T: type strain. | | 54 |
| **Figure S16-** Bayesian phylogenetic tree based on EF partial sequence of LGMF1546, LGMF1553, LGMF1590, LGMF1547 and LGMF1558 (bold) identified as *Fusarium* sp. and sequences of all accepted species from *Fusarium* genus The data matrix had 97 taxa and 680 characters. The tree was rooted to *Fusairum oxysporum* (NRRL22902). Scale bar shows 0.008 changes and Bayesian posterior probability values are indicated at the nodes. T: type strain. | | 55 |
| **Figure S17**- Bayesian phylogenetic tree based on ITS partial sequence of LGMF1613 (bold) identified as *Hypoxylon* sp. and sequences of all accepted species from *Hypoxylon* genus The data matrix had 35 taxa and 369 characters. The tree was rooted to *Nemania* *serpens* (FN428829). Scale bar shows 0.04 changes and Bayesian posterior probability values are indicated at the nodes. T: type strain. | | 56 |
| **Figure S18**- Bayesian phylogenetic tree based on ITS partial sequence of LGMF1609, LGMF1555 and LGMF1534 (bold) identified as of *Lasiodiplodia* sp. and sequences of all accepted species from *Lasiodiplodia* genus The data matrix had 34 taxa and 401 characters. The tree was rooted to *Spencermartinsia viticola* (AY905554). Scale bar shows 0.004 changes and Bayesian posterior probability values are indicated at the nodes. T: type strain. | | 57 |
| **Figure S19**- Bayesian phylogenetic tree based on ITS partial sequence of LGMF1535 and LGMF1634 (bold) identified as *Neofusicoccum brasiliense* and *Neofusicoccum* sp. The data matrix had 30 taxa and 505 characters. The tree was rooted to *Botryosphaeria dothidea* (AY259092). Scale bar shows 0.005 changes and Bayesian posterior probability values are indicated at the nodes. T: type strain. | | 58 |
| **Figure S20**- Bayesian phylogenetic tree based on ITS partial sequence of LGMF1562 (bold) identified as *Neopestalotiopsis* sp. and sequences of all accepted species from *Neopestalotiopsis* genus The data matrix had 59 taxa and 480 characters. The tree was rooted to *Pestalotiopsis trachicarpicola* (JQ845947). Scale bar shows 0.005 changes and Bayesian posterior probability values are indicated at the nodes. T: type strain. | | 59 |
| **Figure S21**- Bayesian phylogenetic tree based on ITS partial sequence of LGMF1622 and LGMF1591 (bold) identified as *Nigrospora hainanensis* and sequences of all accepted species from *Nigrospora* genus. The data matrix had 27 taxa and 500 characters. The tree was rooted to *Arthrinium malaysianum* (KX986096)*.* Scale bar shows 0.02 changes and Bayesian posterior probability values are indicated at the nodes. T: type strain. | | 60 |
| **Figure S22**- Bayesian phylogenetic tree based on ITS partial sequence of LGMF1515 and LGMF1516 (bold) identified as *Paraphaeophaeria* sp. and sequences of all accepted species from *Paraphaeophaeria* genus. The data matrix had 19 taxa and 488 characters. The tree was rooted to *Paraconiothyrium archidendri* (JX496049). Scale bar shows 0.02 changes and Bayesian posterior probability values are indicated at the nodes. T: type strain. | | 61 |
| **Figure S23-** Bayesian phylogenetic tree based on ITS partial sequence of LGMF1519, LGMF1552, LGMF1551, LGMF1637 and LGMF1638 (bold) identified as *Pestalotiopsis* sp. and sequences of all accepted species from *Pestalotiopsis* genus The data matrix had 84 taxa and 376 characters. The tree was rooted to *Neopestalotiopsis* *saprophytica* (KY082708). Scale bar shows 0.02 changes and Bayesian posterior probability values are indicated at the nodes T: type strain. | | 62 |
| **Figure S24-** Bayesian phylogenetic tree based on ITS partial sequence of LGMF1584, LGMF1513 and LGMF1524 (bold) identified as *Phaeophleospora* sp. and sequences of all accepted species from *Phaeophleospora* genus. The data matrix had 27 taxa and 496 characters. The tree was rooted to *Pseudoramichloridium henryi* (KF901535). Scale bar shows 0.08 changes and Bayesian posterior probability values are indicated at the nodes. T: type strain. | | 63 |
| **Figure** **S25**- Bayesian phylogenetic tree based on ITS partial sequence of LGMF1584, LGMF1513 and LGMF1524 (bold) identified as *Phyllosticta* sp. and sequences of all accepted species from *Phyllosticta* genus. The data matrix had 69 taxa and 358 characters. The tree was rooted to *Phyllosticta mimusopisicola.* Scale bar shows 0.02 changes and Bayesian posterior probability values are indicated at the nodes. T: type strain. | | 64 |
| **Figure S26**- Bayesian phylogenetic tree based on ITS partial sequence of LGMF1611 and LGMF1608 (bold) identified as *Pseudofusicoccum stromaticcum* and *Pseudofusicoccum* sp. and sequences of all accepted species from *Pseudofusicoccum* genus. The data matrix had 11 taxa and 519 characters. The tree was rooted to *Endomelanconiopsis microspore* (KF766165). Scale bar shows 0.008 changes and Bayesian posterior probability values are indicated at the nodes. T: type strain. | | 65 |
| **Figure S27-** Bayesian phylogenetic tree based on ITS partial sequence of LGMF1518 (bold) identified as *Roussoella* sp. and sequences of all accepted species from *Roussoella* genus. The data matrix had 23 taxa and 388 characters. The tree was rooted to *Neoroussoella bambusae* (KJ474827). Scale bar shows 0.06 changes and Bayesian posterior probability values are indicated at the nodes. T: type strain. | | 66 |
| **Figure S28-**Boxplot graphic showing median, quartiles and outliers for diversity indexes, abundance and richness A) in Morphotype and B) in Genera. Note: Vd for *Vochysia divergens* and Sa for *Stryphnodendron adstringens*. | | 67 |
| **Figure S29-**Rarefaction curve of richness and abundance per morphotype of endophytic fungi associated with *Vochysia* *divergens* (blue), *Stryphnodendron* *adstringens* (green) and both (grey). | | 68 |
| **Figure S30-**Rarefaction curve of richness and abundance per genus of endophytic fungi associated with *Vochysia divergens* (blue), *Stryphnodendron adstringens* (green) and both (grey). | | 69 |

Table S1. Endophytic fungi isolated from medicinal plants *Stryphnodendron adstringens* (Sa) and *Vochysia divergens* (Vd): Identification and number of isolates obtained for each morphotype, source (leave and/or petiole), plant species, selected strain code, Genbank accession number of ITS sequence and phylogeny analysis

| **Identification** | **Macromorphological characteristics** | **Morphotypes** | **Number of isolates** | **Source** | **Plant** | **Strain** | **ITS Genbank** | **Suplementary material** |
| --- | --- | --- | --- | --- | --- | --- | --- | --- |
| *Acrocalymma medicaginis* | Mycelium powdery, slow growing, colony colour: grey, from above, black and red, from reverse, moderate growing. | 106 | 1 | Lf | Sa | LGMF1605 | MG976365 | Figure S2 |
| *Alternaria section alternata* | Aerial mycelium, colony colour: gray reddish brown, from above, gray reverse, moderate growing. | 33 | 4 | Lf / Pt | Vd | LGMF1550 | MG976387 | Figure S3 |
| *Bjerkandera* sp. | Mycelium fluffy, colony colour : white from above and from reverse, moderate growing. | 72 | 3 | Lf / Pt | Vd / Sa | LGMF1578 | MG976388 | Figure S4 |
| *Bjerkandera* sp. | Mycelium fluffy, colony colour: white, from above, and black from reverse, moderate growing. | 73 | 4 | Lf / Pt | Vd / Sa | LGMF1579 | MG976430 | Figure S4 |
| *Bjerkandera* sp. | Mycelium fluffy, colony colour: white, from above, and gray, from reverse, moderate growing. | 41 | 1 | Pt | Vd / Sa | LGMF1627 | MG976343 | Figure S34 |
| *Colletotrichum siamense* | Mycelium fluffy, colony colour: white, from above, and creamy yellow from reverse, fast-growing. | 74 | 1 | Lf | Vd / Sa | LGMF1636 | MG976384 | Figure S5 |
| *Colletotrichum gloeosporioides* sensu lato | Mycelium fluffy, colony colour: white to orange-colored, from above and reverse, fast-growing. | 47 | 4 | Lf / Pt | Vd / Sa | LGMF1559 | MG976380 | Figure S5 |
| *Colletotrichum siamense* | Dense mycelium, colony colour: whitish to gray, from above, and orange, from reverse, fast-growing. | 36 | 5 | Lf / Pt | Vd / Sa | LGMF1625 | MG976438 | Figure S5 |
| *Colletotrichum* *siamense* | Dense mycelium, colony colour: whitish to yellow, from above and creamy yellow from reverse, fast-growing. | 22 | 7 | Lf / Pt | Vd / Sa | LGMF1621 | MG976428 | Figure S5 |
| *Colletotrichum gloeosporioides* sensu lato | Mycelium irregular, colony colour: white to yellow-colored reddish, from above and reverse, fast-growing. | 76 | 1 | Pt | Vd / Sa | LGMF1580 | MG976393 | Figure S5 |
| *Colletotrichum gloeosporioides* sensu lato | Mycelium irregular, colony colour: white to orangish colored, from above and reverse, fast-growing. | 84 | 4 | Lf / Pt | Vd / Sa | LGMF1586 | MG976395 | Figure S5 |
| *Colletotrichum gloeosporioides* sensu lato | Mycelium irregular, colony colour: flat and white to grayish colored, from above and reverse, fast-growing. | 86 | 4 | Lf / Pt | Vd / Sa | LGMF1588 | MG976398 | Figure S5 |
| *Colletotrichum siamense* | Mycelium fluffy, colony colour: white to yellow-colored, from above, and yellow from reverse, fast-growing. | 104 | 1 | Lf | Vd / Sa | LGMF1604 | MG976424 | Figure S5 |
| *Colletotrichum siamense* | Dense mycelium, colony colour: whitish to gray, from above, and yellow, from reverse, fast-growing. | 93 | 1 | Lf | Vd / Sa | LGMF1595 | MG976407 | Figure S5 |
| *Colletotrichum boninense* sensu lato | Dense mycelium, colony colour: light grey, from above, and dark yellow, from reverse, fast-growing. | 90 | 1 | Lf | Vd / Sa | LGMF1592 | MG976401 | Figure S6 |
| *Colletotrichum* | Dense mycelium, colony colour: cream, from above and light grey from reverse, fast-growing. | 71 | 1 | Pt | Vd / Sa | LGMF1577 | MG976389 | Figure S6 |
| *boninense* sensu lato | Dense mycelium, colony colour: white to yellow, from above and light grey, from reverse, fast-growing. | 62 | 5 | Lf / Pt | Vd / Sa | LGMF1571 | MG976386 | Figure S6 |
| *Colletotrichum boninense* sensu lato | Dense mycelium, colony colour: whitish‐orange, from above and white, from reverse, fast-growing. | 117 | 1 | Lf | Vd / Sa | LGMF1522 | MG976371 | Figure S6 |
| *Coniochaeta* *nepalica* | Aerial mycelium, colony colour: white, from above and cream to reddish, from reverse, slow-growing. | 68 | 3 | Lf / Pt | Vd | LGMF1575 | MG976422 | Figure S7 |
| *Corynespora cambrensis* | Fluffy mycelium, colony colour: dark blackish brown, from above and reverse, moderate-growing. | 20 | 22 | Lf / Pt | Vd / Sa | LGMF1619 | MG976352 | Figure S8 |
| *Curvularia* sp. | Fluffy mycelium, colony colour: blackish brown, from above and dark, from reverse, fast-growing. | 1 | 45 | Lf / Pt | Vd / Sa | LGMF1533 | MG976426 | Figure S9 |
| *Daldinia* sp.1 | Dense mycelium, colony colour: grey with reddish in patches, from above, and brownish, from reverse, moderate-growing. | 31 | 2 | Pt | Vd | LGMF1623 | MG976413 | Figure S10 |
| *Diaporthe* sp. | Aerial mycelium, colony colour: white from above and black to yellow from reverse, fast-growing. | 2 | 29 | Lf / Pt | Vd / Sa | LGMF1610 | MG976330 | Figure S11 |
| *Diaporthe* sp. | Aerial mycelium, colony colour: white with greenish in patches from above and white to dark green from reverse, fast-growing. | 103 | 2 | Lf | Vd / Sa | LGMF1508 | MG976338 | Figure S11 |
| *Diaporthe* sp. | Aerial mycelium, colony colour: white with yellow in patches from above and cream to brown from reverse, fast-growing. | 112 | 3 | Lf | Vd / Sa | LGMF1517 | MG976349 | Figure S11 |
| *Diaporthe* sp. | Aerial mycelium, colony colour: white with yellow in patches from above and cream to brown from reverse, fast-growing | 121 | 1 | Lf | Vd / Sa | LGMF1530 | MG976350 | Figure S11 |
| *Diaporthe* sp. | Powdery mycelium, colony colour: light grey with dark grey in patches from above and dark grey from reverse, fast-growing | 122 | 1 | Lf | Vd / Sa | LGMF1531 | MG976351 | Figure S11 |
| *Diaporthe* sp. | Mycelium powdery, colony colour: light grey from above and white from reverse, fast-growing. | 124 | 1 | Lf | Vd / Sa | LGMF1532 | MG976356 | Figure S11 |
| *Diaporthe* sp. | Aerial mycelium, colony colour: white from above and cream to light yellow from reverse, fast-growing. | 11 | 15 | Lf / Pt | Vd / Sa | LGMF1538 | MG976357 | Figure S11 |
| *Diaporthe* sp. | Aerial mycelium, colony colour: white from above and white with black from reverse, fast-growing. | 12 | 31 | Lf / Pt | Vd / Sa | LGMF1539 | MG976358 | Figure S11 |
| *Diaporthe* sp. | Aerial mycelium, colony colour: grey to grey greenish, from above and dark from reverse, fast-growing. | 13 | 19 | Lf / Pt | Vd / Sa | LGMF1540 | MG976359 | Figure S11 |
| *Diaporthe* *schini* | Aerial mycelium, colony colour: white from above and white to cream from reverse, fast-growing. | 17 | 112 | Lf / Pt | Vd / Sa | LGMF1541 | MG976363 | Figure S11 |
| *Diaporthe* sp. | Aerial mycelium, colony colour: white from above and cream to brown from reverse, fast-growing. | 29 | 1 | Lf | Vd | LGMF1548 | MG976364 | Figure S11 |
| *Diaporthe* sp.2 | Aerial mycelium, colony colour: white to brown from above and dark brown from reverse, fast-growing. | 30 | 1 | Lf | Vd / Sa | **LGMF1549** | MG976368 | Figure S11 |
| *Diaporthe* sp. | Aerial mycelium, colony colour: cream white from above and white to reddish from reverse, fast-growing. | 39 | 2 | Pt | Vd / Sa | LGMF1554 | MG976372 | Figure S11 |
| *Diaporthe* sp. | Aerial mycelium, colony colour: cream white from above and light grey from reverse, fast-growing. | 49 | 5 | Lf / Pt | Vd / Sa | LGMF1561 | MG976374 | Figure S11 |
| *Diaporthe* sp. | Aerial mycelium, colony colour: white to reddish from above and white, fast-growing. | 52 | 5 | Lf / Pt | Vd / Sa | LGMF1563 | MG976375 | Figure S11 |
| *Diaporthe* cf*. heveae* 1 | Aerial mycelium, colony colour: cream white from above and cream from reverse, fast-growing. | 56 | 6 | Lf / Pt | Vd / Sa | LGMF1565 | MG976377 | Figure S11 |
| *Diaporthe* sp. | Aerial mycelium, colony colour: white from above and dark grey from reverse, fast-growing. | 58 | 6 | Lf / Pt | Vd / Sa | LGMF1567 | MG976378 | Figure S11 |
| *Diaporthe* cf*. heveae* 1 | Aerial mycelium, colony colour: white to light grey from above and dark grey from reverse, fast-growing. | 60 | 2 | Pt | Vd / Sa | LGMF1569 | MG976382 | Figure S11 |
| *Diaporthe* sp. | Aerial mycelium, colony colour: white to light grey from above and dark grey to black from reverse, fast-growing. | 65 | 2 | Pt | Vd / Sa | LGMF1573 | MG976385 | Figure S11 |
| *Diaporthe* sp. | Aerial mycelium, colony colour: white to yellow from above and white from reverse, fast-growing. | 69 | 4 | Pt | Vd / Sa | LGMF1576 | MG976391 | Figure S11 |
| *Diaporthe* sp. | Aerial mycelium, colony colour: dark brown with white patches from above and cream to dark brown from reverse, fast-growing. | 80 | 1 | Pt | Vd | LGMF1583 | MG976347 | Figure S11 |
| *Diaporthe* sp. | Aerial mycelium, colony colour: cream to brown from above and cream to brown from reverse, fast-growing. | 83 | 1 | Lf | Vd / Sa | LGMF1585 | MG976399 | Figure S11 |
| *Diaporthe* cf*. heveae* 1 | Aerial mycelium, colony colour: white from above and white to grey from reverse, fast-growing. | 91 | 2 | Pt | Vd / Sa | LGMF1593 | MG976400 | Figure S11 |
| *Diaporthe* cf*. heveae* 1 | Aerial mycelium, colony colour: light grey from above and white from reverse, fast-growing. | 92 | 1 | Lf | Vd / Sa | LGMF1594 | MG976404 | Figure S11 |
| *Diaporthe* sp. | Aerial mycelium, colony colour: light pink from above and white from reverse, fast-growing. | 101 | 1 | Lf | Vd / Sa | LGMF1601 | MG976405 | Figure S11 |
| *Diaporthe* sp. | Aerial mycelium, colony colour: white to orange from above and white from reverse, fast-growing. | 102 | 1 | Lf | Vd / Sa | LGMF1602 | MG976409 | Figure S11 |
| *Diaporthe* sp. | Mycelium cottony, colony colour: white from above and white from reverse, fast-growing. | 113 | 3 | Lf | Vd / Sa | LGMF1606 | LGMF1612 | Figure S11 |
| *Diaporthe* sp. | Mycelium cottony, colony colour: black from above and grey to back from reverse, fast-growing. | 4 | 16 | Lf / Pt | Vd / Sa | LGMF1612 | LGMF1614 | Figure S11 |
| *Diaporthe* sp. | Mycelium cottony, colony colour: white from above and white to brown from reverse, fast-growing. | 10 | 11 | Lf / Pt | Vd / Sa | LGMF1614 | LGMF1615 | Figure S11 |
| *Diaporthe* sp. | Mycelium cottony, colony colour: white to black from above and brown from reverse, fast-growing. | 14 | 14 | Lf / Pt | Vd / Sa | LGMF1615 | MG976419 | Figure S11 |
| *Diaporthe* sp.2 | Aerial mycelium, colony colour: white from above and cream to brown from reverse, fast-growing. | 15 | 33 | Lf / Pt | Vd / Sa | **LGMF1616** | MG976421 | Figure S11 |
| *Diaporthe* sp.2 | Mycelium cottony, colony colour: white from above and white with black from reverse, fast-growing. | 19 | 73 | Lf / Pt | Vd / Sa | **LGMF1618** | MG976423 | Figure S11 |
| *Diaporthe* sp.1 | Mycelium cottony, colony colour: white to grey from above and grey yellowish from reverse, fast-growing. | 21 | 31 | Lf / Pt | Vd / Sa | **LGMF1620** | MG976429 | Figure S11 |
| *Diaporthe* sp. | Pulvinate mycelium, colony colour: white to yellow from above and reverse, fast-growing. | 37 | 3 | Lf / Pt | Vd / Sa | LGMF1626 | MG976432 | Figure S11 |
| *Diaporthe* sp. | Mycelium cottony, colony colour: white to grey from above and grey greyish white from reverse, fast-growing. | 46 | 4 | Lf / Pt | Vd / Sa | LGMF1629 | MG976433 | Figure S11 |
| *Diaporthe* cf*. heveae* 1 | Mycelium cottony, colony colour: white to cream from above and cream to brown from reverse, fast-growing. | 54 | 2 | Pt | Sa | LGMF1631 | MG976434 | Figure S11 |
| *Diaporthe* *ocoteae* | Pulvinate mycelium, colony colour: white to yellow from above and dark brown reverse, fast-growing. | 55 | 4 | Lf / Pt | Vd / Sa | LGMF1632 | MG976435 | Figure S11 |
| *Diaporthe* cf*. heveae* 1 | Mycelium cottony, colony colour: white to grey from above and cream to brown from reverse, fast-growing. | 63 | 5 | Pt | Vd / Sa | LGMF1633 | MG976437 | Figure S11 |
| *Diaporthe* cf*. heveae* 1 | Mycelium cottony, colony colour: white to grey from above and grey to brown from reverse, fast-growing. | 70 | 2 | Lf / Pt | Vd / Sa | LGMF1635 | MG976441 | Figure S11 |
| *Diaporthe* sp.1 | Mycelium cottony, colony colour: white from above and brown to reddish from reverse, fast-growing. | 81 | 51 | Lf / Pt | Vd / Sa | **LGMF1639** | MG976346 | Figure S11 |
| *Diaporthe schini* | Aerial mycelium, colony colour: cream from above and cream to brown from reverse, fast-growing. | 120 | 1 | Lf | Vd / Sa | LGMF1525 | MG976361 | Figure S11 |
| Diatrypaceae sp. | Aerial mycelium, colony colour: light pink from above and black from reverse, fast-growing. | 24 | 3 | Lf / Pt | Vd / Sa | LGMF1544 | MG976420 | Figure S15 |
| *Didymella* sp. | Aerial mycelium, colony colour: white from above and brown from reverse, fast-growing. | 16 | 32 | Lf / Pt | Vd / Sa | LGMF1617 | MG976427 | Figure S12 |
| *Didymella* sp. | Aerial mycelium, colony colour: dark grey from above and black from reverse, fast-growing. | 32 | 4 | Lf / Pt | Vd / Sa | LGMF1624 | MG976390 | Figure S12 |
| *Efibula* sp. | Mycelium cottony, colony colour: white to grey from above and dark grey from reverse, fast-growing. | 78 | 1 | Pt | Vd | **LGMF1581** | MG976370 | Figure S13 |
| *Epicoccum* sp.1 | Mycelium cottony, colony colour: white to reddish from above and grey from reverse, fast-growing. | 43 | 3 | Lf / Pt | Vd / Sa | **LGMF1557** | MG976379 | Figure S14 |
| *Epicoccum* sp.2 | Mycelium cottony, colony colour: white to orange from above and dark grey to yellow from reverse, fast-growing. | 61 | 5 | Lf / Pt | Vd / Sa | **LGMF1570** | MG976402 | Figure S14 |
| *Epicoccum* sp.1 | Mycelium cottony, colony colour: light pink to orange from above and dark red from reverse, fast-growing. | 98 | 8 | Lf | Vd / Sa | **LGMF1598** | MG976403 | Figure S14 |
| *Epicoccum* sp.2 | Mycelium cottony, colony colour: white to grey to from above and dark red with black patches from reverse, fast-growing. | 100 | 1 | Lf | Vd / Sa | **LGMF1600** | MG976431 | Figure S14 |
| *Epicoccum* sp. | Mycelium cottony, colony colour: light pink to grey from above and grey with red patches from reverse, fast-growing. | 45 | 4 | Lf | Vd / Sa | **LGMF1628** | MG976442 | Figure S14 |
| *Epicoccum* sp. | Mycelium cottony, colony colour: grey to yellow from above and red to orange from reverse, fast-growing. | 97 | 2 | Pt | Vd / Sa | **LGMF1641** | MG976365 | Figure S14 |
| Fungi | Aerial mycelium, colony colour: white to brown from above and white from reverse, low-growing. | 8 | 19 | Lf / Pt | Vd / Sa | LGMF1536 | - | - |
| Fungi | Aerial mycelium, white to brown from above and purple from reverse, low-growing. | 42 | 1 | Pt | Vd / Sa | LGMF1556 | - | - |
| Fungi | Aerial mycelium, colony colour: white and light rose, from above, and rose to violet, from reverse, low-growing | 79 | 1 | Pt | Vd / Sa | LGMF1582 | - | - |
| Fungi | Aerial mycelium colony colour: pink to white, from above, and grey, from reverse, low-growing. | 94 | 3 | Lf | Vd / Sa | LGMF1596 | - | - |
| Fungi | Aerial mycelium colony colour: white to purple, from above and cream, from reverse, low-growing. | 95 | 3 | Lf | Vd / Sa | LGMF1597 | - | - |
| Fungi | Aerial mycelium colony colour: white to cream, from above, and cream with black patches, from reverse, moderate-growing. | 99 | 1 | Pt | Vd / Sa | LGMF1599 | - | - |
| Fungi | Fluffy mycelium, colony colour: yellow from above and cream to reverse, moderate-growing. | 96 | 1 | Pt | Vd / Sa | LGMF1640 | - | - |
| *Fusarium* sp. | Mycelium cottony, colony colour: light pink from above and dark pink from reverse, fast-growing | 23 | 9 | Lf / Pt | Vd / Sa | LGMF1543 | - | Figure S16 |
| *Fusarium* sp.1 | Mycelium cottony, colony colour: white-creamy from above and brown from reverse, fast-growing. | 26 | 2 | Pt | Vd / Sa | **LGMF1546** | MH105813 | Figure S16 |
| *Fusarium* sp.2 | Mycelium cottony, colony colour: white to pink, from above and red, from reverse, fast-growing. | 27 | 1 | Lf | Vd / Sa | **LGMF1547** | MH105814 | Figure S16 |
| *Fusarium* sp.1 | Mycelium cottony, colony colour: white from above and red from reverse, fast-growing. | 38 | 1 | Pt | Vd / Sa | **LGMF1553** | MH105815 | Figure S16 |
| *Fusarium* sp.2 | Mycelium cottony, colony colour: light violet from above and dark violet from reverse, fast-growing. | 44 | 1 | Lf | Vd / Sa | **LGMF1558** | MH105816 | Figure S16 |
| *Fusarium* sp. | Mycelium cottony, colony colour: light pink from above and dark pink from reverse, fast-growing. | 88 | 3 | Lf / Pt | Vd / Sa | LGMF1590 | MH105817 | Figure S16 |
| *Fusarium* sp. | Mycelium cottony, colony colour: violet from above and cream from reverse, fast-growing. | 53 | 1 | Pt | Vd / Sa | LGMF1564 | MH144142 | Figure S16 |
| *Fusarium* sp. | Mycelium cottony, colony colour: white to red, from above and cream from reverse, fast-growing. | 57 | 2 | Lf / Pt | Vd / Sa | LGMF1566 | MG976376 | - |
| *Fusarium* sp. | Mycelium cottony, colony colour: red from above and cream with red patches from reverse, fast-growing. | 59 | 3 | Lf / Pt | Vd / Sa | LGMF1568 | MH144143 | - |
| *Fusarium* sp. | Mycelium cottony, colony colour: white from above and cream with red patches from reverse, fast-growing. | 85 | 3 | Lf | Vd / Sa | LGMF1587 | MG976394 | - |
| *Fusarium* sp. | Mycelium cottony, colony colour: white to orange, from above and cream with orange patches, from reverse, fast-growing. | 87 | 1 | Pt | Vd / Sa | LGMF1589 | MH144144 | - |
| *Fusarium* sp. | Mycelium cottony, dark violet from above and dark violet from reverse, fast-growing. | 48 | 1 | Pt | Vd / Sa | LGMF1560 | MG976396 | - |
| *Fusarium* sp. | Mycelium cottony, colony colour: dark red from above and dark red from reverse, fast-growing. | 51 | 3 | Pt | Vd / Sa | LGMF1630 | MH144145 | - |
| *Hypoxylon* sp.1 | Mycelium cottony, colony colour: white, from above and white with green dark, from reverse, fast-growing. | 6 | 23 | Lf / Pt | Vd / Sa | LGMF1613 | MG976416 | Figure S17 |
| *Lasiodiplodia* sp. | Mycelium powdery, colony colour: light rose, from above and white with black patches, from reverse, moderate-growing. | 123 | 1 | Lf | Vd / Sa | LGMF1609 | MG976412 | Figure S18 |
| *Lasiodiplodia*sp. | Mycelium powdery, colony colour: brown, from above and from reverse, moderate-growing | 5 | 22 | Lf / Pt | Vd / Sa | LGMF1534 | MG976353 | Figure S18 |
| *Lasiodiplodia*sp. | Mycelium powdery colony colour: cream, from above and dark brown, from reverse, moderate-growing. | 40 | 2 | Lf / Pt | Vd / Sa | LGMF1555 | MG976369 | Figure S18 |
| *Neofusicoccum* *brasiliense* | Aerial mycelium, colony colour: white to brown, from above and white with black, from reverse, fast-growing. | 7 | 50 | Lf / Pt | Vd / Sa | LGMF1535 | MG976354 | Figure S19 |
| *Neofusicoccum* *brasiliense* | Aerial mycelium, colony colour: white, from above and black, from reverse, fast-growing. | 67 | 6 | Lf / Pt | Vd / Sa | LGMF1634 | MG976436 | Figure S19 |
| *Neopestalotiopsis* sp. | Aerial mycelium, colony colour: dark, from above and from reverse, fast-growing | 50 | 3 | Lf / Pt | Vd | LGMF1562 | MG976373 | Figure S20 |
| *Nigrospora* *hainanensis* | Aerial mycelium colony colour: white with black patches, from above and white, from reverse, moderate-growing. | 89 | 2 | Lf | Vd / Sa | LGMF1591 | MG976397 | Figure S21 |
| *Nigrospora* *hainanensis* | Aerial mycelium colony colour: black, from above and grey, from reverse, moderate-growing | 28 | 2 | Lf | Vd / Sa | LGMF1622 | MG976425 | Figure S21 |
| *Paraphaeosphaeria* sp. | Dense mycelium, colony colour: green to greyish, from above and dark, from reverse, low-growing. | 110 | 1 | Pt | Vd / Sa | LGMF1515 | MG976336 | Figure S22 |
| *Paraphaeosphaeria* sp. | Powdery mycelium, colony colour: dark green, from above and dark green, from reverse, moderate-growing. | 111 | 1 | Lf | Vd | LGMF1516 | MG976337 | Figure S22 |
| *Pestalotiopsis* sp. | Aerial mycelium colony colour: grayish to white, from above, and dark from reverse, moderate-growing. | 34 | 4 | Pt | Vd / Sa | LGMF1551 | MG976366 | Figure S23 |
| *Pestalotiopsis* sp. | Aerial mycelium colony colour: grayish to white, from above and grey, from reverse, fast-growing. | 35 | 3 | Lf / Pt | Vd / Sa | LGMF1552 | MG976367 | Figure S23 |
| *Pestalotiopsis* sp. | Aerial mycelium colony colour: grayish to white, from above and dark grey with black patches, from reverse, fast-growing. | 75 | 1 | Lf | Vd / Sa | LGMF1637 | MG976439 | Figure S23 |
| *Pestalotiopsis* sp. | Aerial mycelium colony colour: grayish to white, from above and white with black patches, from reverse, fast-growing. | 77 | 1 | Lf | Vd / Sa | LGMF1638 | MG976440 | Figure S23 |
| *Pestalotiopsis* sp.1 | Dense mycelium colony colour: greenish to grey, from above and black, from reverse, moderate-growing. | 115 | 3 | Lf | Vd / Sa | LGMF1519 | MG976340 | Figure S23 |
| *Phaeophleospora* sp.1 | Mycelium cottony, colony colour: white to redish, from above and red, from reverse, fast-growing. | 82 | 2 | Lf | Vd / Sa | **LGMF1584** | MG976392 | Figure S24 |
| *Phaeophleospora* sp.2 | Mycelium cottony, colony colour: white, from above and white with bright red, from reverse fast-growing. | 109 | 2 | Lf | Vd / Sa | **LGMF1513** | MG976335 | Figure S24 |
| *Phaeophleospora* sp.2 | Mycelium powdery colony colour: dark with white patches, from above and dark, from reverse, fast-growing. | 118 | 1 | Pt | Vd / Sa | **LGMF1524** | MG976345 | Figure S24 |
| *Phyllosticta* sp.1 | Mycelium powdery, colony colour: light grey, from above and dark from reverse, moderate-growing. | 107 | 1 | Lf | Vd / Sa | **LGMF1511** | MG976333 | Figure S25 |
| *Phyllosticta* sp.1 | Mycelium powdery, colony colour: grey to green, from above and dark, from reverse, moderate-growing. | 108 | 1 | Lf | Vd / Sa | **LGMF1512** | MG976334 | Figure S25 |
| *Phyllosticta* sp. | Mycelium powdery, colony colour: black, from above and dark, from reverse, moderate-growing. | 116 | 123 | Lf / Pt | Vd / Sa | LGMF1521 | MG976342 | Figure S25 |
| Pleomassariaceae sp. | Mycelium powdery, colony colour: white to grey, from above and black with yellow, from reverse, fast-growing. | 105 | 1 | Lf | Vd / Sa | LGMF1509 | MG976331 | - |
| *Pseudofusicoccum* sp.1 | Mycelium cottony, colony colour: light grey, from above and white with grey, from reverse, fast-growing. | 3 | 22 | Lf / Pt | Vd / Sa | **LGMF1611** | MG976414 | Figure S25 |
| *Pseudofusicoccum stromaticcum* | Aerial mycelium colony colour: white, from above and white with yellow patches, from reverse, fast-growing. | 119 | 3 | Lf | Vd / Sa | LGMF1608 | MG976411 | Figure S26 |
| *Roussoella* sp. | Powdery mycelium, colony colour: white with green, from above and dark, from reverse, moderate-growing. | 114 | 2 | Lf | Vd | **LGMF1518** | MG976339 | Figure S27 |
| Xylariaceae sp.3 | Aerial mycelium white, from above and white to grey, from reverse, fast-growing | 18 | 44 | Lf / Pt | Vd / Sa | LGMF1542* | MG976360 | Figure 2 |
| Xylariaceae sp.4 | Aerial mycelium colony colour: light rose, from above and white to from reverse, fast-growing. | 25 | 3 | Lf / Pt | Vd / Sa | LGMF1545* | MG976362 | Figure 2 |
| Xylariaceae sp.4 | Aerial mycelium colony colour: white to grey, from above and, from reverse, fast-growing. | 64 | 2 | Pt | Vd / Sa | LGMF1572* | MG976381 | Figure 2 |
| Xylariaceae sp.4 | Aerial mycelium colony colour: grey, from above and dark, from reverse, fast-growing. | 66 | 6 | Lf / Pt | Vd / Sa | LGMF1574* | MG976383 | Figure 2 |
| Xylariaceae sp.5 | Aerial mycelium colony colour: white, from above and yellowish, from reverse, fast-growing. | 9 | 29 | Lf / Pt | Vd / Sa | LGMF1537* | MG976355 | Figure 2 |

Table S2-Indicator Species Values table for morphotypes. Highlighted values indicate

significant representatives of a group. *(Continue)*

| **Morphotype** | ***S. adstringens* Leaf** | ***S. adstringens* Petiole** | ***V. divergens* Leaf** | ***V. divergens* Petiole** | **P** |
| --- | --- | --- | --- | --- | --- |
| **1** | 0.08 | 0.06 | 0.07 | 0.22 | 0.20 |
| **2** | 0.02 | 0.08 | 0.07 | 0.11 | 0.82 |
| **3** | 0.02 | 0.04 | 0.06 | 0.11 | 0.65 |
| **4** | 0.00 | 0.04 | 0.22 | 0.01 | 0.04 |
| **5** | 0.02 | 0.05 | 0.07 | 0.10 | 0.79 |
| **6** | 0.01 | 0.10 | 0.01 | 0.23 | 0.05 |
| **7** | 0.15 | 0.09 | 0.03 | 0.25 | 0.13 |
| **8** | 0.01 | 0.10 | 0.02 | 0.17 | 0.20 |
| **9** | 0.20 | 0.00 | 0.06 | 0.19 | 0.24 |
| **10** | 0.06 | 0.09 | 0.00 | 0.04 | 0.51 |
| **11** | 0.05 | 0.02 | 0.05 | 0.07 | 0.92 |
| **12** | 0.03 | 0.01 | 0.11 | 0.19 | 0.19 |
| **13** | 0.11 | 0.00 | 0.04 | 0.14 | 0.31 |
| **14** | 0.07 | 0.02 | 0.15 | 0.01 | 0.25 |
| **15** | 0.18 | 0.04 | 0.03 | 0.16 | 0.37 |
| **16** | 0.06 | 0.01 | 0.08 | 0.32 | 0.02 |
| **17** | 0.16 | 0.05 | 0.12 | 0.35 | 0.03 |
| **18** | 0.18 | 0.05 | 0.07 | 0.20 | 0.43 |
| **19** | 0.19 | 0.09 | 0.12 | 0.19 | 0.77 |
| **20** | 0.09 | 0.00 | 0.05 | 0.08 | 0.81 |
| **21** | 0.06 | 0.05 | 0.07 | 0.20 | 0.21 |
| **22** | 0.00 | 0.05 | 0.01 | 0.05 | 0.72 |
| **23** | 0.00 | 0.03 | 0.02 | 0.15 | 0.13 |
| **24** | 0.00 | 0.03 | 0.01 | 0.02 | 1.00 |
| **25** | 0.00 | 0.00 | 0.06 | 0.02 | 0.45 |
| **26** | 0.00 | 0.00 | 0.00 | 0.06 | 0.70 |
| **27** | 0.00 | 0.00 | 0.05 | 0.00 | 1.00 |
| **28** | 0.17 | 0.00 | 0.00 | 0.00 | 0.03 |
| **29** | 0.00 | 0.00 | 0.05 | 0.00 | 1.00 |
| **30** | 0.00 | 0.00 | 0.05 | 0.00 | 1.00 |
| **31** | 0.00 | 0.00 | 0.00 | 0.11 | 0.17 |
| **32** | 0.03 | 0.00 | 0.04 | 0.01 | 0.86 |
| **33** | 0.00 | 0.00 | 0.05 | 0.06 | 0.53 |
| **34** | 0.00 | 0.00 | 0.00 | 0.11 | 0.18 |
| **35** | 0.00 | 0.00 | 0.02 | 0.04 | 0.67 |
| **36** | 0.00 | 0.00 | 0.09 | 0.05 | 0.33 |
| **37** | 0.00 | 0.01 | 0.00 | 0.09 | 0.25 |
| **38** | 0.00 | 0.00 | 0.00 | 0.06 | 0.71 |
| **39** | 0.00 | 0.00 | 0.00 | 0.11 | 0.16 |
| **40** | 0.00 | 0.00 | 0.02 | 0.03 | 1.00 |
| **41** | 0.00 | 0.00 | 0.00 | 0.06 | 0.69 |
| **42** | 0.00 | 0.00 | 0.00 | 0.06 | 0.68 |
| **43** | 0.00 | 0.00 | 0.03 | 0.02 | 0.94 |
| **44** | 0.00 | 0.00 | 0.05 | 0.00 | 1.00 |
| **45** | 0.00 | 0.00 | 0.05 | 0.00 | 1.00 |
| **46** | 0.00 | 0.04 | 0.02 | 0.00 | 0.69 |
| **47** | 0.02 | 0.01 | 0.03 | 0.03 | 1.00 |
| **48** | 0.00 | 0.00 | 0.02 | 0.04 | 0.66 |
| **49** | 0.00 | 0.00 | 0.02 | 0.04 | 0.67 |
| **50** | 0.00 | 0.00 | 0.02 | 0.08 | 0.35 |
| **51** | 0.03 | 0.00 | 0.01 | 0.03 | 0.86 |
| **52** | 0.00 | 0.02 | 0.01 | 0.07 | 0.45 |
| **53** | 0.00 | 0.00 | 0.00 | 0.06 | 0.70 |
| **54** | 0.00 | 0.13 | 0.00 | 0.00 | 0.09 |
| **55** | 0.00 | 0.02 | 0.05 | 0.01 | 0.78 |
| **56** | 0.00 | 0.00 | 0.02 | 0.11 | 0.16 |
| **57** | 0.00 | 0.00 | 0.02 | 0.03 | 1.00 |
| **58** | 0.00 | 0.00 | 0.05 | 0.06 | 0.57 |
| **59** | 0.00 | 0.00 | 0.06 | 0.02 | 0.47 |
| **60** | 0.00 | 0.04 | 0.00 | 0.03 | 0.84 |
| **61** | 0.02 | 0.00 | 0.02 | 0.04 | 0.80 |
| **62** | 0.00 | 0.00 | 0.07 | 0.02 | 0.41 |
| **63** | 0.00 | 0.13 | 0.00 | 0.04 | 0.13 |
| **64** | 0.00 | 0.00 | 0.00 | 0.11 | 0.14 |
| **65** | 0.00 | 0.00 | 0.00 | 0.11 | 0.18 |
| **66** | 0.00 | 0.01 | 0.02 | 0.08 | 0.40 |
| **67** | 0.00 | 0.00 | 0.00 | 0.28 | 0.01 |
| **68** | 0.00 | 0.00 | 0.06 | 0.02 | 0.46 |
| **69** | 0.00 | 0.00 | 0.05 | 0.03 | 0.70 |
| **70** | 0.00 | 0.00 | 0.02 | 0.03 | 1.00 |
| **71** | 0.00 | 0.00 | 0.00 | 0.06 | 0.69 |
| **72** | 0.13 | 0.00 | 0.01 | 0.00 | 0.11 |
| **73** | 0.00 | 0.00 | 0.04 | 0.02 | 0.87 |
| **74** | 0.08 | 0.00 | 0.00 | 0.00 | 0.20 |
| **75** | 0.08 | 0.00 | 0.00 | 0.00 | 0.18 |
| **76** | 0.00 | 0.00 | 0.00 | 0.06 | 0.68 |
| **77** | 0.08 | 0.00 | 0.00 | 0.00 | 0.19 |
| **78** | 0.00 | 0.00 | 0.00 | 0.06 | 0.70 |
| **79** | 0.00 | 0.00 | 0.00 | 0.06 | 0.67 |
| **80** | 0.00 | 0.00 | 0.00 | 0.06 | 0.73 |
| **81** | 0.07 | 0.02 | 0.21 | 0.16 | 0.26 |
| **82** | 0.00 | 0.00 | 0.05 | 0.00 | 1.00 |
| **83** | 0.00 | 0.00 | 0.05 | 0.00 | 1.00 |
| **84** | 0.00 | 0.04 | 0.02 | 0.00 | 0.71 |
| **85** | 0.00 | 0.00 | 0.15 | 0.00 | 0.06 |
| **86** | 0.00 | 0.00 | 0.06 | 0.02 | 0.49 |
| **87** | 0.00 | 0.00 | 0.00 | 0.06 | 0.69 |
| **88** | 0.00 | 0.00 | 0.00 | 0.06 | 0.70 |
| **89** | 0.00 | 0.00 | 0.05 | 0.00 | 1.00 |
| **90** | 0.00 | 0.00 | 0.05 | 0.00 | 1.00 |
| **91** | 0.00 | 0.04 | 0.00 | 0.03 | 0.81 |
| **92** | 0.00 | 0.00 | 0.05 | 0.00 | 1.00 |
| **93** | 0.00 | 0.00 | 0.05 | 0.00 | 1.00 |
| **94** | 0.00 | 0.03 | 0.06 | 0.00 | 0.59 |
| **95** | 0.00 | 0.00 | 0.05 | 0.00 | 1.00 |
| **96** | 0.00 | 0.07 | 0.00 | 0.00 | 0.42 |
| **97** | 0.00 | 0.04 | 0.00 | 0.03 | 0.82 |
| **98** | 0.00 | 0.14 | 0.01 | 0.01 | 0.11 |
| **99** | 0.00 | 0.00 | 0.00 | 0.06 | 0.73 |
| **100** | 0.00 | 0.00 | 0.05 | 0.00 | 1.00 |
| **101** | 0.00 | 0.00 | 0.05 | 0.00 | 1.00 |
| **102** | 0.00 | 0.00 | 0.05 | 0.00 | 1.00 |
| **103** | 0.13 | 0.03 | 0.41 | 0.07 | 0.01 |

| **Table S3**-Permanova results for morphotypes. | | | | | | |
| --- | --- | --- | --- | --- | --- | --- |
|  | **DF** | **Sum of Sq.** | **Mean of Sq.** | **F** | **R2** | **p** |
| **Plant** | 1 | 0.44 | 0.44 | 1.38 | 0.02 | 0.12 |
| **Tissues** | 1 | 0.61 | 0.61 | 1.89 | 0.03 | 0.02 |

Note: Highlighted values indicate significant differences inside a group. Number of permutations: 1000. DF=Degrees of freedom; Sq=squares; p= probability value.

Table S4-ANOSIMs results per morphotype comparing plants and tissues.

| **Petiole x Leaf (Vd and Sa)** | **Petiole x Leaf (Vd only)** |
| --- | --- |
| Dissimilarity: bray | Dissimilarity: bray |
| ANOSIM statistic R: 0.04677 | ANOSIM statistic R: 0.1111 |
| Significance: **0.014** | Significance: **0.002** |
| Permutation: free | Permutation: free |
| Number of permutations: 999 | Number of permutations: 999 |
| Upper quantiles of permutations (null model): | Upper quantiles of permutations (null model): |
| 90% 95% 97.5% 99% | 90% 95% 97.5% 99% |
| 0.0225 0.0302 0.0401 0.0518 | 0.0373 0.0525 0.0623 0.0766 |
| Dissimilarity ranks between and within classes: | Dissimilarity ranks between and within classes: |
| 0% 25% 50% 75% 100% N | 0% 25% 50% 75% 100% N |
| Between 1 550.5 1081.50 1808.5 1808.5 1056 | Between 2 196.000 394.25 540.25 635 360 |
| Leaf 5 433.0 929.50 1490.0 1808.5 496 | Leaf 1 184.375 348.00 635.00 635 190 |
| Petiole 5 527.5 1073.75 1808.5 1808.5 528 | Petiole 5 137.000 275.50 425.50 635 153 |

Note: Highlighted values indicate significant differences between morphogroups. Vd for *V. divergens* and Sa for *S. adstringens.*

| **Table S5-**Screening of antimicrobial activity of the endophytic fungi against phytopathogens. *(Continue)*   \| **Identification** \| **Strain** \| **Inhibition Rate (%)** \| \| \| \| --- \| --- \| --- \| --- \| --- \| \|  \|  \| *P. citricarpa* \| *C. abscissum* \| *F. verticillioides* \| \| *Curvularia* sp. \| LGMF1533 \| 20 \| 10 \| 8 \| \| *Diaporthe*  sp. \| LGMF1610 \| 45 \| 10 \| 5 \| \| *Pseudofusicoccum* sp. \| LGMF1611 \| 35 \| 39 \| 7 \| \| *Diaporthe* sp. \| LGMF1612 \| 33 \| 15 \| 9 \| \| *Lasiodiplodia* sp. \| LGMF1534 \| 35 \| 46 \| 0 \| \| *Hypoxylon* sp. \| LGMF1613 \| 50 \| 24 \| 10 \| \| *Neofusicoccum* *brasiliense* \| LGMF1535 \| 66 \| 47 \| 15 \| \| Xylariaceae sp. 5 \| LGMF1537 \| 47 \| 35 \| 10 \| \| *Diaporthe* sp. \| LGMF1614 \| 70 \| 36 \| 8 \| \| *Diaporthe* sp. \| LGMF1538 \| 44 \| 25 \| 8 \| \| *Diaporthe* sp. \| LGMF1539 \| 48 \| 35 \| 10 \| \| *Diaporthe* sp. \| LGMF1540 \| 51 \| 35 \| 4 \| \| *Diaporthe* sp. \| LGMF1615 \| 48 \| 30 \| 6 \| \| *Diaporthe* sp. \| LGMF1616 \| 47 \| 40 \| 9 \| \| *Didymella* sp. \| LGMF1617 \| 33 \| 30 \| 10 \| \| *Diaporthe* *schini* \| LGMF1541 \| 45 \| 25 \| 8 \| \| Xylariaceae sp.3 \| LGMF1542 \| 49 \| 30 \| 10 \| \| *Diaporthe* sp. \| LGMF1618 \| 37 \| 35 \| 8 \| \| *Corynespora* *cambrensis* \| LGMF1619 \| 38 \| 22 \| 7 \| \| *Diaporthe* sp. \| LGMF1620 \| 41 \| 30 \| 9 \| \| *Colletotrichum* sp. \| LGMF1621 \| 49 \| 22 \| 7 \| \| *Fusarium* sp. \| LGMF1543 \| 50 \| 38 \| 15 \| \| Diatrypaceae sp. \| LGMF1544 \| 36 \| 13 \| 8 \| \| Xylariaceae sp.4 \| LGMF1545 \| 48 \| 39 \| 10 \| \| *Fusarium* sp. \| LGMF1546 \| 51 \| 44 \| 10 \| \| *Fusarium* sp. \| LGMF1547 \| 49 \| 38 \| 12 \| \| *Nigrospora* sp. \| LGMF1622 \| 49 \| 31 \| 8 \| \| *Diaporthe* sp. \| LGMF1548 \| 46 \| 30 \| 10 \| \| *Diaporthe* sp. \| LGMF1549 \| 35 \| 30 \| 9 \| \| *Daldinia* sp. \| LGMF1623 \| 33 \| 14 \| 9 \| \| *Didymella* sp. \| LGMF1624 \| 54 \| 33 \| 7 \| \| *Alternaria* *alternata* \| LGMF1550 \| 38 \| 22 \| 5 \| \| *Pestalotiopsis* sp. \| LGMF1551 \| 36 \| 16 \| 8 \| \| *Pestalotiopsis* sp. \| LGMF1552 \| 51 \| 22 \| 7 \| \| *Colletotrichum* *siamense* \| LGMF1625 \| 48 \| 34 \| 3 \| \| *Diaporthe* sp. \| LGMF1626 \| 19 \| 14 \| 6 \| \| *Fusarium* sp. \| LGMF1553 \| 49 \| 38 \| 9 \| \| *Diaporthe* sp. \| LGMF1554 \| 47 \| 41 \| 8 \| \| *Lasiodiplodia* sp. \| LGMF1555 \| 20 \| 13 \| 7 \| \| *Bjerkandera* sp. \| LGMF1627 \| 33 \| 18 \| 0 \| \| *Epicoccum* sp. \| LGMF1557 \| 39 \| 36 \| 9 \| \| *Fusarium* sp. \| LGMF1558 \| 49 \| 22 \| 7 \| \| *Epicoccum* sp. \| LGMF1628 \| 44 \| 39 \| 9 \| \| *Diaporthe* sp. \| LGMF1629 \| 47 \| 38 \| 10 \| \| *Colletotrichum gloeosporioides* \| LGMF1559 \| 50 \| 41 \| 8 \| \| *Fusarium* sp. \| LGMF1560 \| 50 \| 42 \| 8 \| \| *Diaporthe* sp. \| LGMF1561 \| 49 \| 37 \| 9 \| \| *Neopestalotiopsis* sp. \| LGMF1562 \| 25 \| 50 \| 7 \| \| *Fusarium* sp. \| LGMF1630 \| 75 \| 66 \| 7 \| \| *Diaporthe* sp. \| LGMF1563 \| 45 \| 36 \| 8 \| \| *Fusarium* sp. \| LGMF1564 \| 51 \| 36 \| 9 \| \| *Diaporthe* cf*. heveae* \| LGMF1631 \| 80 \| 64 \| 22 \| \| *Diaporthe* sp. \| LGMF1632 \| 33 \| 25 \| 7 \| \| *Diaporthe* cf*. heveae* \| LGMF1565 \| 25 \| 14 \| 7 \| \| *Fusarium* sp. \| LGMF1566 \| 50 \| 38 \| 9 \| \| *Diaporthe* sp. \| LGMF1567 \| 39 \| 23 \| 7 \| \| *Fusarium* sp. \| LGMF1568 \| 47 \| 31 \| 9 \| \| *Diaporthe* cf*. heveae* \| LGMF1569 \| 37 \| 29 \| 10 \| \| *Epicoccum* sp. \| LGMF1570 \| 38 \| 29 \| 10 \| \| *Colletotrichum* sp. \| LGMF1571 \| 48 \| 33 \| 15 \| \| *Diaporthe* cf*. heveae* \| LGMF1633 \| 33 \| 19 \| 12 \| \| Xylariaceae sp.4 \| LGMF1572 \| 36 \| 24 \| 12 \| \| *Diaporthe* sp. \| LGMF1573 \| 38 \| 28 \| 9 \| \| Xylariaceae sp.4 \| LGMF1574 \| 36 \| 23 \| 17 \| \| *Neofusicoccum* *brasiliense* \| LGMF1634 \| 45 \| 34 \| 9 \| \| *Conidiochaeta* *nepalica* \| LGMF1575 \| 33 \| 19 \| 0 \| \| *Diaporthe* sp. \| LGMF1576 \| 39 \| 24 \| 14 \| \| *Diaporthe* cf*. heveae* \| LGMF1635 \| 44 \| 32 \| 6 \| \| *Colletotrichum* sp. \| LGMF1577 \| 47 \| 33 \| 6 \| \| *Bjerkandera* sp. \| LGMF1578 \| 38 \| 19 \| 0 \| \| *Bjerkandera* sp. \| LGMF1579 \| 38 \| 21 \| 0 \| \| *Colletotrichum* *siamense* \| LGMF1636 \| 44 \| 34 \| 0 \| \| *Pestalotiopsis* sp. \| LGMF1637 \| 45 \| 33 \| 0 \| \| *Colletotrichum* *gloeosporioides* \| LGMF1580 \| 48 \| 35 \| 0 \| \| *Pestalotiopsis* sp. \| LGMF1638 \| 36 \| 22 \| 8 \| \| *Efibula* sp. \| LGMF1581 \| 33 \| 18 \| 9 \| \| *Diaporthe* sp. \| LGMF1583 \| 38 \| 26 \| 10 \| \| *Diaporthe* sp. \| LGMF1639 \| 41 \| 26 \| 9 \| \| *Phaeophleospora* sp. \| LGMF1584 \| 40 \| 28 \| 14 \| \| *Diaporthe* sp. \| LGMF1585 \| 39 \| 27 \| 10 \| \| *Colletotrichum* g*loeosporioides* \| LGMF1586 \| 35 \| 30 \| 6 \| \| *Fusarium* sp. \| LGMF1587 \| 48 \| 35 \| 9 \| \| *Colletotrichum*  *gloeosporioides* \| LGMF1588 \| 44 \| 32 \| 6 \| \| *Fusarium* sp. \| LGMF1589 \| 55 \| 19 \| 9 \| \| *Fusarium* sp. \| LGMF1590 \| 46 \| 22 \| 9 \| \| *Nigrospora* *hainanensis* \| LGMF1591 \| 36 \| 33 \| 12 \| \| *Colletotrichum boninense* \| LGMF1592 \| 38 \| 45 \| 3 \| \| *Diaporthe* cf*. heveae* \| LGMF1593 \| 32 \| 23 \| 5 \| \| *Diaporthe* cf*. heveae* \| LGMF1594 \| 47 \| 27 \| 0 \| \| *Colletotrichum* *siamense* \| LGMF1595 \| 31 \| 22 \| 0 \| \| *Epicoccum* sp. \| LGMF1641 \| 78 \| 60 \| 15 \| \| *Epicoccum* sp. \| LGMF1598 \| 25 \| 37 \| 7 \| \| *Epicoccum* sp. \| LGMF1600 \| 31 \| 47 \| 6 \| \| *Diaporthe* sp. \| LGMF1601 \| 48 \| 44 \| 14 \| \| *Diaporthe* sp. \| LGMF1602 \| 50 \| 37 \| 12 \| \| *Diaporthe* sp. \| LGMF1508 \| 85 \| 31 \| 2 \| \| *Colletotrichum siamense* \| LGMF1604 \| 85 \| 62 \| 5 \| \| Pleomassariaceae sp. \| LGMF1509 \| 21 \| 29 \| 7 \| \| *Acrocalymma* sp. \| LGMF1605 \| 32 \| 29 \| 6 \| \| *Phyllosticta* sp. \| LGMF1511 \| 22 \| 19 \| 3 \| \| *Phyllosticta* sp. \| LGMF1512 \| 69 \| 30 \| 6 \| \| *Phaeophleospora* sp. \| LGMF1513 \| 35 \| 35 \| 7 \| \| *Paraphaeosphaeria s*p. \| LGMF1515 \| 76 \| 22 \| 7 \| \| *Paraphaeosphaeria* sp. \| LGMF1516 \| 26 \| 36 \| 7 \| \| *Diaporthe* sp. \| LGMF1517 \| 54 \| 40 \| 6,7 \| \| *Diaporthe* sp. \| LGMF1606 \| 60 \| 41 \| 6,3 \| \| *Roussoella* sp. \| LGMF1518 \| 18 \| 31 \| 8 \| \| *Pestalotiopsis* sp. \| LGMF1519 \| 20 \| 64 \| 13 \| \| *Phyllosticta* sp. \| LGMF1521 \| 24 \| 32 \| 7,5 \| \| *Colletotrichum boninense* \| LGMF1522 \| 24 \| 50 \| 7,5 \| \| *Phaeophleospora* sp. \| LGMF1524 \| 25 \| 61 \| 17 \| \| *Pseudofusicoccum* *stromaticcum* \| LGMF1608 \| 26 \| 31 \| 6 \| \| *Diaporthe*  *schini* \| LGMF1525 \| 10 \| 24 \| 6 \| \| *Diaporthe* sp. \| LGMF1530 \| 82 \| 67 \| 15 \| \| *Diaporthe* sp. \| LGMF1531 \| 43 \| 43 \| 17 \| \| *Lasiodiplodia* sp. \| LGMF1609 \| 26 \| 26 \| 9 \| \| *Diaporthe* sp. \| LGMF1532 \| 48 \| 42 \| 10 \| |
| --- | --- | --- | --- | --- | --- | --- | --- | --- | --- | --- | --- | --- | --- | --- | --- | --- | --- | --- | --- | --- | --- | --- | --- | --- | --- | --- | --- | --- | --- | --- | --- | --- | --- | --- | --- | --- | --- | --- | --- | --- | --- | --- | --- | --- | --- | --- | --- | --- | --- | --- | --- | --- | --- | --- | --- | --- | --- | --- | --- | --- | --- | --- | --- | --- | --- | --- | --- | --- | --- | --- | --- | --- | --- | --- | --- | --- | --- | --- | --- | --- | --- | --- | --- | --- | --- | --- | --- | --- | --- | --- | --- | --- | --- | --- | --- | --- | --- | --- | --- | --- | --- | --- | --- | --- | --- | --- | --- | --- | --- | --- | --- | --- | --- | --- | --- | --- | --- | --- | --- | --- | --- | --- | --- | --- | --- | --- | --- | --- | --- | --- | --- | --- | --- | --- | --- | --- | --- | --- | --- | --- | --- | --- | --- | --- | --- | --- | --- | --- | --- | --- | --- | --- | --- | --- | --- | --- | --- | --- | --- | --- | --- | --- | --- | --- | --- | --- | --- | --- | --- | --- | --- | --- | --- | --- | --- | --- | --- | --- | --- | --- | --- | --- | --- | --- | --- | --- | --- | --- | --- | --- | --- | --- | --- | --- | --- | --- | --- | --- | --- | --- | --- | --- | --- | --- | --- | --- | --- | --- | --- | --- | --- | --- | --- | --- | --- | --- | --- | --- | --- | --- | --- | --- | --- | --- | --- | --- | --- | --- | --- | --- | --- | --- | --- | --- | --- | --- | --- | --- | --- | --- | --- | --- | --- | --- | --- | --- | --- | --- | --- | --- | --- | --- | --- | --- | --- | --- | --- | --- | --- | --- | --- | --- | --- | --- | --- | --- | --- | --- | --- | --- | --- | --- | --- | --- | --- | --- | --- | --- | --- | --- | --- | --- | --- | --- | --- | --- | --- | --- | --- | --- | --- | --- | --- | --- | --- | --- | --- | --- | --- | --- | --- | --- | --- | --- | --- | --- | --- | --- | --- | --- | --- | --- | --- | --- | --- | --- | --- | --- | --- | --- | --- | --- | --- | --- | --- | --- | --- | --- | --- | --- | --- | --- | --- | --- | --- | --- | --- | --- | --- | --- | --- | --- | --- | --- | --- | --- | --- | --- | --- | --- | --- | --- | --- | --- | --- | --- | --- | --- | --- | --- | --- | --- | --- | --- | --- | --- | --- | --- | --- | --- | --- | --- | --- | --- | --- | --- | --- | --- | --- | --- | --- | --- | --- | --- | --- | --- | --- | --- | --- | --- | --- | --- | --- | --- | --- | --- | --- | --- | --- | --- | --- | --- | --- | --- | --- | --- | --- | --- | --- | --- | --- | --- | --- | --- | --- | --- | --- | --- | --- | --- | --- | --- | --- | --- | --- | --- | --- | --- | --- | --- | --- | --- | --- | --- | --- | --- | --- | --- | --- | --- | --- | --- | --- | --- | --- | --- | --- | --- | --- | --- | --- | --- | --- | --- | --- | --- | --- | --- | --- | --- | --- | --- | --- | --- | --- | --- | --- | --- | --- | --- | --- | --- | --- | --- | --- | --- | --- | --- | --- | --- | --- | --- | --- | --- | --- | --- | --- | --- | --- | --- | --- | --- | --- | --- | --- | --- | --- | --- | --- | --- | --- | --- | --- | --- | --- | --- | --- | --- | --- | --- | --- | --- | --- | --- | --- | --- | --- | --- | --- | --- | --- | --- | --- | --- | --- | --- | --- | --- | --- | --- | --- | --- | --- | --- | --- | --- | --- | --- | --- | --- | --- | --- | --- | --- | --- | --- | --- | --- | --- | --- | --- | --- | --- | --- | --- | --- | --- | --- | --- | --- | --- | --- | --- | --- | --- | --- | --- | --- | --- | --- | --- | --- | --- | --- | --- | --- | --- | --- | --- | --- | --- | --- | --- | --- | --- | --- | --- | --- | --- | --- | --- | --- | --- | --- | --- |

| **Table S6-**Screening of antimicrobial activity of the endophytic fungi against clinical pathogens.   \| Identification \| **Strain** \| **Spectrum of Action** \| \| --- \| --- \| --- \| \| \| *Curvularia* sp. \| LGMF1533 \| MSSA (+); MRSA (+); *P. aeruginosa* (-); *A. baumannii* (-); KPC (-) \| \| *Diaporthe* sp. \| LGMF1610 \| MSSA (+); MRSA (+);  *P. aeruginosa* (+); *A. baumannii* (-); KPC (-) \| \| *Pseudofusicoccum* sp. \| LGMF1611 \| MSSA (+);MRSA (-); *P. aeruginosa* (+)*; A. baumannii* (-); KPC (-) \| \| *Diaporthe* sp. \| LGMF1612 \| MSSA (+); MRSA (-); *P. aeruginosa* (-); *A. baumannii* (-); KPC (-) \| \| *Lasiodiplodia* sp. \| LGMF1534 \| MSSA (+); MRSA (-); *P. aeruginosa* (-); *A. baumannii* (-); KPC (-) \| \| *Hypoxylon* sp. \| LGMF1613 \| MSSA (+++); MRSA (+++); *P. aeruginosa* (++); *A. baumannii* (++); KPC (-) \| \| *Neofusicoccum* *brasiliense* \| LGMF1535 \| MSSA(+++); MRSA(+++); *P. aeruginosa*(+++); *A.* *baumannii* (+++)*;*KPC(++) \| \| Xylariaceae sp.5 \| LGMF1537 \| MSSA (+++); MRSA (+); *P. aeruginosa* (+)*; A. baumannii* (+); KPC (-) \| \| *Diaporthe* sp. \| LGMF1614 \| MSSA (+++); MRSA (+++); *P. aeruginosa* (+)*; A. baumannii* (+); KPC (-) \| \| *Diaporthe* sp. \| LGMF1538 \| MSSA (+); MRSA (-); *P. aeruginosa* (-); *A.* *baumannii* (-)*;* KPC (-) \| \| *Diaporthe* sp. \| LGMF1539 \| MSSA (+++); MRSA (+); *P. aeruginosa* (+)*; A. baumannii* (+); KPC (-) \| \| *Diaporthe* sp. \| LGMF1540 \| MSSA (+++); MRSA (+); *P. aeruginosa* (+)*; A. baumannii* (+); KPC (-) \| \| *Diaporthe* sp. \| LGMF1615 \| MSSA (++); MRSA (-); *P. aeruginosa* (+)*; A. baumannii* (+); KPC (-) \| \| *Diaporthe* sp. \| LGMF1616 \| MSSA (++); MRSA (-); *P. aeruginosa* (+)*; A. baumannii* (+); KPC (-) \| \| *Didymella* sp. \| LGMF1617 \| MSSA (++); MRSA (-); *P. aeruginosa* (+)*; A. baumannii* (+); KPC (-) \| \| *Diaporthe* *schini* \| LGMF1541 \| MSSA (+); MRSA (-)*; P. aeruginosa* (+); *A. baumannii* (-); KPC (-) \| \| Xylariaceae sp.3 \| LGMF1542 \| MSSA (++); MRSA (-); *P. aeruginosa* (-); *A.* *baumannii* (-)*;* KPC (-) \| \| *Diaporthe* sp. \| LGMF1618 \| MSSA (-); MRSA (-); *P. aeruginosa* (++); *A.* *baumannii* (+)*;* KPC (-) \| \| *Corynespora* *cambrensis* \| LGMF1619 \| MSSA (-); MRSA (-); *P. aeruginosa* (-); *A.* *baumannii* (-)*;* KPC (-) \| \| *Diaporthe* sp. \| LGMF1620 \| MSSA (-); MRSA (-); *P. aeruginosa* (-); *A.* *baumannii* (-)*;* KPC (-) \| \| *Colletotrichum* *siamense* \| LGMF1621 \| MSSA (-); MRSA (-); *P. aeruginosa* (-); *A.* *baumannii* (+)*;* KPC (-) \| \| *Fusarium* sp. \| LGMF1543 \| MSSA (++); MRSA (-); *P. aeruginosa* (+); *A.* *baumannii* (+)*;* KPC (-) \| \| Diatrypaceae sp. \| LGMF1544 \| MSSA (-); MRSA (-); *P. aeruginosa* (+); *A.* *baumannii* (+)*;* KPC (-) \| \| Xylariaceae sp.4 \| LGMF1545 \| MSSA (++); MRSA (-); *P. aeruginosa* (+); *A.* *baumannii* (+)*;* KPC (-) \| \| *Fusarium* sp. \| LGMF1546 \| MSSA (++); MRSA (-); *P. aeruginosa* (-); *A.* *baumannii* (-)*;* KPC (-) \| \| *Fusarium* sp. \| LGMF1547 \| MSSA (++); MRSA (-); *P. aeruginosa* (+); *A.* *baumannii* (+)*;* KPC (-) \| \| *Nigrospora* sp. \| LGMF1622 \| MSSA (-); MRSA (-); *P. aeruginosa* (-); *A.* *baumannii* (-)*;* KPC (-) \| \| *Diaporthe* sp. \| LGMF1548 \| MSSA(+++); MRSA (+++); *P. aeruginosa* (+++)*;A. baumannii* (++);KPC (-) \| \| *Diaporthe* sp. \| LGMF1549 \| MSSA (++)*;* MRSA (+)*; P. aeruginosa* (+); *A. baumannii* (-); KPC (-) \| \| *Daldinia* sp. \| LGMF1623 \| MSSA (++)*;* MRSA (+)*; P. aeruginosa* (-); *A. baumannii* (-); KPC (-) \| \| *Didymella* sp. \| LGMF1624 \| MSSA (+); MRSA (-); *P. aeruginosa* (-); *A.* *baumannii* (-)*;* KPC (-) \| \| *Alternaria* sp. \| LGMF1550 \| MSSA (-); MRSA (-); *P. aeruginosa* (-); *A.* *baumannii* (-)*;* KPC (-) \| \| *Pestalotiopsis* sp. \| LGMF1551 \| MSSA (-); MRSA (-); *P. aeruginosa* (-); *A.* *baumannii* (-)*;* KPC (-) \| \| *Pestalotiopsis* sp. \| LGMF1552 \| MSSA (++)*;* MRSA (+)*; P. aeruginosa* (+)*; A. baumannii* (-); KPC (-) \| \| *Colletotrichum* *siamense* \| LGMF1625 \| MSSA (-); MRSA (-); *P. aeruginosa* (-); *A.* *baumannii* (-)*;* KPC (-) \| \| *Diaporthe* sp. \| LGMF1626 \| MSSA (++)*;* MRSA (+)*; P. aeruginosa* (+)*; A. baumannii* (-); KPC (-) \| \| *Fusarium* sp. \| LGMF1553 \| MSSA (-); MRSA (-); *P. aeruginosa* (-); *A.* *baumannii* (-)*;* KPC (-) \| \| *Diaporthe* sp. \| LGMF1554 \| MSSA (+); MRSA (-); *P. aeruginosa* (-); *A.* *baumannii* (-)*;* KPC (-) \| \| *Lasiodiplodia* sp. \| LGMF1555 \| MSSA (+); MRSA (-); *P. aeruginosa* (-); *A.* *baumannii* (-)*;* KPC (-) \| \| *Bjerkandera* sp. \| LGMF1627 \| MSSA (-); MRSA (-); *P. aeruginosa* (-); *A.* *baumannii* (-)*;* KPC (-) \| \| *Epicoccum* sp. \| LGMF1557 \| MSSA (-); MRSA (-); *P. aeruginosa* (++); *A.* *baumannii* (+)*;* KPC (-) \| \| *Fusarium* sp. \| LGMF1558 \| MSSA (+); MRSA (-); *P. aeruginosa* (-); *A.* *baumannii* (-)*;* KPC (-) \| \| *Epicoccum* sp. \| LGMF1628 \| MSSA (-); MRSA (-); *P. aeruginosa* (-); *A.* *baumannii* (-)*;* KPC (-) \| \| *Diaporthe* sp. \| LGMF1629 \| MSSA (-); MRSA (-); *P. aeruginosa* (-); *A.* *baumannii* (-)*;* KPC (-) \| \| *Colletotrichum* sp. \| LGMF1559 \| MSSA (-); MRSA (-); *P. aeruginosa* (-); *A.* *baumannii* (-)*;* KPC (-) \| \| *Fusarium* sp. \| LGMF1560 \| MSSA (-); MRSA (-); *P. aeruginosa* (-); *A.* *baumannii* (-)*;* KPC (-) \| \| *Diaporthe* sp. \| LGMF1561 \| MSSA (-); MRSA (-); *P. aeruginosa* (-); *A.* *baumannii* (-)*;* KPC (-) \| \| *Neopestalotiopsis* sp. \| LGMF1562 \| MSSA (+); MRSA (-); *P. aeruginosa* (-); *A.* *baumannii* (-)*;* KPC (-) \| \| *Fusarium* sp. \| LGMF1630 \| MSSA (+++); MRSA (++); *P. aeruginosa* (+)*; A. baumannii* (+); KPC (+) \| \| *Diaporthe* sp. \| LGMF1563 \| MSSA (-); MRSA (-); *P. aeruginosa* (-); *A.* *baumannii* (-)*;* KPC (-) \| \| *Fusarium* sp. \| LGMF1564 \| MSSA (-); MRSA (-); *P. aeruginosa* (-); *A.* *baumannii* (-)*;* KPC (-) \| \| *Diaporthe* cf*. heveae* \| LGMF1631 \| MSSA (+++); MRSA (++); *P. aeruginosa* (+)*; A. baumannii* (+); KPC (+) \| \| *Diaporthe* sp. \| LGMF1632 \| MSSA (-); MRSA (-); *P. aeruginosa* (-); *A.* *baumannii* (-)*;* KPC (-) \| \| *Diaporthe* cf*. heveae* \| LGMF1565 \| MSSA (-); MRSA (-); *P. aeruginosa* (-); *A.* *baumannii* (-)*;* KPC (-) \| \| *Fusarium* sp. \| LGMF1566 \| MSSA (-); MRSA (-); *P. aeruginosa* (++); *A.* *baumannii* (-)*;* KPC (-) \| \| *Diaporthe* sp. \| LGMF1567 \| MSSA (-); MRSA (-); *P. aeruginosa* (-); *A.* *baumannii* (-)*;* KPC (-) \| \| *Fusarium* sp. \| LGMF1568 \| MSSA (++); MRSA (+); *P. aeruginosa* (-); *A.* *baumannii* (+)*;* KPC (-) \| \| *Diaporthe* cf*. heveae* \| LGMF1569 \| MSSA (-); MRSA (-); *P. aeruginosa* (-); *A.* *baumannii* (-)*;* KPC (-) \| \| *Epicoccum* \| LGMF1570 \| MSSA (-); MRSA (-); *P. aeruginosa* (-); *A.* *baumannii* (-)*;* KPC (-) \| \| *Colletotrichum* \| LGMF1571 \| MSSA (-); MRSA (-); *P. aeruginosa* (-); *A.* *baumannii* (-)*;* KPC (-) \| \| *Diaporthe* cf*. heveae* \| LGMF1633 \| MSSA (++); MRSA (+); *P. aeruginosa* (++); *A.* *baumannii* (+)*;* KPC (-) \| \| Xylariaceae sp.3 \| LGMF1572 \| MSSA (++); MRSA (-); *P. aeruginosa* (-); *A.* *baumannii* (+)*;* KPC (-) \| \| *Diaporthe* sp. \| LGMF1573 \| MSSA (-); MRSA (-); *P. aeruginosa* (-); *A.* *baumannii* (-)*;* KPC (-) \| \| Xylariaceae sp.4 \| LGMF1574 \| MSSA (-); MRSA (-); *P. aeruginosa* (-); *A.* *baumannii* (-)*;* KPC (-) \| \| *Neofusicoccum* *brasiliense* \| LGMF1634 \| MSSA (-); MRSA (-); *P. aeruginosa* (-); *A.* *baumannii* (-)*;* KPC (-) \| \| *Conidiochaeta nepalica* \| LGMF1575 \| MSSA (-); MRSA (-); *P. aeruginosa* (-); *A.* *baumannii* (-)*;* KPC (-) \| \| *Diaporthe* sp. \| LGMF1576 \| MSSA (-); MRSA (-); *P. aeruginosa* (-); *A.* *baumannii* (-)*;* KPC (-) \| \| *Diaporthe* cf*. heveae* \| LGMF1635 \| MSSA (+); MRSA (-); *P. aeruginosa* (-); *A.* *baumannii* (-)*;* KPC (-) \| \| *Colletotrichum* *boninense* \| LGMF1577 \| MSSA (-); MRSA (-); *P. aeruginosa* (-); *A.* *baumannii* (-)*;* KPC (-) \| \| *Bjerkandera* sp. \| LGMF1578 \| MSSA (-); MRSA (-); *P. aeruginosa* (-); *A.* *baumannii* (-)*;* KPC (-) \| \| *Bjerkandera* sp. \| LGMF1579 \| MSSA (-); MRSA (-); *P. aeruginosa* (-); *A.* *baumannii* (-)*;* KPC (-) \| \| *Colletotrichum* *siamense* \| LGMF1636 \| MSSA (-); MRSA (-); *P. aeruginosa* (-); *A.* *baumannii* (-)*;* KPC (-) \| \| *Pestalotiopsis* sp. \| LGMF1637 \| MSSA (-); MRSA (-); *P. aeruginosa* (-); *A.* *baumannii* (-)*;* KPC (-) \| \| *Colletotrichum gloeosporioides* \| LGMF1580 \| MSSA (-); MRSA (-); *P. aeruginosa* (-); *A.* *baumannii* (-)*;* KPC (-) \| \| *Pestalotiopsis* sp. \| LGMF1638 \| MSSA (-); MRSA (-); *P. aeruginosa* (-); *A.* *baumannii* (-)*;* KPC (-) \| \| *Efibula* sp. \| LGMF1581 \| MSSA (-); MRSA (-); *P. aeruginosa* (-); *A.* *baumannii* (-)*;* KPC (-) \| \| *Diaporthe* sp. \| LGMF1583 \| MSSA(+++); MRSA(+++);*P. aeruginosa* (+++)*; A.baumannii* (+++);KPC(++) \| \| *Diaporthe* sp. \| LGMF1639 \| MSSA (-); MRSA (-); *P. aeruginosa* (-); *A.* *baumannii* (-)*;* KPC (-) \| \| *Phaeophleospora* sp. \| LGMF1584 \| MSSA (-); MRSA (-); *P. aeruginosa* (-); *A.* *baumannii* (-)*;* KPC (-) \| \| *Diaporthe* sp. \| LGMF1585 \| MSSA (-); MRSA (-); *P. aeruginosa* (-); *A.* *baumannii* (-)*;* KPC (-) \| \| *Colletotrichum* *gloeosporioides* \| LGMF1586 \| MSSA (-); MRSA (-); *P. aeruginosa* (-); *A.* *baumannii* (-)*;* KPC (-) \| \| *Fusarium* sp. \| LGMF1587 \| MSSA (-); MRSA (-); *P. aeruginosa* (-); *A.* *baumannii* (-)*;* KPC (-) \| \| *Colletotrichum gloeosporioides* \| LGMF1588 \| MSSA (-); MRSA (-); *P. aeruginosa* (-); *A.* *baumannii* (-)*;* KPC (-) \| \| *Fusarium* sp. \| LGMF1589 \| MSSA (-); MRSA (-); *P. aeruginosa* (-); *A.* *baumannii* (-)*;* KPC (-) \| \| *Fusarium* sp. \| LGMF1590 \| MSSA (-); MRSA (-); *P. aeruginosa* (-); *A.* *baumannii* (-)*;* KPC (-) \| \| *Nigrospora* *hainanensis* \| LGMF1591 \| MSSA (+); MRSA (-); *P. aeruginosa* (-); *A.* *baumannii* (-)*;* KPC (-) \| \| *Colletotrichum* *boninense* \| LGMF1592 \| MSSA (-); MRSA (-); *P. aeruginosa* (-); *A.* *baumannii* (-)*;* KPC (-) \| \| *Diaporthe* cf*. heveae* \| LGMF1593 \| MSSA (-); MRSA (-); *P. aeruginosa* (-); *A.* *baumannii* (-)*;* KPC (-) \| \| *Diaporthe* cf*. heveae* \| LGMF1594 \| MSSA (+); MRSA (-); *P. aeruginosa* (-); *A.* *baumannii* (-)*;* KPC (-) \| \| *Colletotrichum* *siamense* \| LGMF1595 \| MSSA (-); MRSA (-); *P. aeruginosa* (-); *A.* *baumannii* (-)*;* KPC (-) \| \| *Epicoccum* sp. \| LGMF1641 \| MSSA (-); MRSA (-); *P. aeruginosa* (-); *A.* *baumannii* (-)*;* KPC (-) \| \| *Epicoccum* sp. \| LGMF1598 \| MSSA (+++); MRSA (+++); *P. aeruginosa* (-); *A.* *baumannii* (-)*;* KPC (-) \| \| *Epicoccum* sp. \| LGMF1600 \| MSSA (+++); MRSA (+++); *P. aeruginosa* (-); *A.* *baumannii* (-)*;* KPC (-) \| \| *Diaporthe* sp \| LGMF1601 \| MSSA (+); MRSA (-); *P. aeruginosa* (-); *A.* *baumannii* (-)*;* KPC (-) \| \| *Diaporthe* sp. \| LGMF1602 \| MSSA (+); MRSA (-); *P. aeruginosa* (-); *A.* *baumannii* (-)*;* KPC (-) \| \| *Diaporthe* sp. \| LGMF1508 \| MSSA (+); MRSA (-); *P. aeruginosa* (-); *A.* *baumannii* (-)*;* KPC (-) \| \| *Colletotrichum* *siamense* \| LGMF1604 \| MSSA (+); MRSA (-); *P. aeruginosa* (-); *A.* *baumannii* (-)*;* KPC (-) \| \| Pleomassariaceae sp. \| LGMF1509 \| MSSA (-); MRSA (-); *P. aeruginosa* (-); *A.* *baumannii* (-)*;* KPC (-) \| \| *Acrocalymma* *medicaginis* \| LGMF1605 \| MSSA (+); MRSA (-); *P. aeruginosa* (-); *A.* *baumannii* (-)*;* KPC (-) \| \| *Phyllosticta* sp. \| LGMF1511 \| MSSA (-); MRSA (-); *P. aeruginosa* (-); *A.* *baumannii* (-)*;* KPC (-) \| \| *Phyllosticta* sp. \| LGMF1512 \| MSSA (-); MRSA (-); *P. aeruginosa* (-); *A.* *baumannii* (-)*;* KPC (-) \| \| *Phaeophleospora* sp. \| LGMF1513 \| MSSA (+++); MRSA (+++); *P. aeruginosa* (-); *A.* *baumannii* (-)*;* KPC (-) \| \| *Paraphaeosphaeria* sp. \| LGMF1515 \| MSSA (-); MRSA (-); *P. aeruginosa* (-); *A.* *baumannii* (-)*;* KPC (-) \| \| *Paraphaeosphaeria* sp. \| LGMF1516 \| MSSA (+); MRSA (-); *P. aeruginosa* (-); *A.* *baumannii* (-)*;* KPC (-) \| \| *Diaporthe* sp. \| LGMF1517 \| MSSA (-); MRSA (-); *P. aeruginosa* (-); *A.* *baumannii* (-)*;* KPC (-) \| \| *Diaporthe* sp. \| LGMF1606 \| MSSA (+); MRSA (-); *P. aeruginosa* (-); *A.* *baumannii* (-)*;* KPC (-) \| \| *Roussoella* sp. \| LGMF1518 \| MSSA (+); MRSA (-); *P. aeruginosa* (-); *A.* *baumannii* (-)*;* KPC (-) \| \| *Pestalotiopsis* sp. \| LGMF1519 \| MSSA (+); MRSA (-); *P. aeruginosa* (-); *A.* *baumannii* (-)*;* KPC (-) \| \| *Phyllosticta* sp. \| LGMF1521 \| MSSA (-); MRSA (-); *P. aeruginosa* (-); *A.* *baumannii* (-)*;* KPC (-) \| \| *Colletotrichum boninense* \| LGMF1522 \| MSSA (-); MRSA (-); *P. aeruginosa* (-); *A.* *baumannii* (-)*;* KPC (-) \| \| *Phaeophleospora* sp. \| LGMF1524 \| MSSA (+); MRSA (-); *P. aeruginosa* (-); *A.* *baumannii* (-)*;* KPC (-) \| \| *Pseudofusicoccum* *stromaticcum* \| LGMF1608 \| MSSA (-); MRSA (-); *P. aeruginosa* (-); *A.* *baumannii* (-)*;* KPC (-) \| \| *Diaporthe schini* \| LGMF1525 \| MSSA (-); MRSA (-); *P. aeruginosa* (-); *A.* *baumannii* (-)*;* KPC (-) \| \| *Diaporthe* sp. \| LGMF1530 \| MSSA (-); MRSA (-); *P. aeruginosa* (-); *A.* *baumannii* (-)*;* KPC (-) \| \| *Diaporthe* sp. \| LGMF1531 \| MSSA (-); MRSA (-); *P. aeruginosa* (-); *A.* *baumannii* (-)*;* KPC (-) \| \| *Lasiodiplodia* sp. \| LGMF1609 \| MSSA (-); MRSA (-); *P. aeruginosa* (-); *A.* *baumannii* (-)*;* KPC (-) \| \| *Diaporthe* sp. \| LGMF1532 \| MSSA (-); MRSA (-); *P. aeruginosa* (-); *A.* *baumannii* (-)*;* KPC (-) \| |
| --- | --- | --- | --- | --- | --- | --- | --- | --- | --- | --- | --- | --- | --- | --- | --- | --- | --- | --- | --- | --- | --- | --- | --- | --- | --- | --- | --- | --- | --- | --- | --- | --- | --- | --- | --- | --- | --- | --- | --- | --- | --- | --- | --- | --- | --- | --- | --- | --- | --- | --- | --- | --- | --- | --- | --- | --- | --- | --- | --- | --- | --- | --- | --- | --- | --- | --- | --- | --- | --- | --- | --- | --- | --- | --- | --- | --- | --- | --- | --- | --- | --- | --- | --- | --- | --- | --- | --- | --- | --- | --- | --- | --- | --- | --- | --- | --- | --- | --- | --- | --- | --- | --- | --- | --- | --- | --- | --- | --- | --- | --- | --- | --- | --- | --- | --- | --- | --- | --- | --- | --- | --- | --- | --- | --- | --- | --- | --- | --- | --- | --- | --- | --- | --- | --- | --- | --- | --- | --- | --- | --- | --- | --- | --- | --- | --- | --- | --- | --- | --- | --- | --- | --- | --- | --- | --- | --- | --- | --- | --- | --- | --- | --- | --- | --- | --- | --- | --- | --- | --- | --- | --- | --- | --- | --- | --- | --- | --- | --- | --- | --- | --- | --- | --- | --- | --- | --- | --- | --- | --- | --- | --- | --- | --- | --- | --- | --- | --- | --- | --- | --- | --- | --- | --- | --- | --- | --- | --- | --- | --- | --- | --- | --- | --- | --- | --- | --- | --- | --- | --- | --- | --- | --- | --- | --- | --- | --- | --- | --- | --- | --- | --- | --- | --- | --- | --- | --- | --- | --- | --- | --- | --- | --- | --- | --- | --- | --- | --- | --- | --- | --- | --- | --- | --- | --- | --- | --- | --- | --- | --- | --- | --- | --- | --- | --- | --- | --- | --- | --- | --- | --- | --- | --- | --- | --- | --- | --- | --- | --- | --- | --- | --- | --- | --- | --- | --- | --- | --- | --- | --- | --- | --- | --- | --- | --- | --- | --- | --- | --- | --- | --- | --- | --- | --- | --- | --- | --- | --- | --- | --- | --- | --- | --- | --- | --- | --- | --- | --- | --- | --- | --- | --- | --- | --- | --- | --- | --- | --- | --- | --- | --- | --- | --- | --- | --- | --- | --- | --- | --- | --- | --- | --- | --- | --- | --- | --- | --- | --- | --- | --- | --- | --- | --- | --- | --- |

Note: MSSA= *Staphylococcus aureus* methicillin-sensitive; MRSA= *Staphylococcus aureus* methicillin-resistant; *P. aeruginosa*= *Pseudomonas aeruginosa*; *A. baumannii*= *Acinetobacter baumannii*; KPC= *Klebsiella pneumoniae* Carbapenemase. no activity (-), low (+), moderate (++) and high (+++) activity

Table S7-Extract yield performed in 250 mL in two culture media: ME (Malte extract) and CY (Czapeck).

| \| \| \| **Morphotype** \| **Identification** \| \| **Isolate** \| **Extract yield (mg)** \| \| \| --- \| --- \| --- \| --- \| --- \| --- \| \|  \| \|  \|  \| **ME** \| **CY** \| \| 6 \| \| *Hypoxylon* sp. \| LGMF1613 \| 97 \| 11 \| \| 7 \| \| *Neofusicoccum brasiliense* \| LGMF1535 \| 20 \| 2 \| \| 29 \| \| *Diaporthe* sp. \| LGMF1548 \| 41 \| 8 \| \| 51 \| \| *Fusarium* sp. \| LGMF1630 \| 11 \| 6 \| \| 54 \| \| *Diaporthe* cf*. heveae* \| LGMF1631 \| 42 \| 15 \| \| 80 \| \| *Diaporthe* sp. \| LGMF1583 \| 110 \| 31 \| \| 98 \| \| *Epicoccum* sp. \| LGMF1598 \| 21 \| 4 \| \| 100 \| \| *Epicoccum* sp \| LGMF1600 \| 11 \| 6 \| \| 104 \| \| *Colletotrichum siamense* \| LGMF1604 \| 100 \| 40 \| \| 109 \| \| *Phaeophleospora* sp \| LGMF1513 \| 18 \| 14 \| \| 121 \| \| *Diaporthe* sp. \| LGMF1530 \| 33 \| 4 \| \| \|  \| \| \| --- \| --- \| --- \| --- \| --- \| --- \| --- \| --- \| --- \| --- \| --- \| --- \| --- \| --- \| --- \| --- \| --- \| --- \| --- \| --- \| --- \| --- \| --- \| --- \| --- \| --- \| --- \| --- \| --- \| --- \| --- \| --- \| --- \| --- \| --- \| --- \| --- \| --- \| --- \| --- \| --- \| --- \| --- \| --- \| --- \| --- \| --- \| --- \| --- \| --- \| --- \| --- \| --- \| --- \| --- \| --- \| --- \| --- \| --- \| --- \| --- \| --- \| --- \| --- \| --- \| --- \| --- \| --- \| --- \| --- \| --- \| --- \| --- \| --- \| --- \| --- \| --- \| --- \| --- \| --- \| --- \| --- \| \|  \|  \| \|  \|  \|  \|  \| \|  \|  \|  \|  \| \| \| --- \| --- \| --- \| --- \| --- \| --- \| --- \| --- \| --- \| --- \| --- \| --- \| --- \| --- \| --- \| --- \| --- \| --- \| --- \| --- \| --- \| --- \| --- \| --- \| --- \| --- \| --- \| --- \| --- \| --- \| --- \| --- \| --- \| --- \| --- \| --- \| --- \| --- \| --- \| --- \| --- \| --- \| --- \| --- \| --- \| --- \| --- \| --- \| --- \| --- \| --- \| --- \| --- \| --- \| --- \| --- \| --- \| --- \| --- \| --- \| --- \| --- \| --- \| --- \| --- \| --- \| --- \| --- \| --- \| --- \| --- \| --- \| --- \| --- \| --- \| --- \| --- \| --- \| --- \| --- \| --- \| --- \| --- \| --- \| --- \| --- \| --- \| --- \| --- \| --- \| --- \| --- \| --- \| |
| --- | --- | --- | --- | --- | --- | --- | --- | --- | --- | --- | --- | --- | --- | --- | --- | --- | --- | --- | --- | --- | --- | --- | --- | --- | --- | --- | --- | --- | --- | --- | --- | --- | --- | --- | --- | --- | --- | --- | --- | --- | --- | --- | --- | --- | --- | --- | --- | --- | --- | --- | --- | --- | --- | --- | --- | --- | --- | --- | --- | --- | --- | --- | --- | --- | --- | --- | --- | --- | --- | --- | --- | --- | --- | --- | --- | --- | --- | --- | --- | --- | --- | --- | --- | --- | --- | --- | --- | --- | --- | --- | --- | --- | --- |

| Table S8-Inhibition zone of the crud extracts produced by endophytic fungi against clinical pathogens.   \| **Strain/Genera** \| **Antimicrobial activity (inhibition zone in mm)** \| \| \| \| \| \| \| \| \| \| \| \| \| \| \| \| \| --- \| --- \| --- \| --- \| --- \| --- \| --- \| --- \| --- \| --- \| --- \| --- \| --- \| --- \| --- \| --- \| --- \| \| MRSA \| \| MSSA \| \| *S. maltophilia* \| \| KPC \| \| *E. cloacae* \| \| *A. baumannii* \| \| *P. aeruginosa* \| \| *C. albicans* \| \| \|  \| ME \| CY \| ME \| CY \| ME \| CY \| ME \| CY \| ME \| CY \| ME \| CY \| ME \| CY \| ME \| CY \| \| *Colletotrichum* LGMF1604 \| 15 ±2 \| 9.5±0.7 \| 9.5 ± 0.7 \| 0 \| 11 ± 0.7 \| 13±1.4 \| 10 ± 1.4 \| 10.5±0.7 \| 10 ± 0 \| 0 \| 0 \| 0 \| 9.5 ± 0.7 \| 11±0.7 \| 10 ± 2 \| 10±0 \| \| *Diaporthe* LGMF1530 \| 15 ±2 \| 0 \| 10 ± 1.4 \| 11.5±2.1 \| 11 ± 0.7 \| 13±1.4 \| 11 ± 0 \| 13±0 \| 14 ± 1.7 \| 11.5±0.7 \| 0 \| 9.5±0.7 \| 10 ± 0.7 \| 10±0 \| 12 ± 0 \| 13±0 \| \| *Diaporthe* LGMF1548 \| 14 ±1.4 \| 15±0 \| 23 ± 0.7 \| 11.5±2.1 \| 17 ± 3.5 \| 12±1.4 \| 18 ± 0 \| 14±1.4 \| 19 ± 1.4 \| 11.5±2 \| 0 \| 0 \| 16 ± 0.7 \| 10±0 \| 9.5 ± 0.7 \| 9.5±0.7 \| \| *Diaporthe* LGMF1583 \| 17 ±2 \| 10±0 \| 23 ± 2.8 \| 10±0 \| 14 ± 1.4 \| 13±1.4 \| 14 ± 0.7 \| 11.5±0.7 \| 14 ± 0.7 \| 14 ± 0.7 \| 0 \| 0 \| 16 ± 0 \| 9,0±0 \| 19 ± 1.4 \| 14±1.4 \| \| *Diaporthe* LGMF1631 \| 10 ±0 \| 0 \| 11 ± 1.4 \| 0 \| 12 ± 0.7 \| 0 \| 11 ± 1.4 \| 0 \| 16 ± 2.8 \| 0 \| 0 \| 0 \| 0 \| 0 \| 13 ± 0.7 \| 0 \| \| *Epicoccum* LGMF1598 \| 11 ±0.7 \| 9±0 \| 11 ± 0.7 \| 0 \| 16 ± 0.7 \| 11.5±0.7 \| 14 ± 1.4 \| 11.5±0.7 \| 11 ± 1.4 \| 14±1.4 \| 0 \| 0 \| 9 ± 0 \| 0 \| 11 ± 0.7 \| 9.5±0.7 \| \| *Epicoccum* LGMF1600 \| 9.5 ±0.7 \| 9.5±0.7 \| 9.7 ± 0.7 \| 9.5±0.7 \| 15 ± 1.4 \| 10.5±0.7 \| 9.5 ± 0.7 \| 9.5±0.7 \| 10 ± 0 \| 11.5±2 \| 15 ± 0.7 \| 10.5±0.7 \| 10. ± 0 \| 0 \| 11 ± 1.4 \| 10.5±0.7 \| \| *Fusarium* LGMF1630 \| 10±0 \| 15±0 \| 9.0±0 \| 11.5±2.1 \| 10±0 \| 13±1.4 \| 15±0 \| 13±0 \| 1,25±0,07 \| 13.5±2 \| 0 \| 0 \| 0,5±0,7 \| 12.5±0.7 \| 0 \| 13±1.4 \| \| *Hypoxylon* LGMF1613 \| 11±2 \| 0 \| 10±0 \| 0 \| 13±2 \| 0 \| 12,5±0.7 \| 0 \| 13±1.4 \| 0 \| 0 \| 0 \| 0 \| 0 \| 0 \| 0 \| \| *Neofusicoccum* LGMF1535 \| 23 ±2**^2^** \| 0 \| 20 ± 0 \| 16±0.7 \| 14 ± 0.7 \| 11.5±2 \| 16 ± 0.7 \| 11±1.4 \| 16 ± 1.4 \| 12.5±0.7 \| 0 \| 0 \| 17 ± 0.7 \| 11±1.4 \| 14 ± 0 \| 15 ± 0 \| \| *Phaeophleospora* LGMF1513 \| 11±1.4 \| 1.1±0.14 \| 11.5±2.1 \| 11±0 \| 15±1.4 \| 15±1.4 \| 12.5±0.7 \| 9.5±0.7 \| 14±0.7 \| 12±0.7 \| 11.5±0.7 \| 0 \| 11.5±0.7 \| 0 \| 13±1.4 \| 12 ± 0 \| \| Antibiotic \| 25±0 \| \|  \|  \| 20±0 \| \| 20±0 \| \| 20±0 \| \| 30±0 \| \| 25±0 \| \| 20±0 \| \| |
| --- | --- | --- | --- | --- | --- | --- | --- | --- | --- | --- | --- | --- | --- | --- | --- | --- | --- | --- | --- | --- | --- | --- | --- | --- | --- | --- | --- | --- | --- | --- | --- | --- | --- | --- | --- | --- | --- | --- | --- | --- | --- | --- | --- | --- | --- | --- | --- | --- | --- | --- | --- | --- | --- | --- | --- | --- | --- | --- | --- | --- | --- | --- | --- | --- | --- | --- | --- | --- | --- | --- | --- | --- | --- | --- | --- | --- | --- | --- | --- | --- | --- | --- | --- | --- | --- | --- | --- | --- | --- | --- | --- | --- | --- | --- | --- | --- | --- | --- | --- | --- | --- | --- | --- | --- | --- | --- | --- | --- | --- | --- | --- | --- | --- | --- | --- | --- | --- | --- | --- | --- | --- | --- | --- | --- | --- | --- | --- | --- | --- | --- | --- | --- | --- | --- | --- | --- | --- | --- | --- | --- | --- | --- | --- | --- | --- | --- | --- | --- | --- | --- | --- | --- | --- | --- | --- | --- | --- | --- | --- | --- | --- | --- | --- | --- | --- | --- | --- | --- | --- | --- | --- | --- | --- | --- | --- | --- | --- | --- | --- | --- | --- | --- | --- | --- | --- | --- | --- | --- | --- | --- | --- | --- | --- | --- | --- | --- | --- | --- | --- | --- | --- | --- | --- | --- | --- | --- | --- | --- | --- | --- | --- | --- | --- | --- | --- | --- | --- | --- | --- | --- | --- | --- | --- | --- | --- | --- | --- | --- | --- | --- | --- | --- | --- | --- | --- | --- | --- | --- | --- | --- | --- | --- | --- | --- | --- | --- | --- | --- | --- | --- | --- | --- | --- | --- |


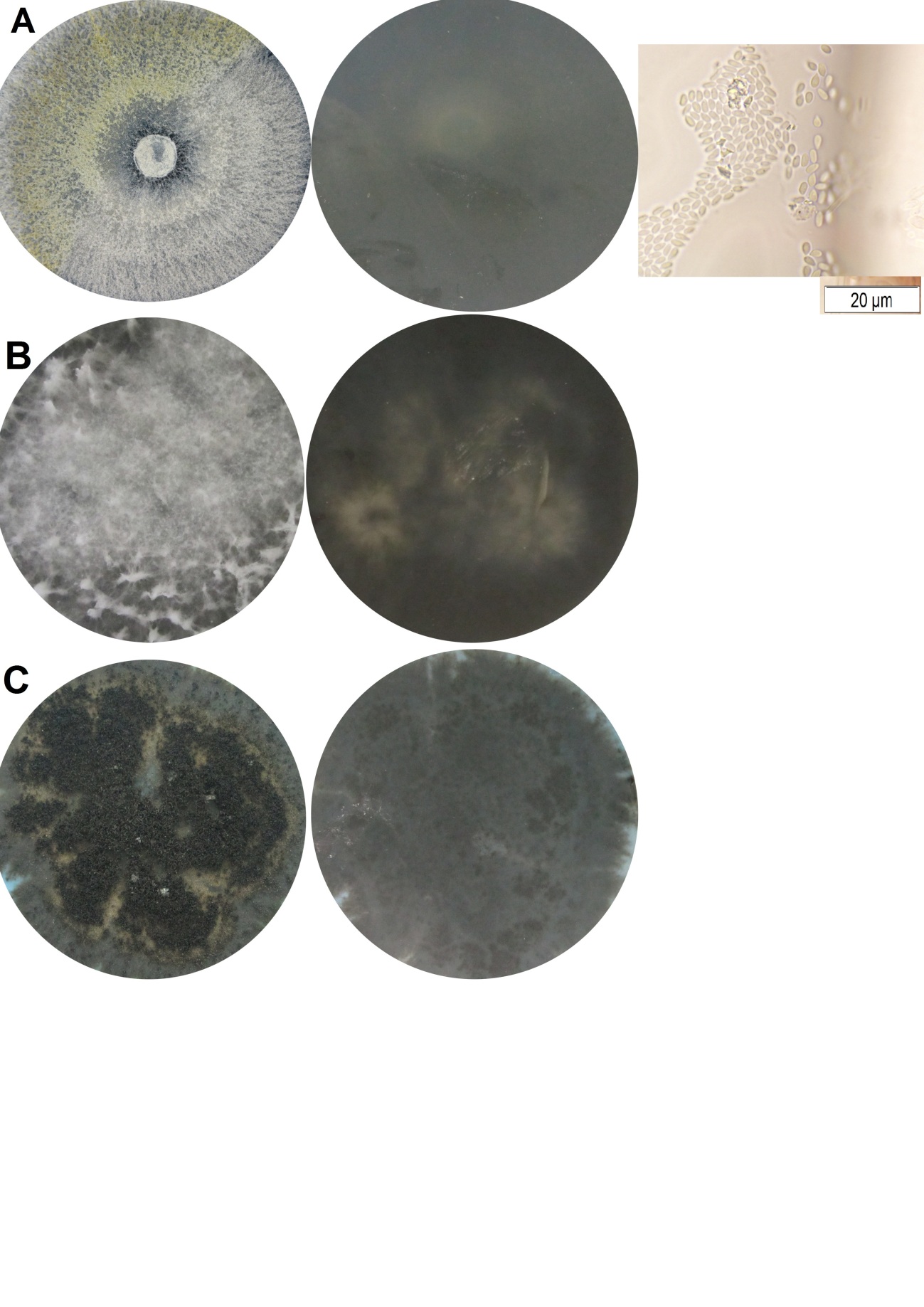


Figure S1-Morphological aspect of the dominant endophytic fungi found in this study. For details on the source (plant species), see Table 1. A: *Diaporthe schini* (morphotype 17), B: *Neofusicoccum brasiliense* (morphotype 7), C: *Phyllosticta* sp. (morphotype 116).


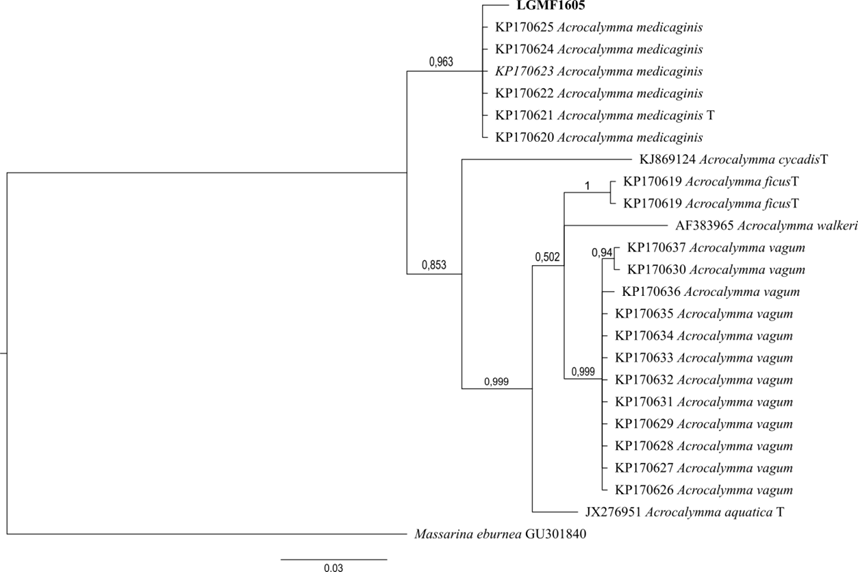


Figure S2-Bayesian phylogenetic tree based on ITS partial sequence of LGMF1605 (bold) identified as *Acrocalymma medicaginis* and sequences of all accepted species from *Acrocalymma* genus. The data matrix had 25 taxa and 465 characters. The tree was rooted to *Massarina* *eburnea* (AF383959). Scale bar shows 0.03 changes and Bayesian posterior probability values are indicated at the nodes. T: type strain.


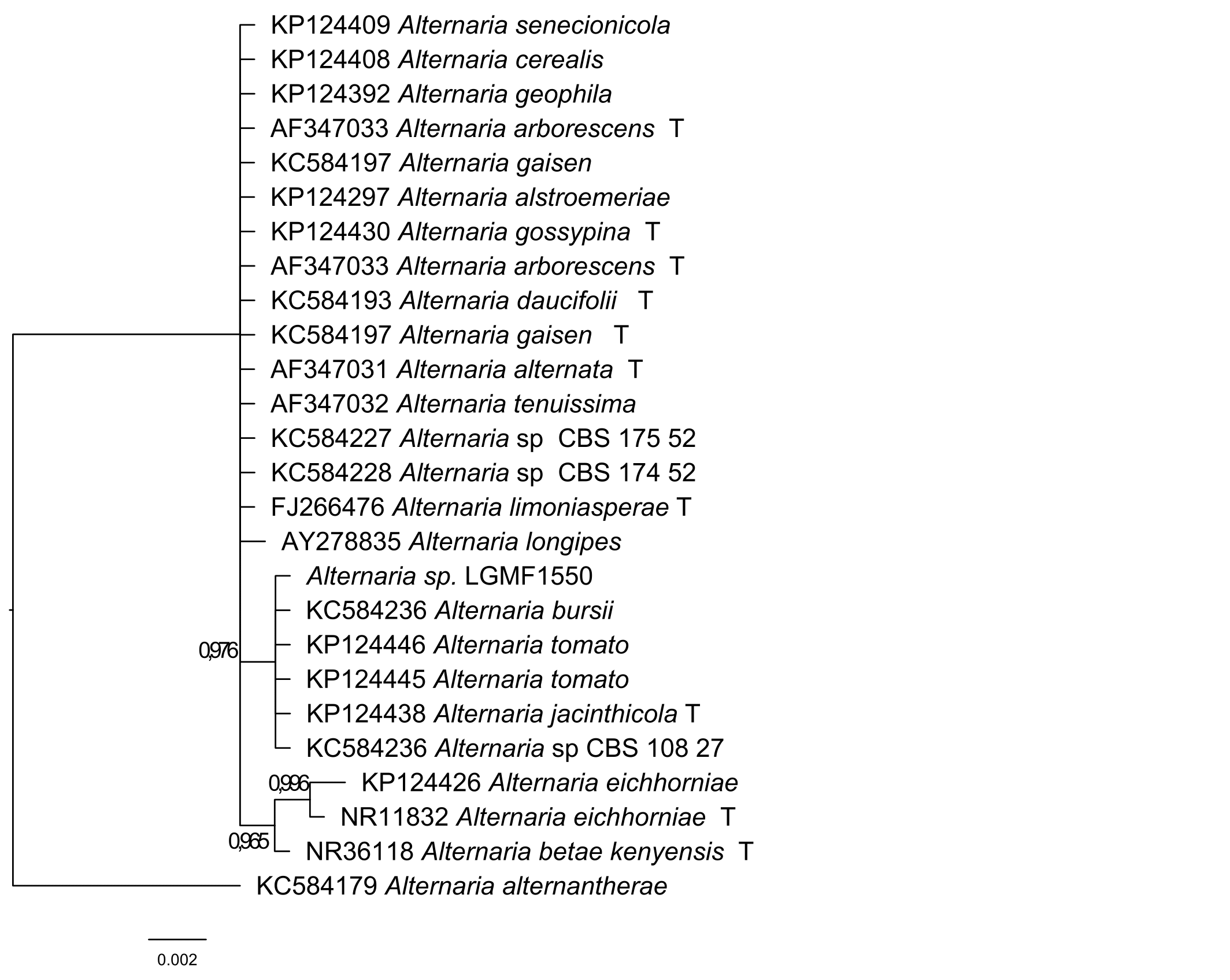


**Figure S3**-Bayesian phylogenetic tree based on ITS partial sequence of LGMF1550 (bold) identified as *Alternaria* sp., and sequence of all species allocated in *Alternaria* sect. *Alternaria*. The data matrix had 12 taxa and 500 characters. The tree was rooted to *Alternaria* *alternantherae* (KC584179). Scale bar shows 0.003 changes and Bayesian posterior probability values are indicated at the nodes. T: type strain.


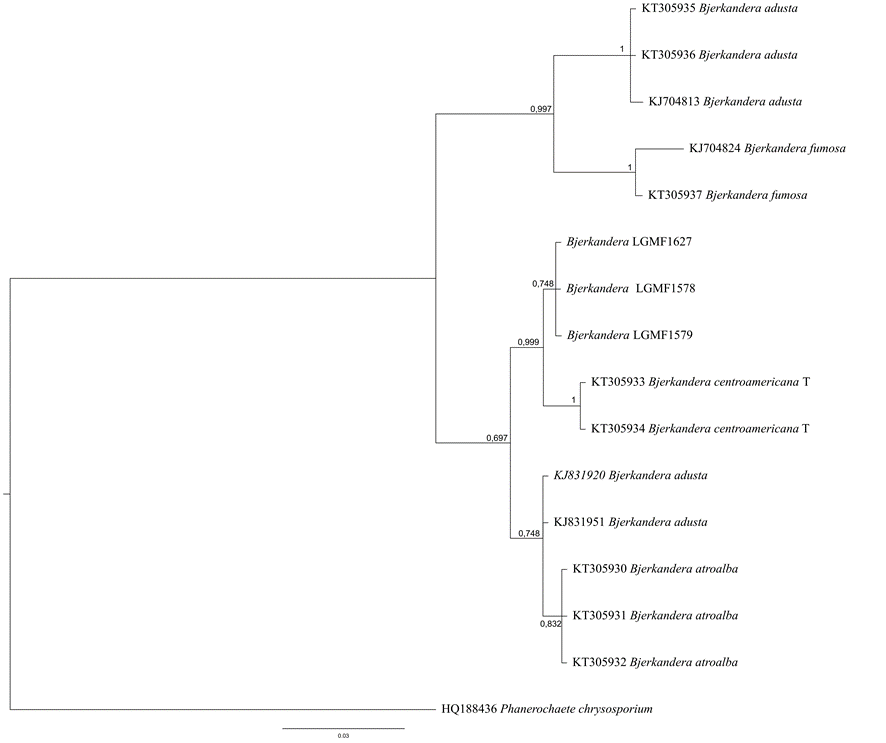


**Figure S4-**Bayesian phylogenetic tree based on ITS partial sequence of LGMF1627, LGMF1578 and LGMF1579 (bold) identified as *Bjerkandera* sp. and sequences of all accepted species from *Bjerkandera*. The tree was rooted to *Phanerochaete chrysosporiu* (HQ188436). Scale bar shows 0.03 changes and Bayesian posterior probability values are indicated at the nodes. T: type strain**.**

**
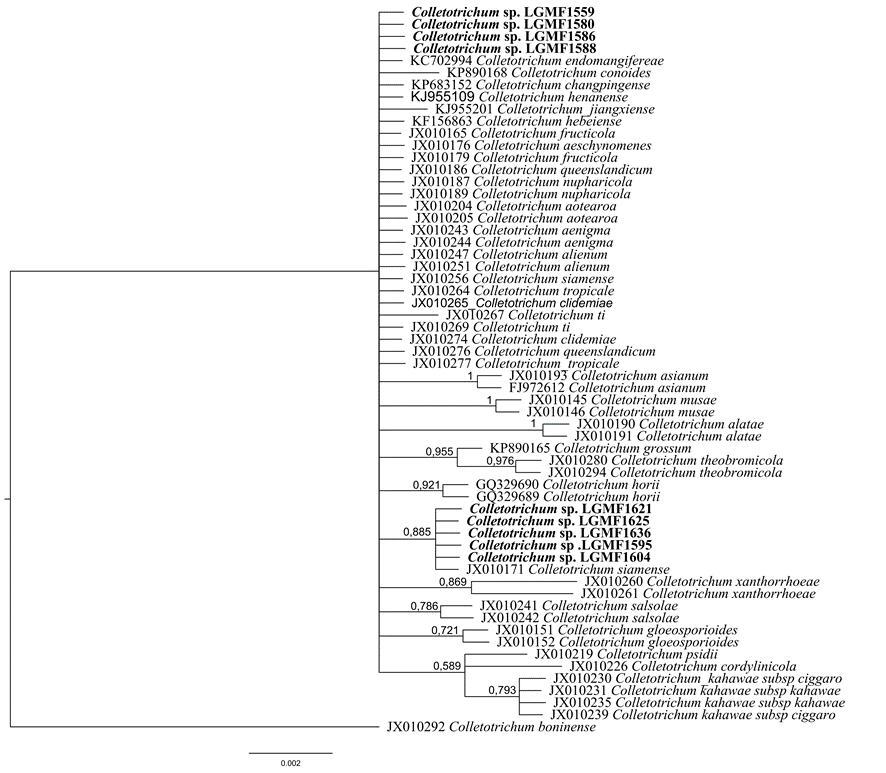
**

**Figure S5-**Bayesian phylogenetic tree based on ITS partial sequence of LGMF1559, LGMF1580 LGMF1586, LGMF1621, LGMF1625, LGMF1636, LGMF1595 and LGMF1604 (bold) identified as *Colletotrichum gloeosporioides* sensu lato and sequences of all species of *Colletotrichum* *gloeosporioides* species complex. The data matrix had 60 taxa and 407 characters. The tree was rooted to *Colletotrichum boninense* (JX010292). Scale bar shows 0.03 changes and Bayesian posterior probability values are indicated at the nodes. T: type strain.


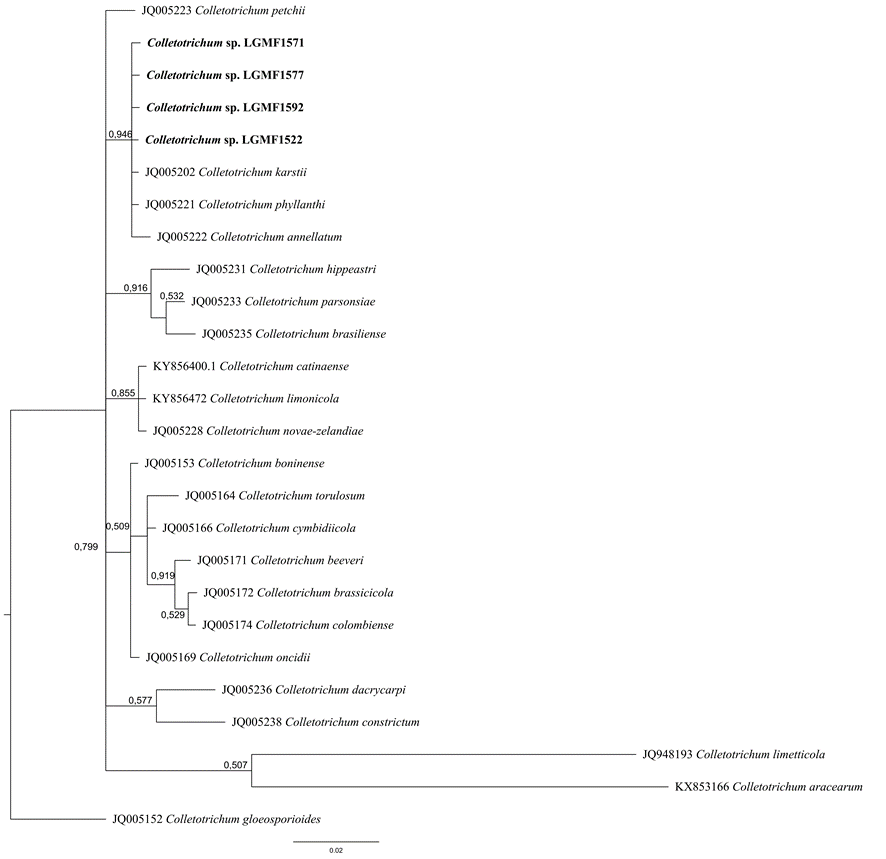


**Figure S6-**Bayesian phylogenetic tree based on ITS partial sequence of LGMF1571, LGMF1577, LGMF1592, and LGMF1522 (bold) identified as *Colletotrichum* *boninense* sensu lato and sequences of all accepted species from *Colletotrichum* *boninense* species complex. The data matrix had 26 taxa and 312 characters. The tree was rooted to *Colletotrichum gloeosporioides* (JQ005152). Scale bar shows 0.002 changes and Bayesian posterior probability values are indicated at the nodes. T: type strain.


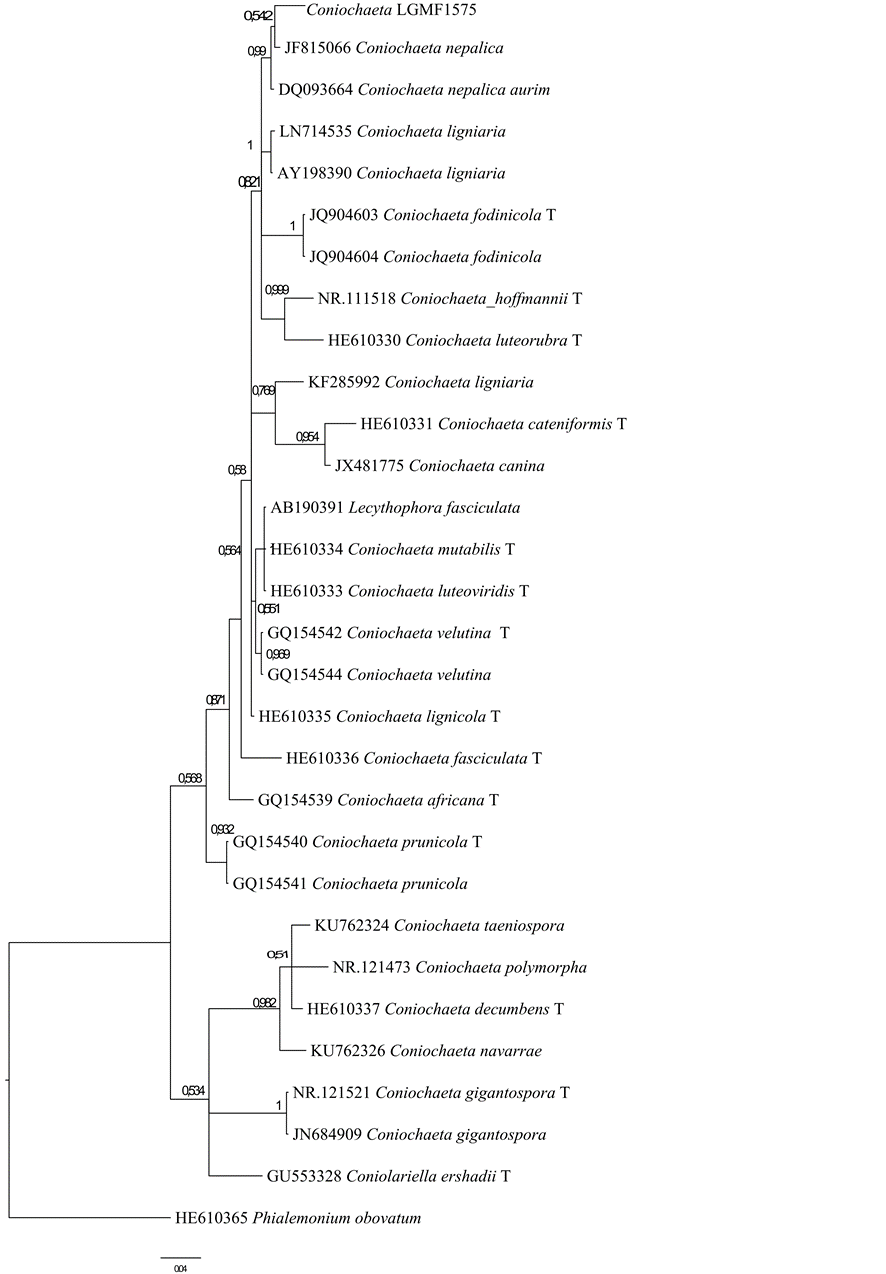


Figure S7-Bayesian phylogenetic tree based on ITS partial sequence of LGMF1575 (bold) identified as *Coniochaeta* sp. and sequences of all accepted species from *Coniochaeta* genus. The data matrix had 30 taxa and 488 characters. The tree was rooted to *Phialemonium obovatum* (HE610365). Scale bar shows 0.004 changes and Bayesian posterior probability values are indicated at the nodes. T: type strain.


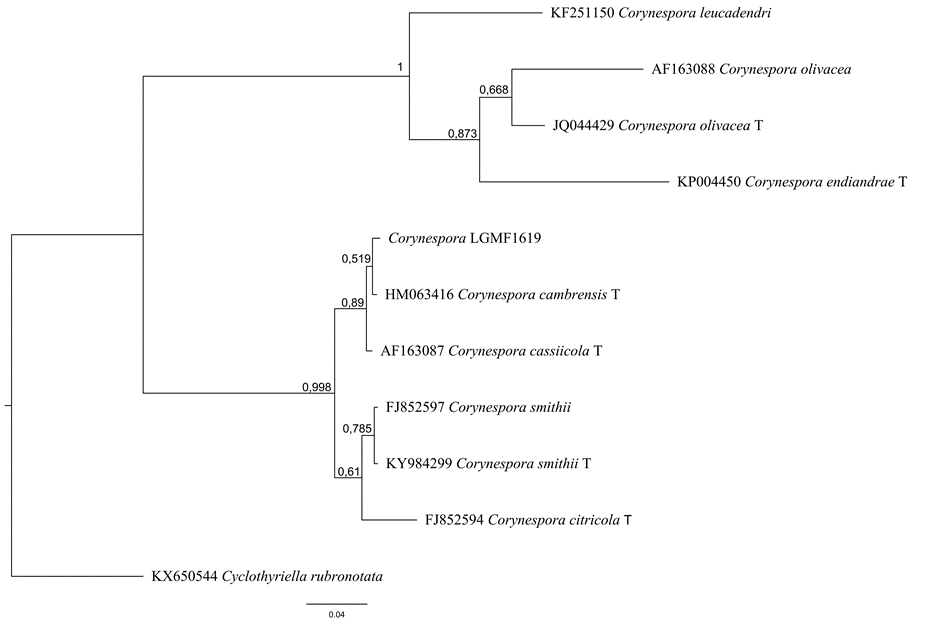


Figure S8-Bayesian phylogenetic tree based on ITS partial sequence of LGMF1619 (bold) identified as *Corynespora cambrensis* and sequences of all accepted species from *Corynespora* genus. The data matrix had 11 taxa and 409 characters. The tree was rooted to *Cyclothyriella rubronotata* (KX650544). Scale bar shows 0.04 changes and Bayesian posterior probability values are indicated at the nodes T: type strain.


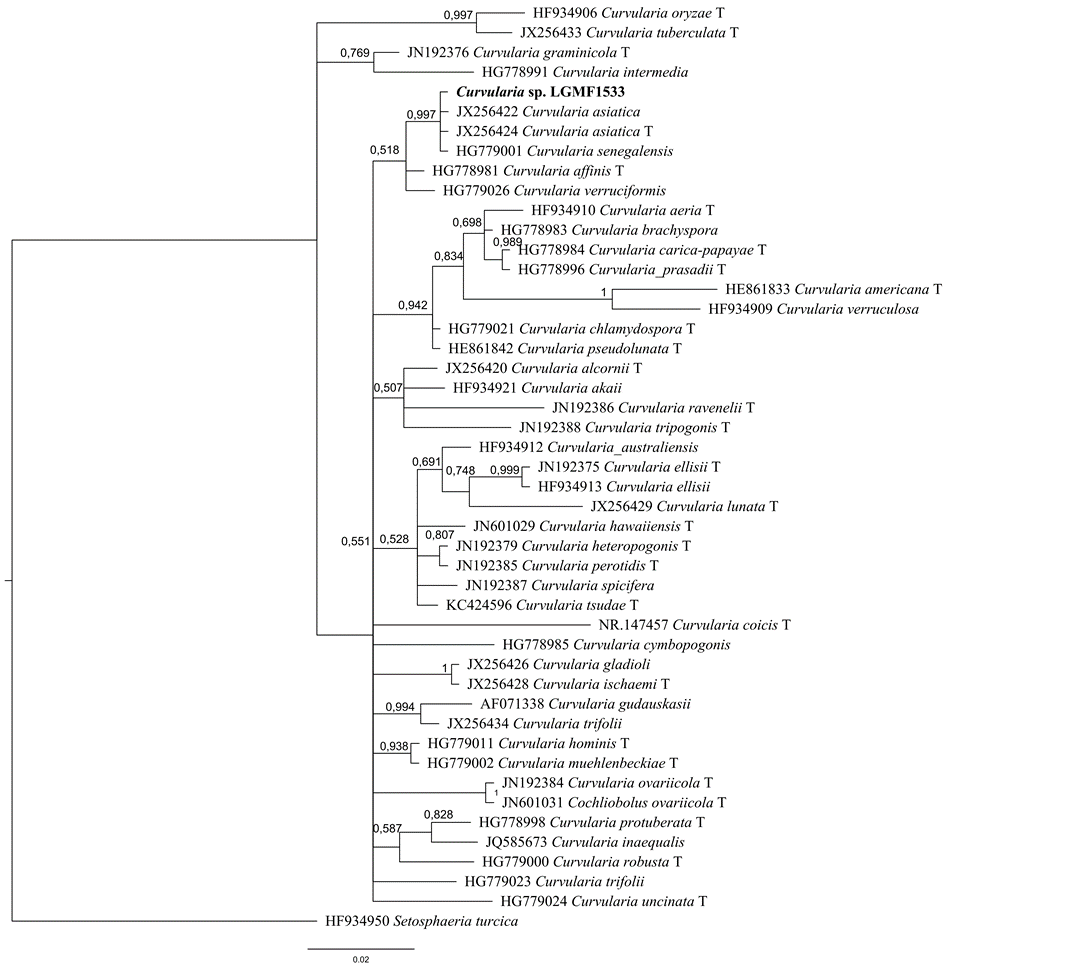


Figure S9-Bayesian phylogenetic tree based on ITS partial sequence of LGMF1533 (bold) identified as *Curvularia* sp. and sequences of all accepted species from *Curvularia* genus. The data matrix had 47 taxa and 491 characters. The tree was rooted to *Setosphaeria turcica* (HF934950). Scale bar shows 0.02 changes and Bayesian posterior probability values are indicated at the nodes. T: type strain.


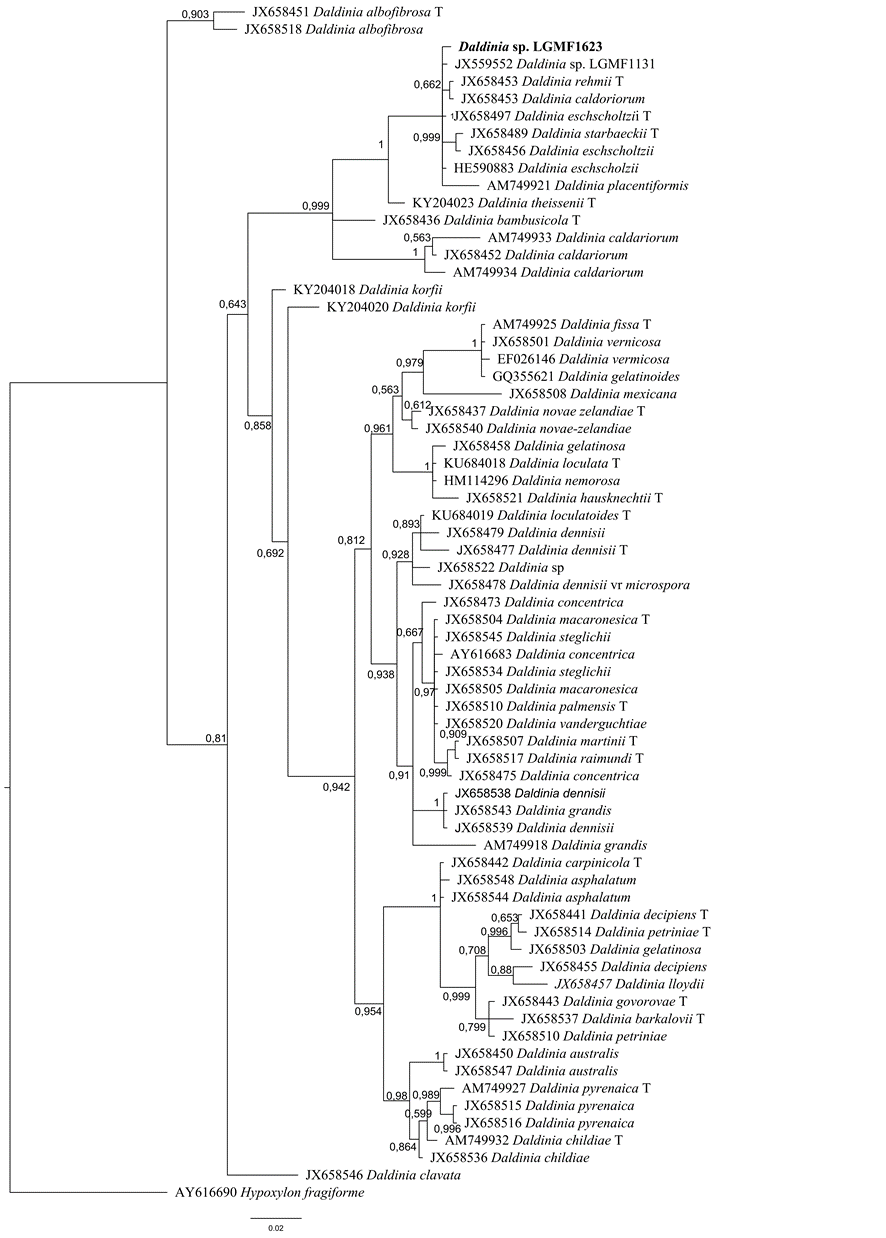


Figure S10-Bayesian phylogenetic tree based on ITS partial sequence of LGMF1623 (bold) identified as *Daldinia* sp. and sequences of all accepted species from *Daldinia* genus. The data matrix had 69 taxa and 522 characters. The tree was rooted to *Hypoxylon fragiforme* (AY616690). Scale bar shows 0.02 changes and Bayesian posterior probability values are indicated at the nodes. T: type strain


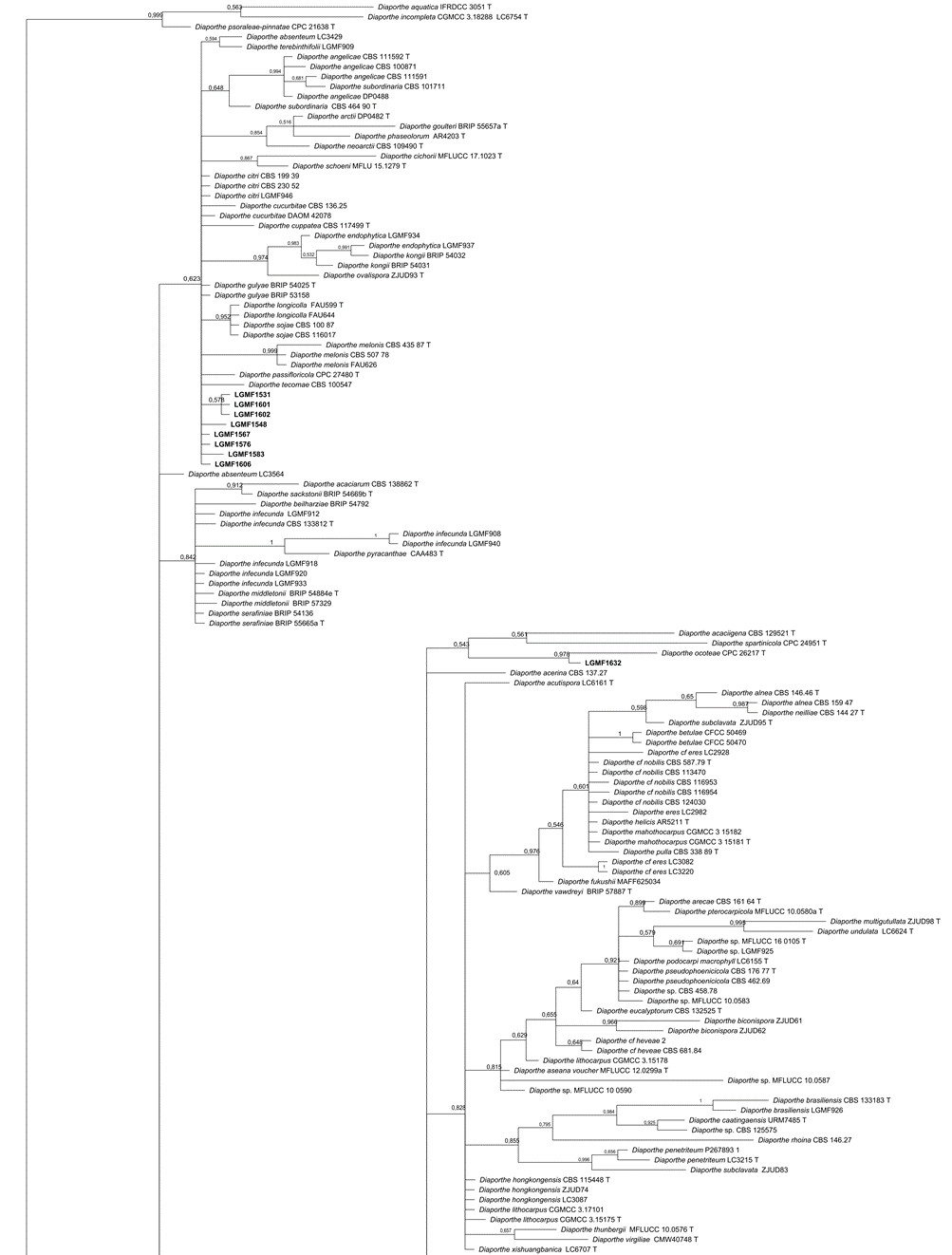


Figure S11-Bayesian phylogenetic tree based on ITS partial sequence of LGMF1610, LGMF1612, LGMF1614, 1538, LGMF1539, LGMF1540, LGMF1615, LGMF1616, LGMF1541, LGMF1618, LGMF1620, LGMF1548, 1549, LGMF1554, LGMF1629, LGMF 1561, LGMF1563, LGMF1631, LGMF1632, LGMF 1565, LGMF1567, LGMF 1569, LGMF 1633, LGMF1573, LGMF1576, LGMF1635, LGMF1583, LGMF1639, LGMF1593, LGMF1594, LGMF1601, LGMF1602, LGMF1508, LGMF1517, LGMF1606, LGMF1525, LGMF1526, LGMF1530, LGMF1531, LGMF1532, and LGMF1526 (bold) sequences of all accepted species from *Diaporthe* genus. The data matrix had 383 taxa and 537 characters. The tree was rooted to *Diaporthella* *corylina* (KC343004). T: type strain. (Continue)


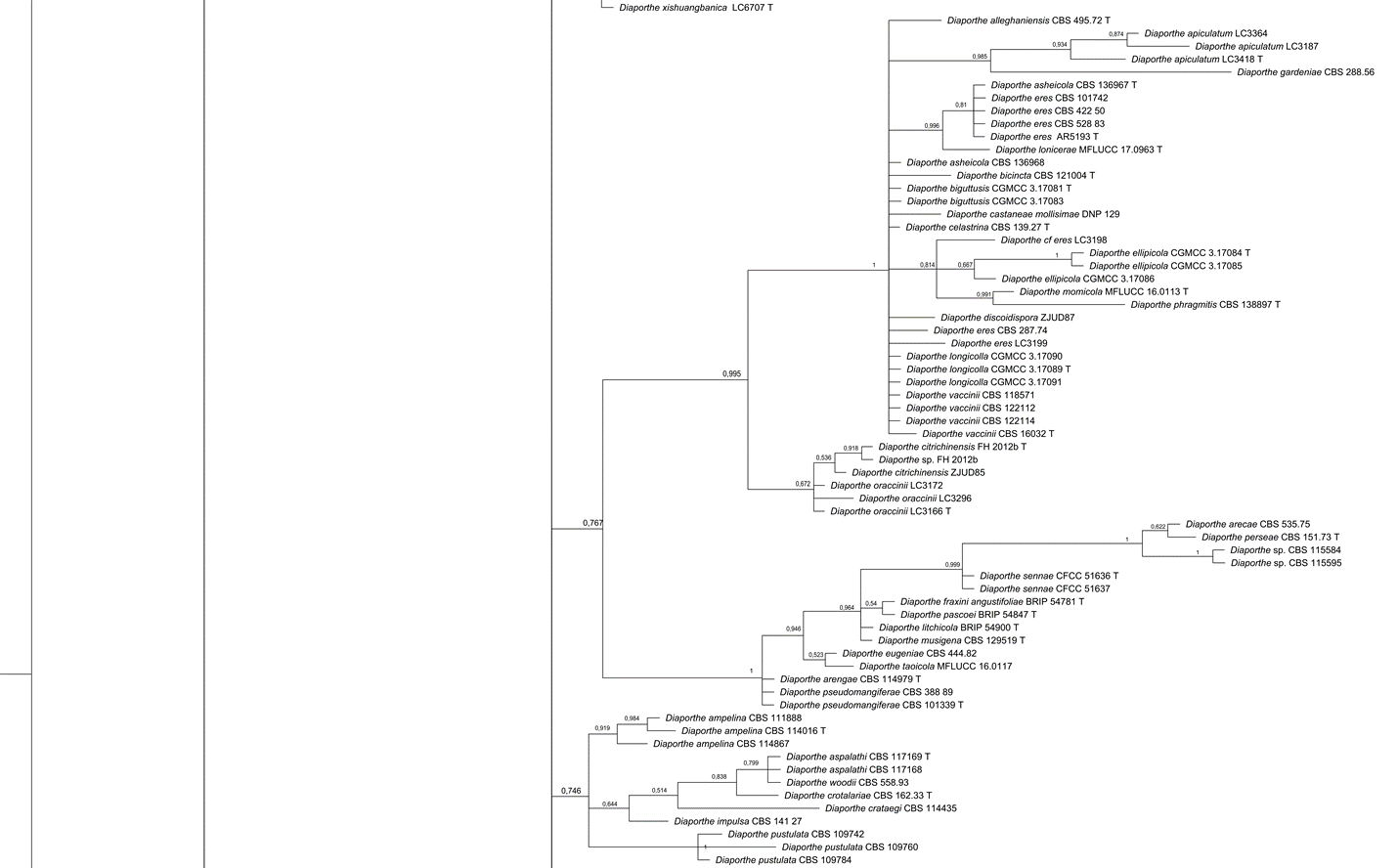


Figure S11-Bayesian phylogenetic tree based on ITS partial sequence of LGMF1610, LGMF1612, LGMF1614, 1538, LGMF1539, LGMF1540, LGMF1615, LGMF1616, LGMF1541, LGMF1618, LGMF1620, LGMF1548, 1549, LGMF1554, LGMF1629, LGMF 1561, LGMF1563, LGMF1631, LGMF1632, LGMF 1565, LGMF1567, LGMF 1569, LGMF 1633, LGMF1573, LGMF1576, LGMF1635, LGMF1583, LGMF1639, LGMF1593, LGMF1594, LGMF1601, LGMF1602, LGMF1508, LGMF1517, LGMF1606, LGMF1525, LGMF1526, LGMF1530, LGMF1531, LGMF1532, and LGMF1526 (bold) sequences of all accepted species from *Diaporthe* genus. The data matrix had 383 taxa and 537 characters. The tree was rooted to *Diaporthella* *corylina* (KC343004). T: type strain. (Continue)


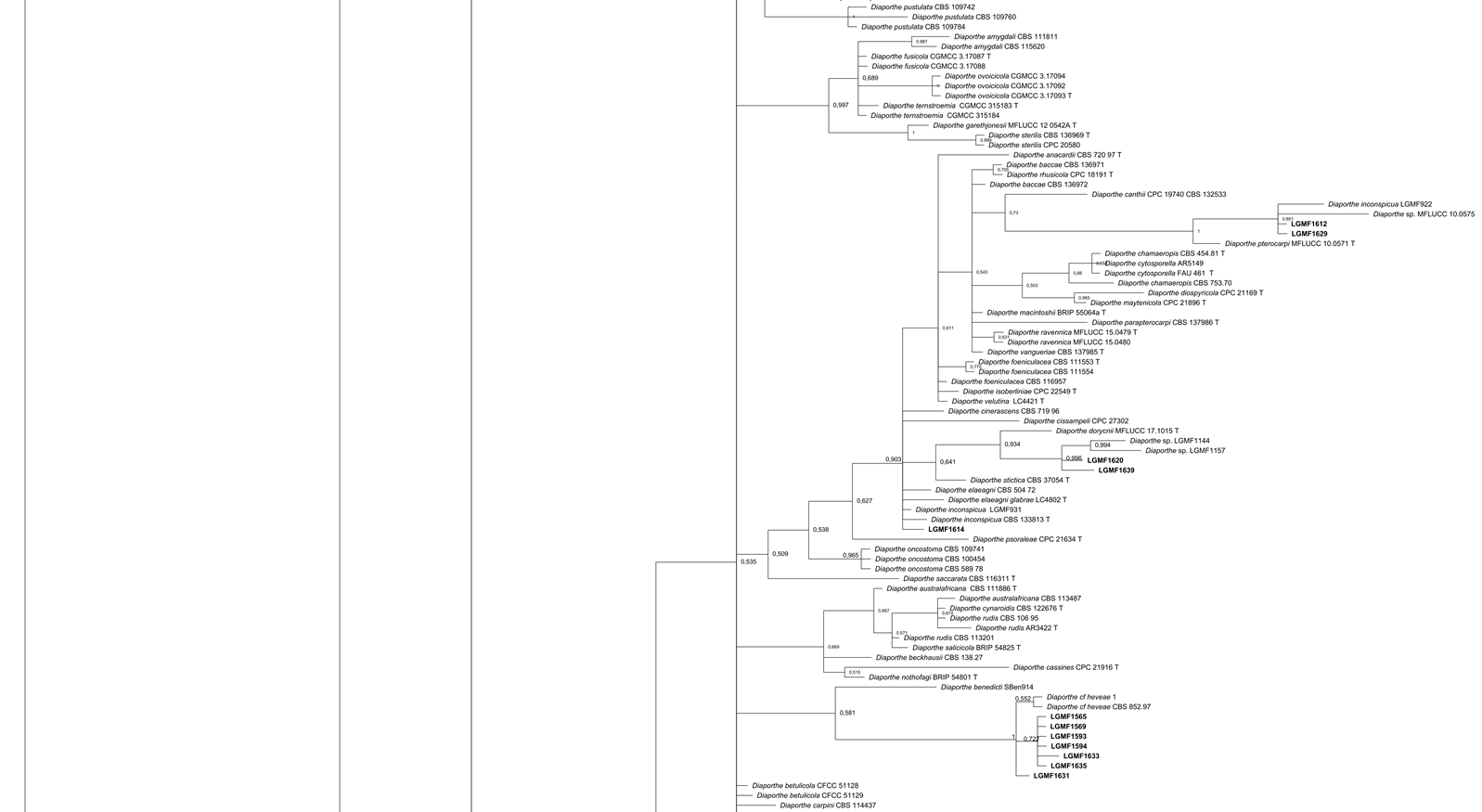


***Clade 1***

Figure S11-Bayesian phylogenetic tree based on ITS partial sequence of LGMF1610, LGMF1612, LGMF1614, 1538, LGMF1539, LGMF1540, LGMF1615, LGMF1616, LGMF1541, LGMF1618, LGMF1620, LGMF1548, 1549, LGMF1554, LGMF1629, LGMF 1561, LGMF1563, LGMF1631, LGMF1632, LGMF 1565, LGMF1567, LGMF 1569, LGMF 1633, LGMF1573, LGMF1576, LGMF1635, LGMF1583, LGMF1639, LGMF1593, LGMF1594, LGMF1601, LGMF1602, LGMF1508, LGMF1517, LGMF1606, LGMF1525, LGMF1526, LGMF1530, LGMF1531, LGMF1532, and LGMF1526 (bold) sequences of all accepted species from *Diaporthe* genus. The data matrix had 383 taxa and 537 characters. The tree was rooted to *Diaporthella* *corylina* (KC343004). T: type strain. (Continue)


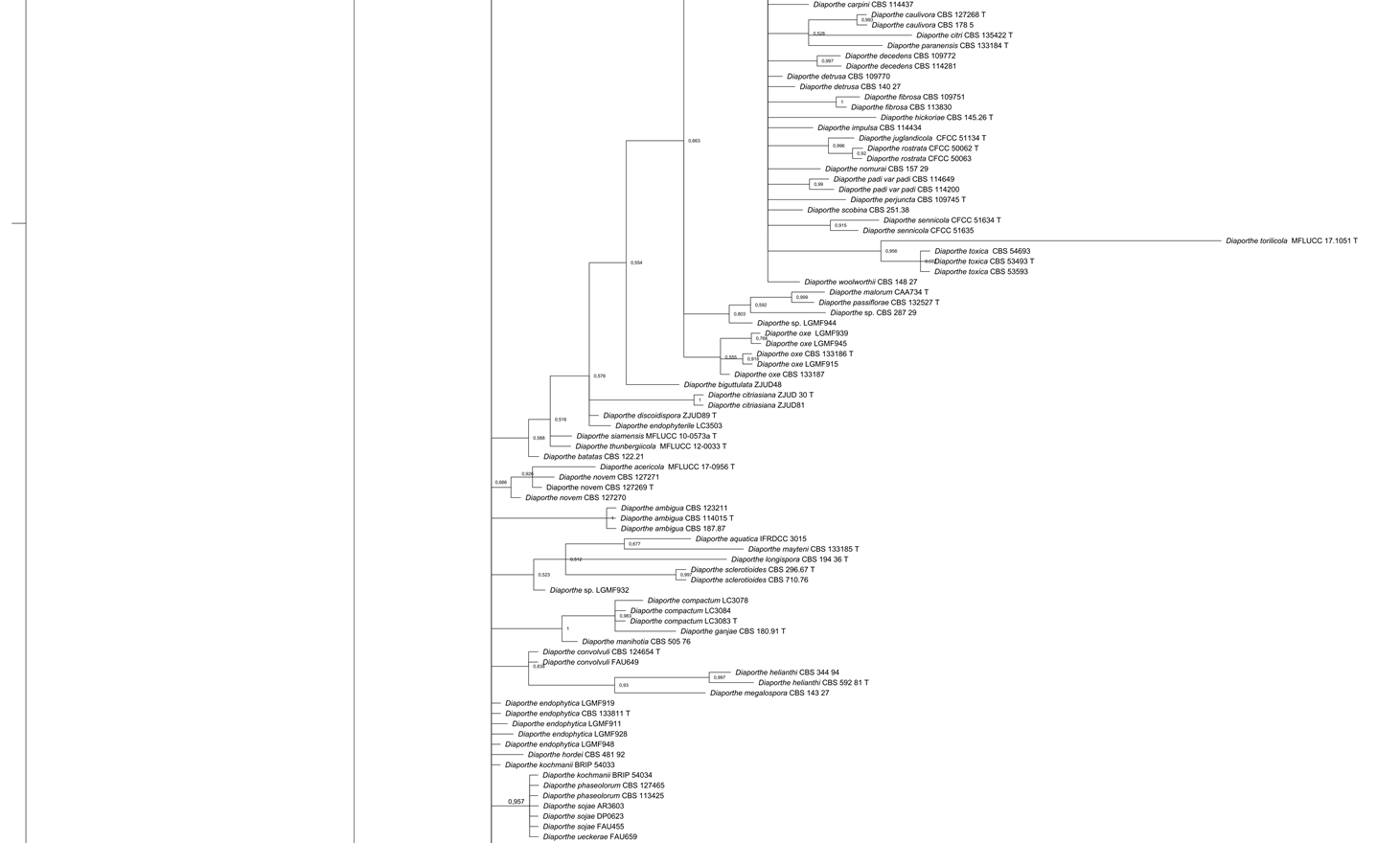


Figure S11-Bayesian phylogenetic tree based on ITS partial sequence of LGMF1610, LGMF1612, LGMF1614, 1538, LGMF1539, LGMF1540, LGMF1615, LGMF1616, LGMF1541, LGMF1618, LGMF1620, LGMF1548, 1549, LGMF1554, LGMF1629, LGMF 1561, LGMF1563, LGMF1631, LGMF1632, LGMF 1565, LGMF1567, LGMF 1569, LGMF 1633, LGMF1573, LGMF1576, LGMF1635, LGMF1583, LGMF1639, LGMF1593, LGMF1594, LGMF1601, LGMF1602, LGMF1508, LGMF1517, LGMF1606, LGMF1525, LGMF1526, LGMF1530, LGMF1531, LGMF1532, and LGMF1526 (bold) sequences of all accepted species from *Diaporthe* genus. The data matrix had 383 taxa and 537 characters. The tree was rooted to *Diaporthella* *corylina* (KC343004). T: type strain. (Continue)


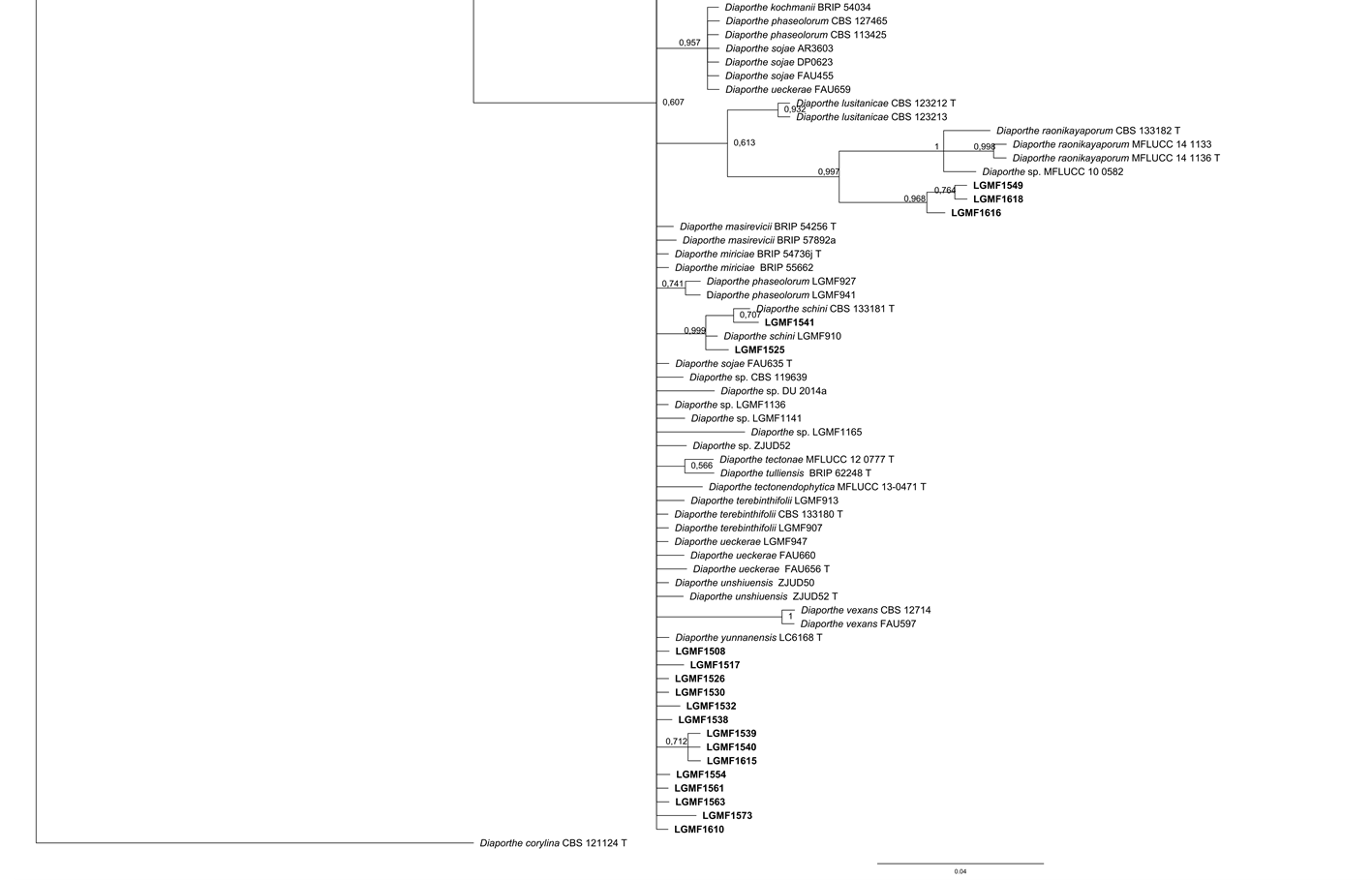


Figure S11-Bayesian phylogenetic tree based on ITS partial sequence of LGMF1610, LGMF1612, LGMF1614, 1538, LGMF1539, LGMF1540, LGMF1615, LGMF1616, LGMF1541, LGMF1618, LGMF1620, LGMF1548, 1549, LGMF1554, LGMF1629, LGMF 1561, LGMF1563, LGMF1631, LGMF1632, LGMF 1565, LGMF1567, LGMF 1569, LGMF 1633, LGMF1573, LGMF1576, LGMF1635, LGMF1583, LGMF1639, LGMF1593, LGMF1594, LGMF1601, LGMF1602, LGMF1508, LGMF1517, LGMF1606, LGMF1525, LGMF1526, LGMF1530, LGMF1531, LGMF1532, and LGMF1526 (bold) sequences of all accepted species from *Diaporthe* genus. The data matrix had 383 taxa and 537 characters. The tree was rooted to *Diaporthella* *corylina* (KC343004). T: type strain.

***Clade 2***


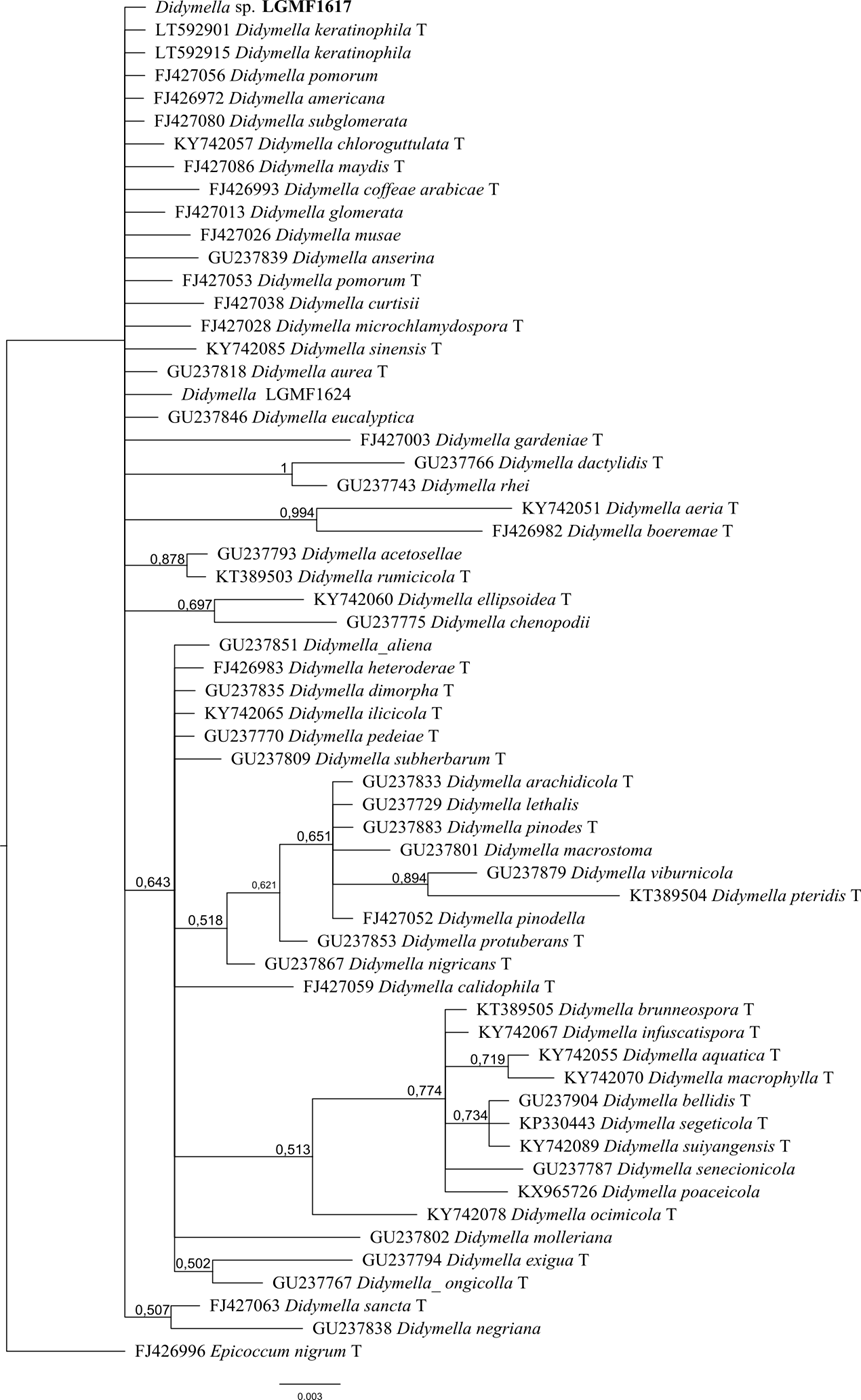


Figure S12-Bayesian phylogenetic tree based on ITS partial sequence of LGMF1617 (bold) identified as *Didymella* sp. and sequences of all accepted species from *Didymella*. The data matrix had 60 taxa and 497 characters. The tree was rooted to *Epicoccum* *nigrum* (FJ426996). Scale bar shows 0.003 changes and Bayesian posterior probability values are indicated at the nodes. T: type strain.


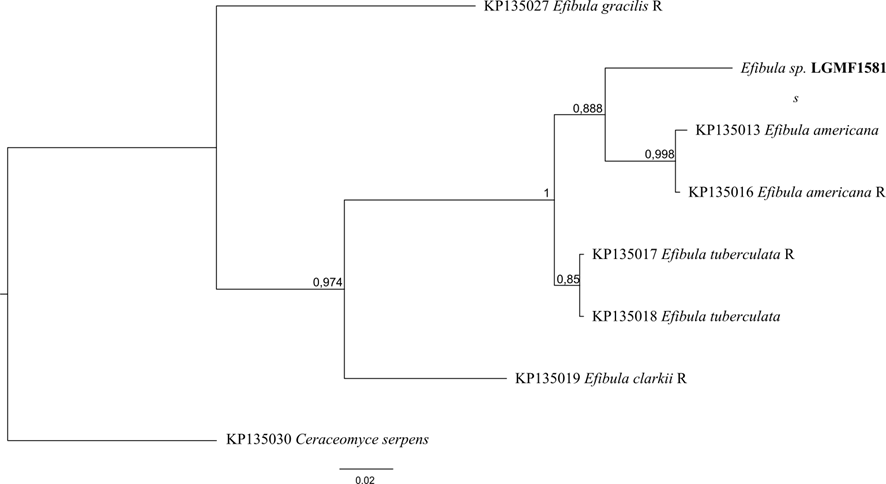


Figure S13-Bayesian phylogenetic tree based on ITS partial sequence of LGMF1581 (bold) identified as *Efibula* sp. and sequences of all accepted species from *Efibula* genus. The data matrix had 8 taxa and 615 characters. The tree was rooted to *Ceraceomyces* *serpens* (KP135030). Scale bar shows 0.02 changes and Bayesian posterior probability values are indicated at the nodes. T: type strain; R: representative strain.


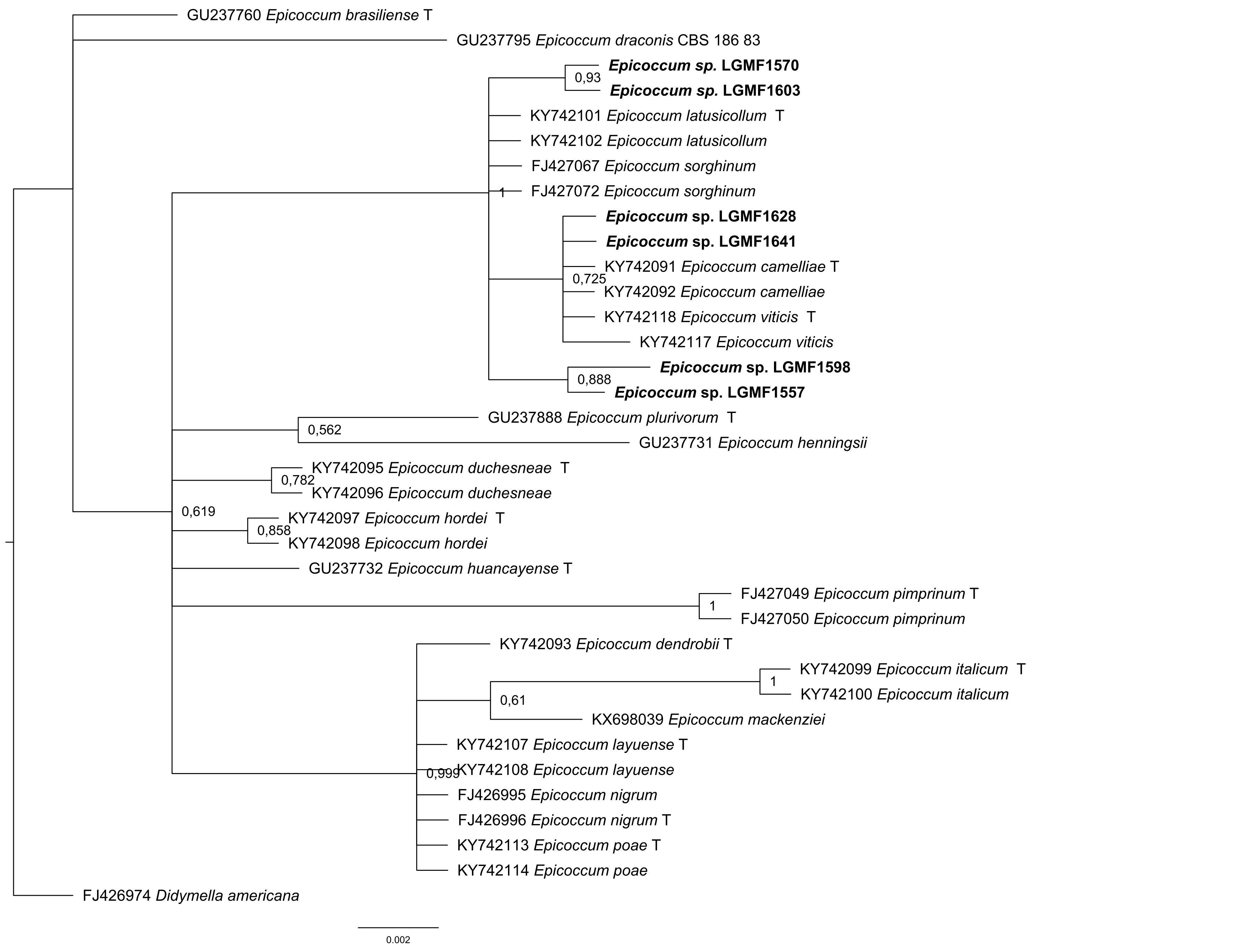


Figure S14-Bayesian phylogenetic tree based on ITS partial sequence of LGMF1603, LGMF1628, LGMF1641, LGMF1598 and LGMF1557 (bold) identified as *Epicoccum* sp. and sequences of all accepted species from *Epicoccum* genus The data matrix had 35 taxa and 455 characters. The tree was rooted to *Didymella americana* (FJ426974). Scale bar shows 0.002 changes and Bayesian posterior probability values are indicated at the nodes. T: type strain.


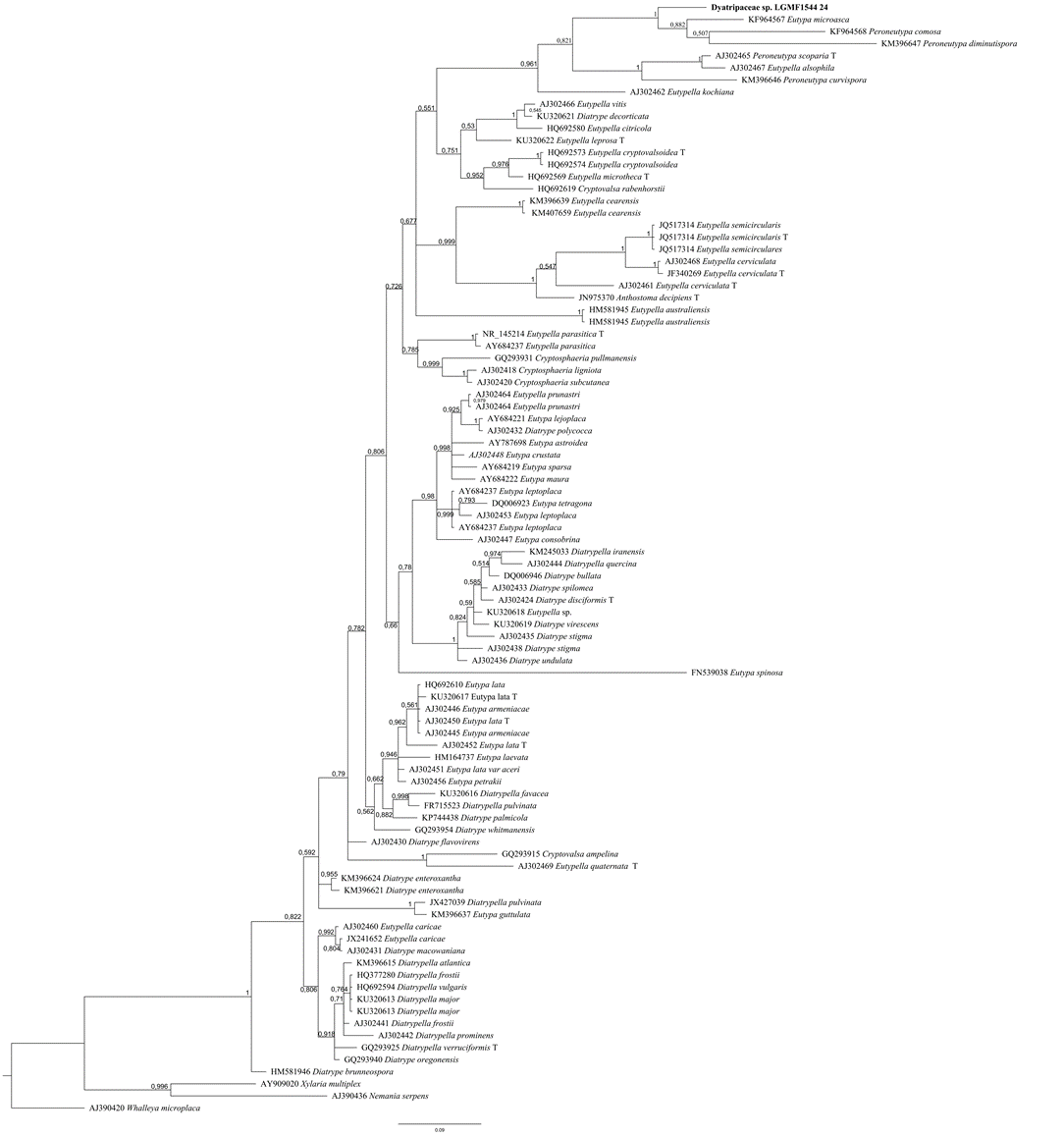


Figure S15-Bayesian phylogenetic tree based on ITS partial sequence of LGMF1544 (bold) identified as *Diatrypaceae* sp. and sequences of all accepted genera from *Diatrypaceae* family. The data matrix had 110 taxa and 641 characters. The tree was rooted to *Whalleya microplaca* (AJ4390420). Scale bar shows 0.002 changes and Bayesian posterior probability values are indicated at the nodes. T: type strain.


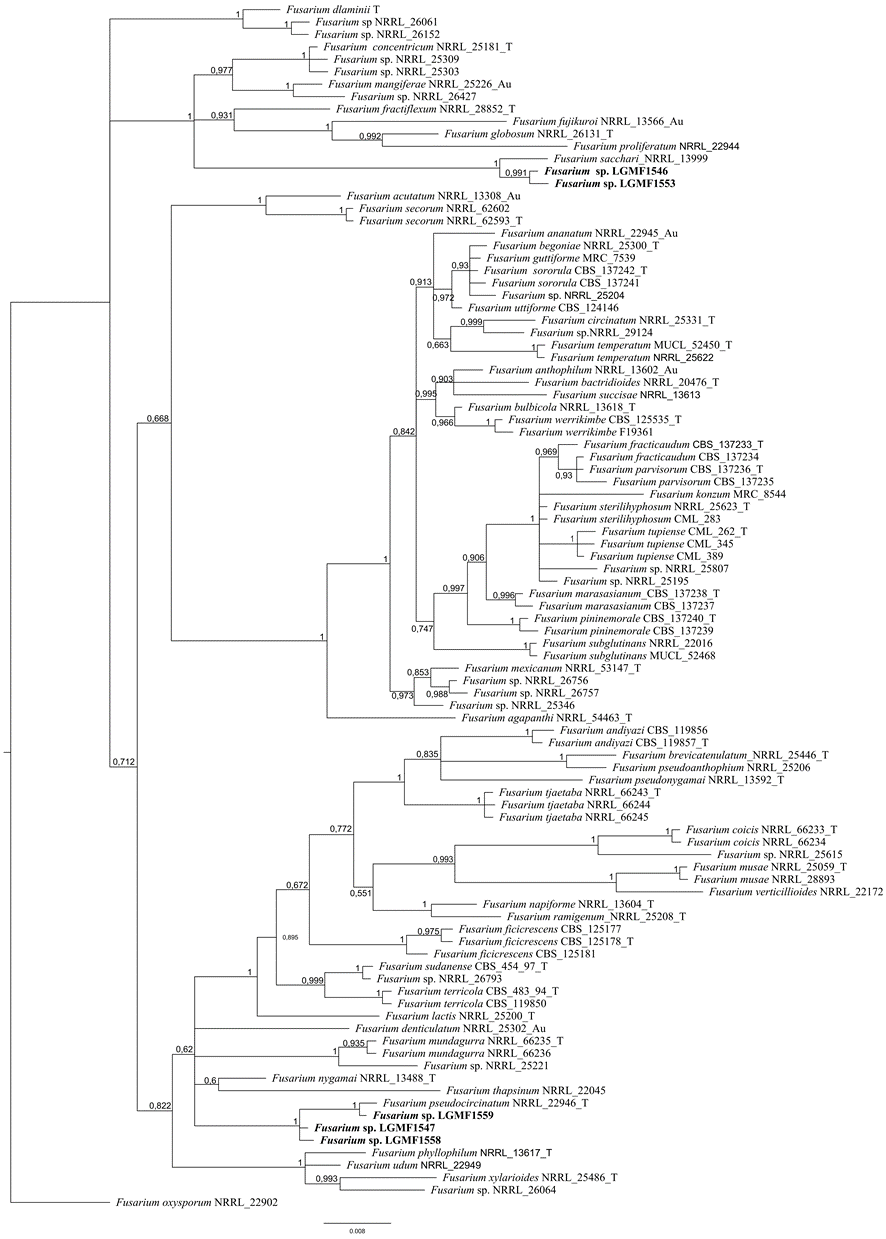


Figure S16-Bayesian phylogenetic tree based on EF partial sequence of LGMF1546, LGMF1553, LGMF1590, LGMF1547 and LGMF1558 (bold) identified as *Fusarium* sp. and sequences of all accepted species from *Fusarium* genus. The data matrix had 97 taxa and 680 characters. The tree was rooted to *Fusairum oxysporum* (NRRL22902). Scale bar shows 0.008 changes and Bayesian posterior probability values are indicated at the nodes. T: type strain.


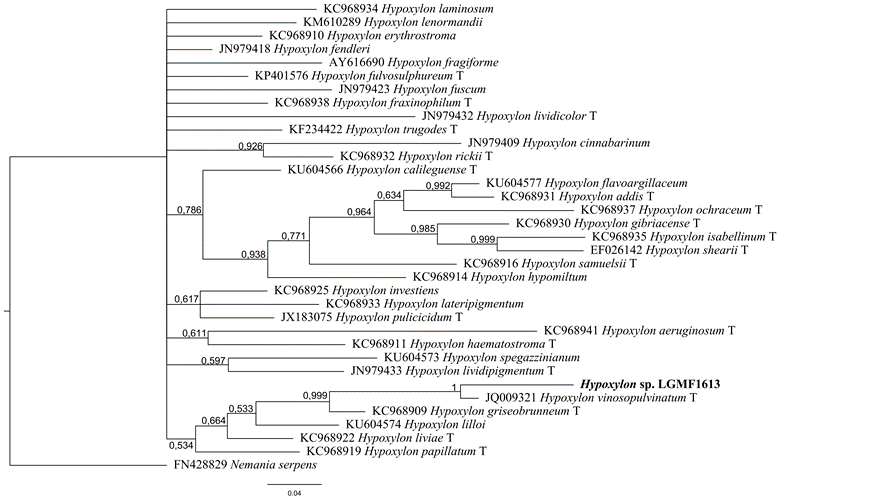


Figure S17-Bayesian phylogenetic tree based on ITS partial sequence of LGMF1613 (bold) identified as *Hypoxylon* sp. and sequences of all accepted species from *Hypoxylon* genus The data matrix had 35 taxa and 369 characters. The tree was rooted to *Nemania* *serpens* (FN428829). Scale bar shows 0.04 changes and Bayesian posterior probability values are indicated at the nodes T: type strain.


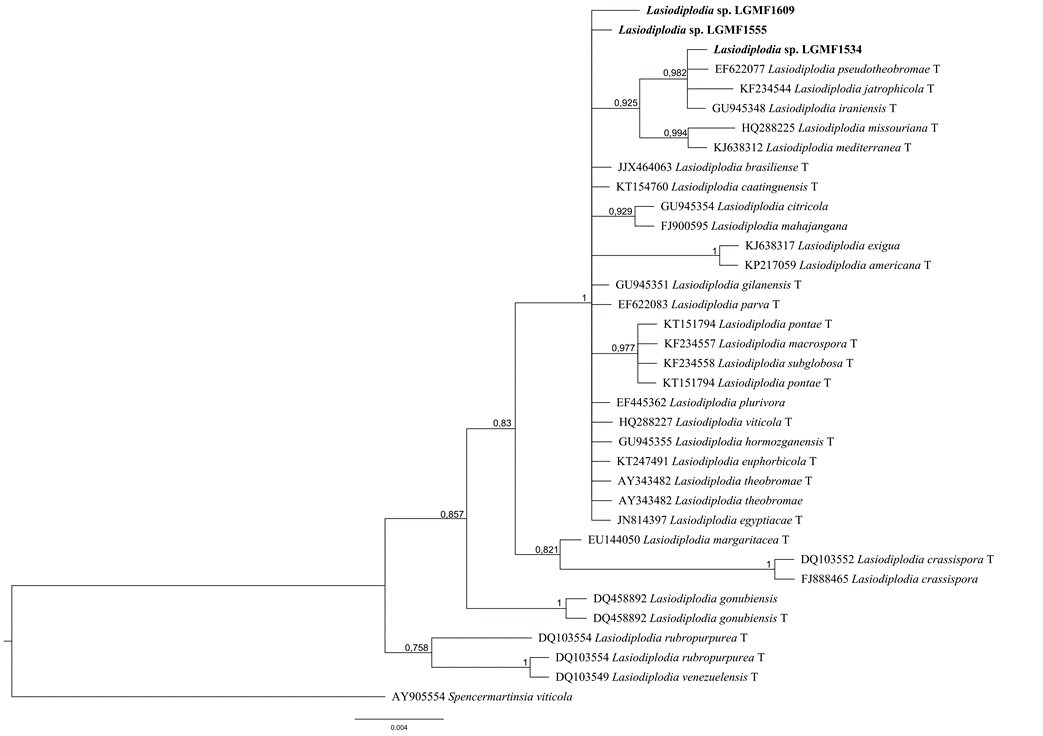


Figure S18-Bayesian phylogenetic tree based on ITS partial sequence of LGMF1609, LGMF1555 and LGMF1534 (bold) identified as of *Lasiodiplodia* sp. and sequences of all accepted species from *Lasiodiplodia* genus The data matrix had 34 taxa and 401 characters. The tree was rooted to *Spencermartinsia viticola* (AY905554). Scale bar shows 0.004 changes and Bayesian posterior probability values are indicated at the nodes T: type strain.


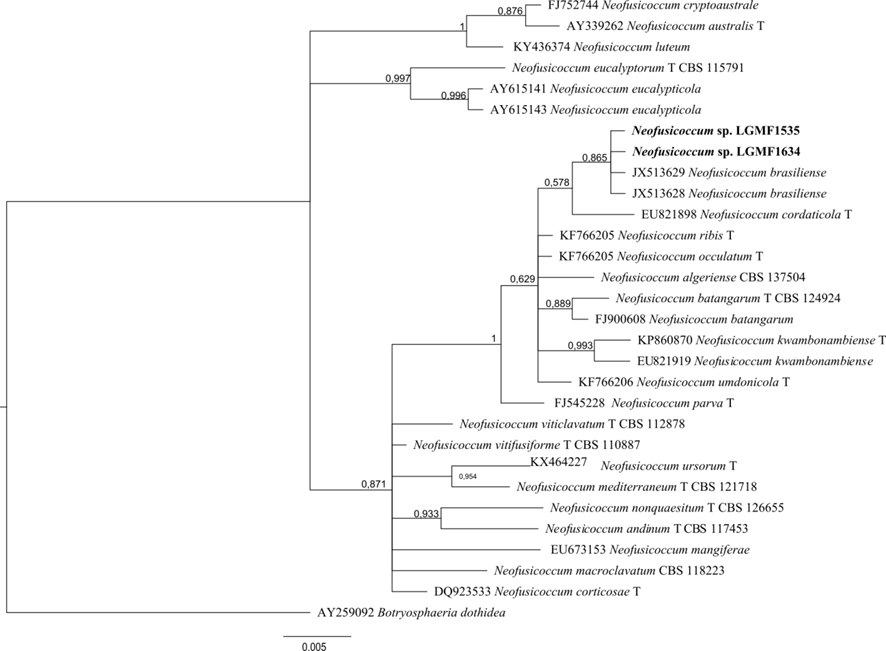


Figure S19-Bayesian phylogenetic tree based on ITS partial sequence of LGMF1535 and LGMF1634 (bold) identified as *Neofusicoccum brasiliense* and *Neofusicoccum* sp. The data matrix had 30 taxa and 505 characters. The tree was rooted to *Botryosphaeria dothidea* (AY259092). Scale bar shows 0.005 changes and Bayesian posterior probability values are indicated at the nodes. T: type strain.


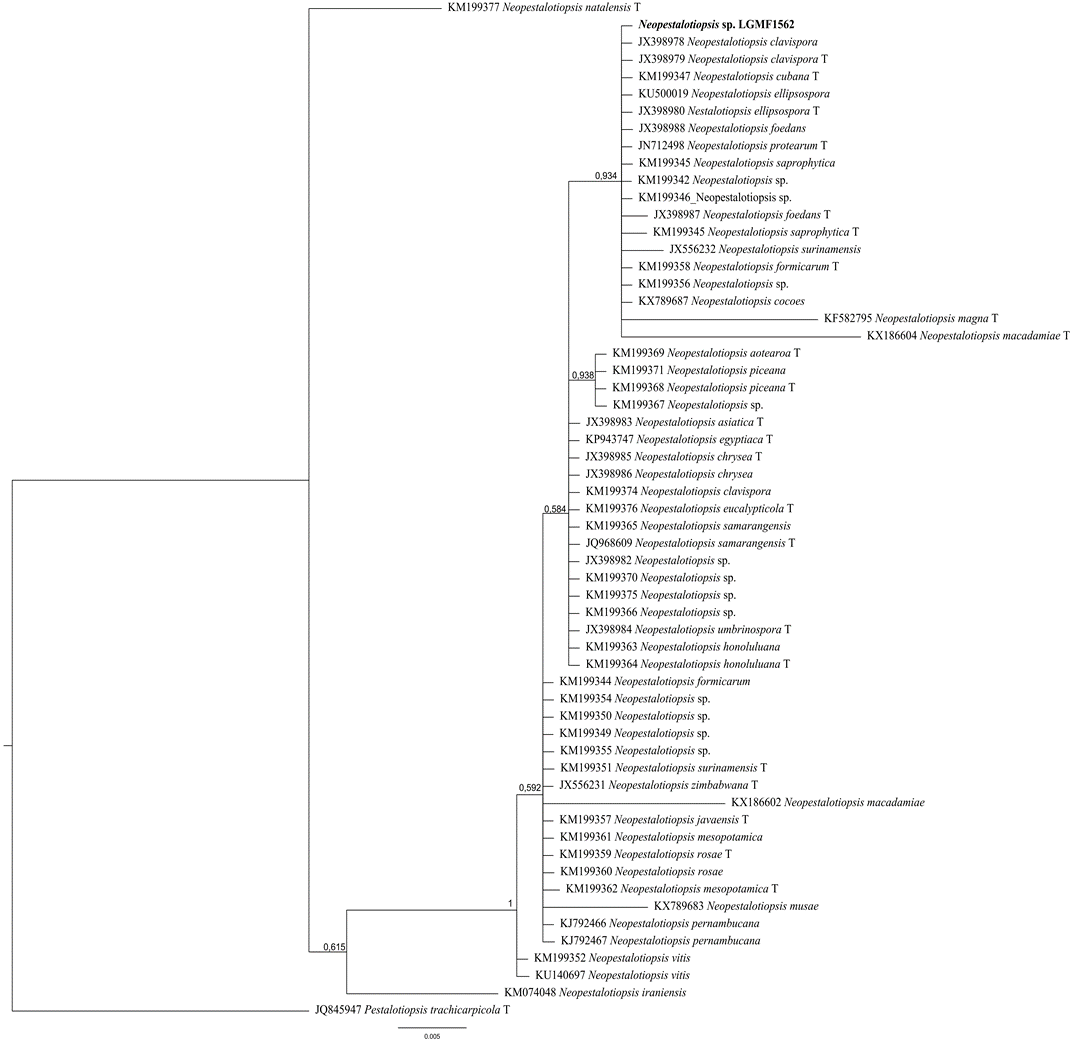
Figure S20-Bayesian phylogenetic tree based on ITS partial sequence of LGMF1562 (bold) identified as *Neopestalotiopsis* sp. and sequences of all accepted species from *Neopestalotiopsis* genus. The data matrix had 59 taxa and 480 characters. The tree was rooted to *Pestalotiopsis trachicarpicola* (JQ845947). T: type strain.


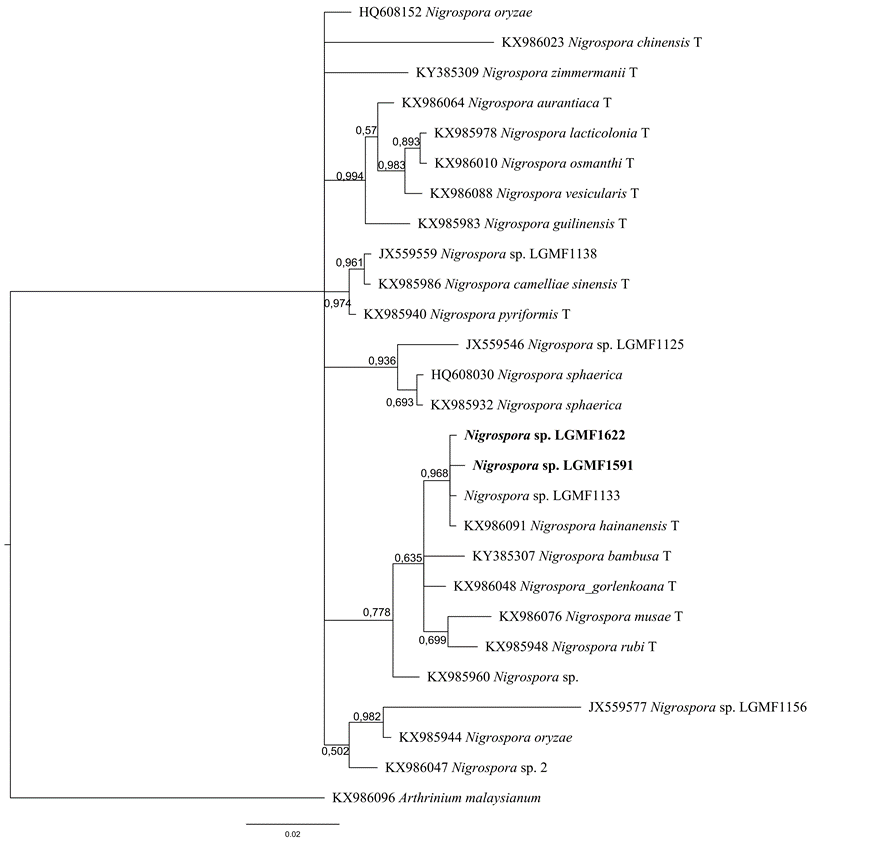
 Figure S21-Bayesian phylogenetic tree based on ITS partial sequence of LGMF1622 and LGMF1591 (bold) identified as *Nigrospora hainanensis* and sequences of all accepted species from *Nigrospora* genus. The data matrix had 27 taxa and 500 characters. The tree was rooted to *Arthrinium malaysianum* (KX986096)*.* Scale bar shows 0.02 changes and Bayesian posterior probability values are indicated at the nodes. T: type strain.


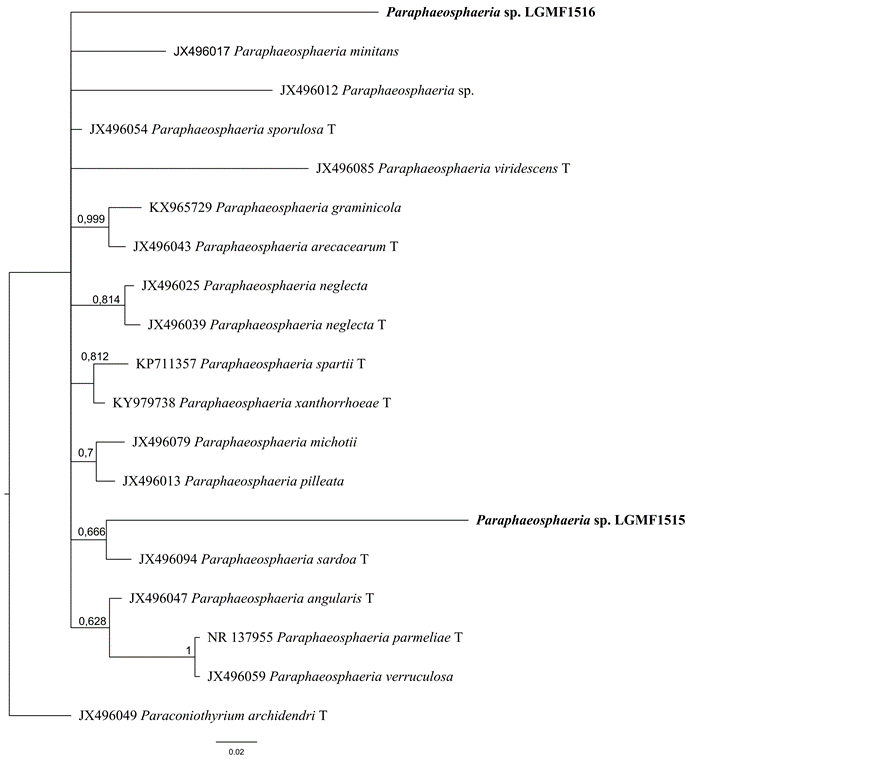


Figure S22-Bayesian phylogenetic tree based on ITS partial sequence of LGMF1515 and LGMF1516 (bold) identified as *Paraphaeophaeria* sp. and sequences of all accepted species from *Paraphaeophaeria* genus. The data matrix had 19 taxa and 488 characters. The tree was rooted to *Paraconiothyrium archidendri* (JX496049). Scale bar shows 0.02 changes and Bayesian posterior probability values are indicated at the nodes. T: type strain.


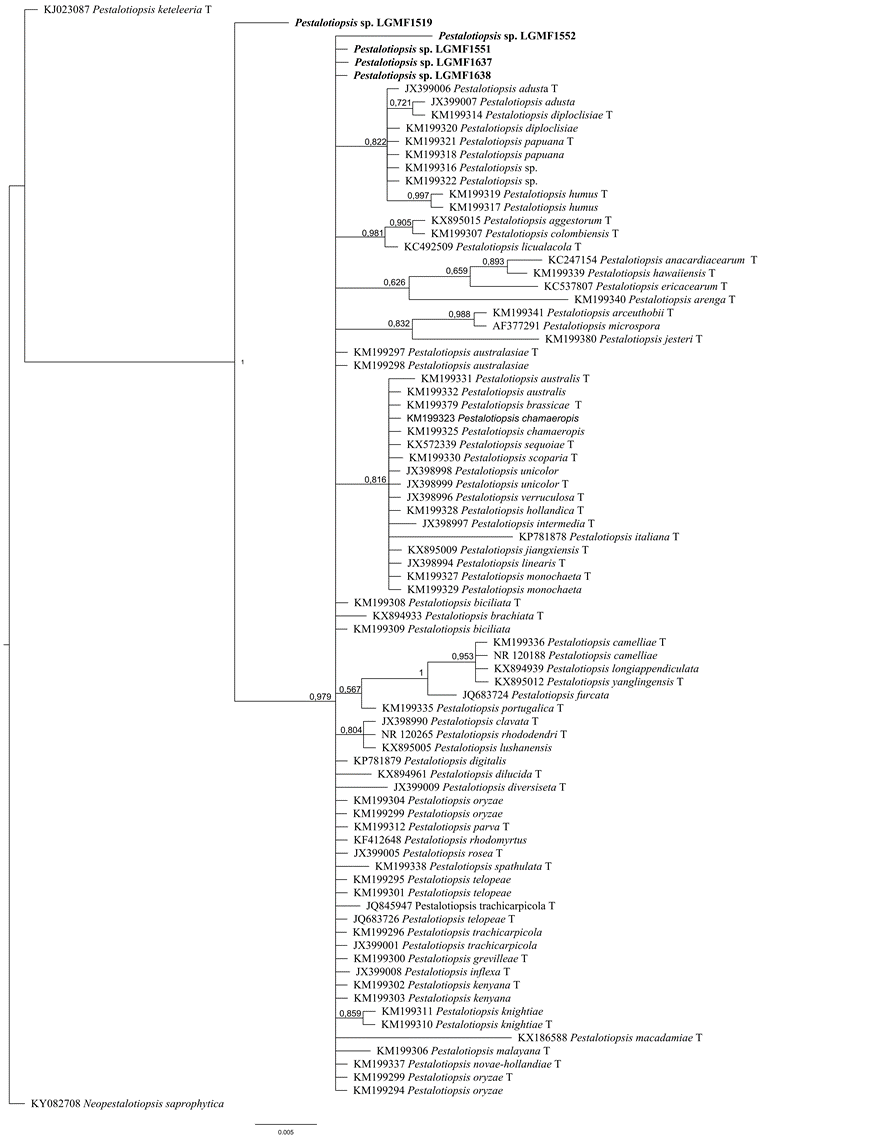


Figure S23-Bayesian phylogenetic tree based on ITS partial sequence of LGMF1519, LGMF1552, LGMF1551, LGMF1637 and LGMF1638 (bold) identified as *Pestalotiopsis* sp. and sequences of all accepted species from *Pestalotiopsis* genus. The data matrix had 84 taxa and 376 characters. The tree was rooted to *Neopestalotiopsis* *saprophytica* (KY082708). Scale bar shows 0.02 changes and Bayesian posterior probability values are indicated at the nodes. T: type strain.


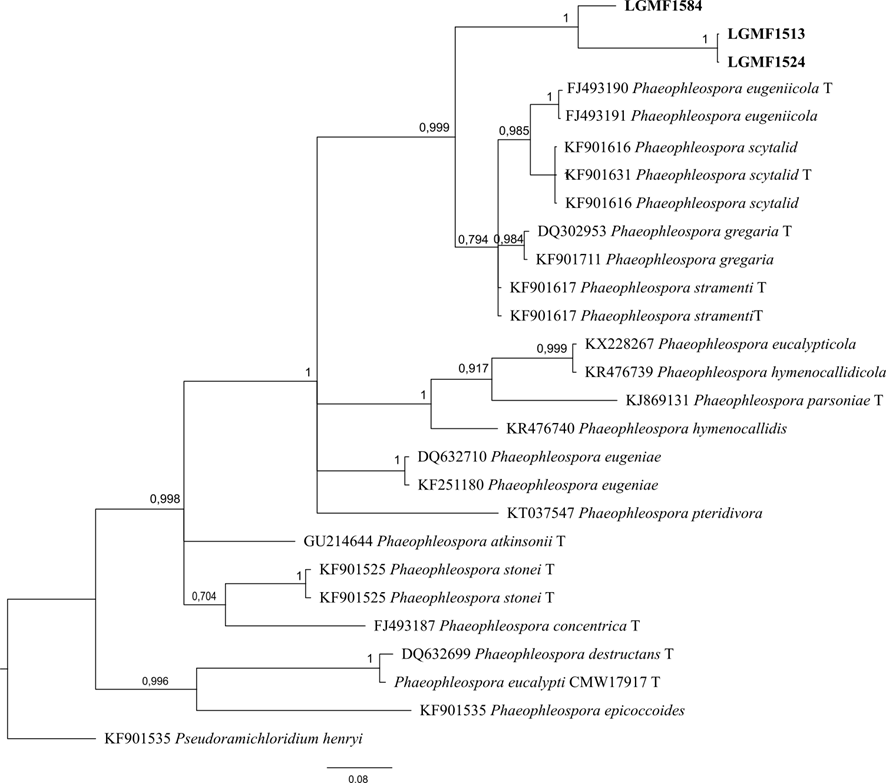


Figure S24-Bayesian phylogenetic tree based on ITS partial sequence of LGMF1584, LGMF1513 and LGMF1524 (bold) identified as *Phaeophleospora* sp. and sequences of all accepted species from *Phaeophleospora* genus. The data matrix had 27 taxa and 496 characters. The tree was rooted to *Pseudoramichloridium henryi* (KF901535). Scale bar shows 0.08 changes and Bayesian posterior probability values are indicated at the nodes. T: type strain


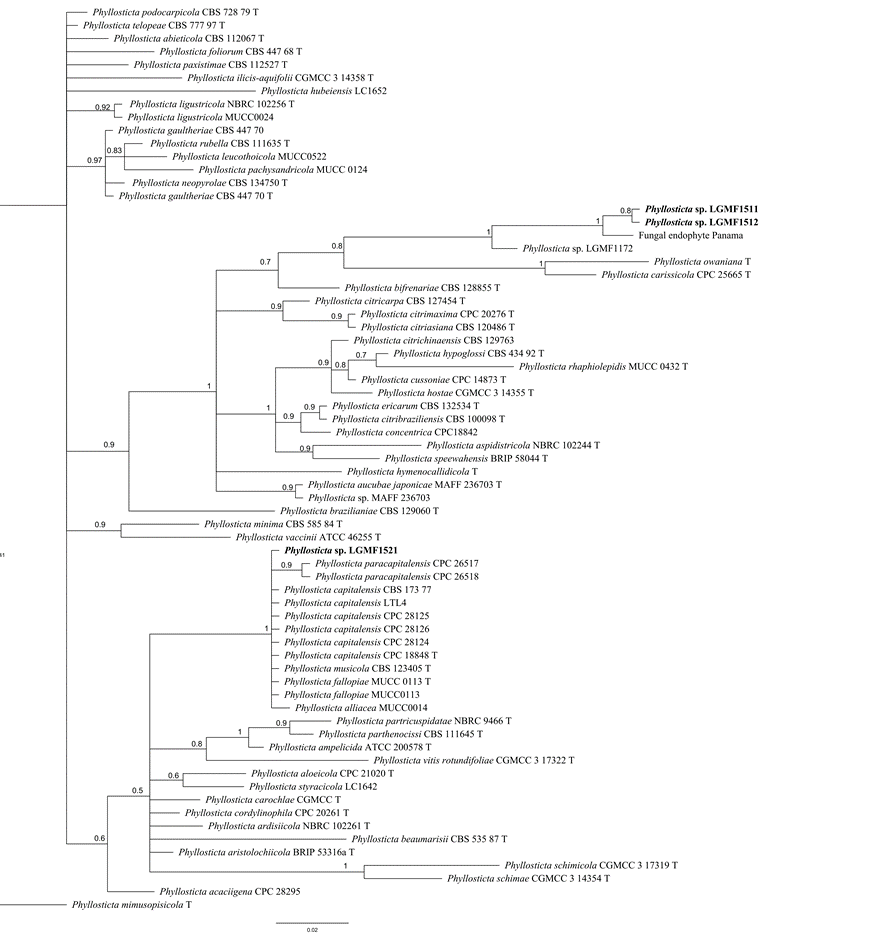


Figure S25-Bayesian phylogenetic tree based on ITS partial sequence of LGMF1584, LGMF1513 and LGMF1524 (bold) identified as *Phyllosticta* sp. and sequences of all accepted species from *Phyllosticta* genus. The data matrix had 69 taxa and 358 characters. The tree was rooted to *Phyllosticta mimusopisicola.* Scale bar shows 0.02 changes and Bayesian posterior probability values are indicated at the nodes. T: type strain.


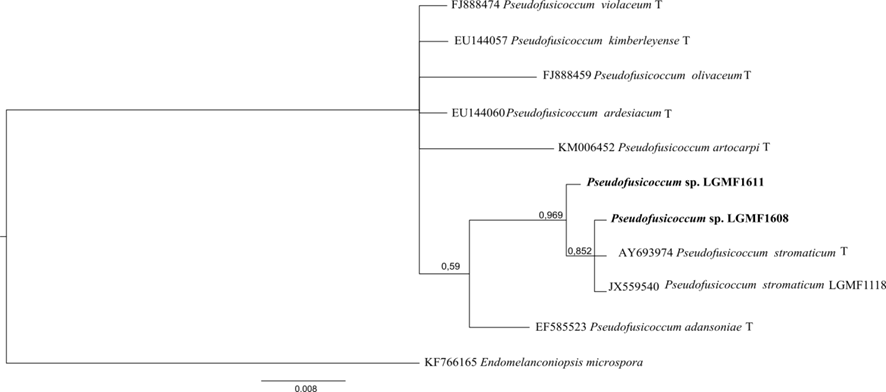


Figure S26-Bayesian phylogenetic tree based on ITS partial sequence of LGMF1611 and LGMF1608 (bold) identified as *Pseudofusicoccum stromaticcum* and *Pseudofusicoccum* sp. and sequences of all accepted species from *Pseudofusicoccum* genus. The data matrix had 11 taxa and 519 characters. The tree was rooted to *Endomelanconiopsis microspore* (KF766165). Scale bar shows 0.008 changes and Bayesian posterior probability values are indicated at the nodes. T: type strain.


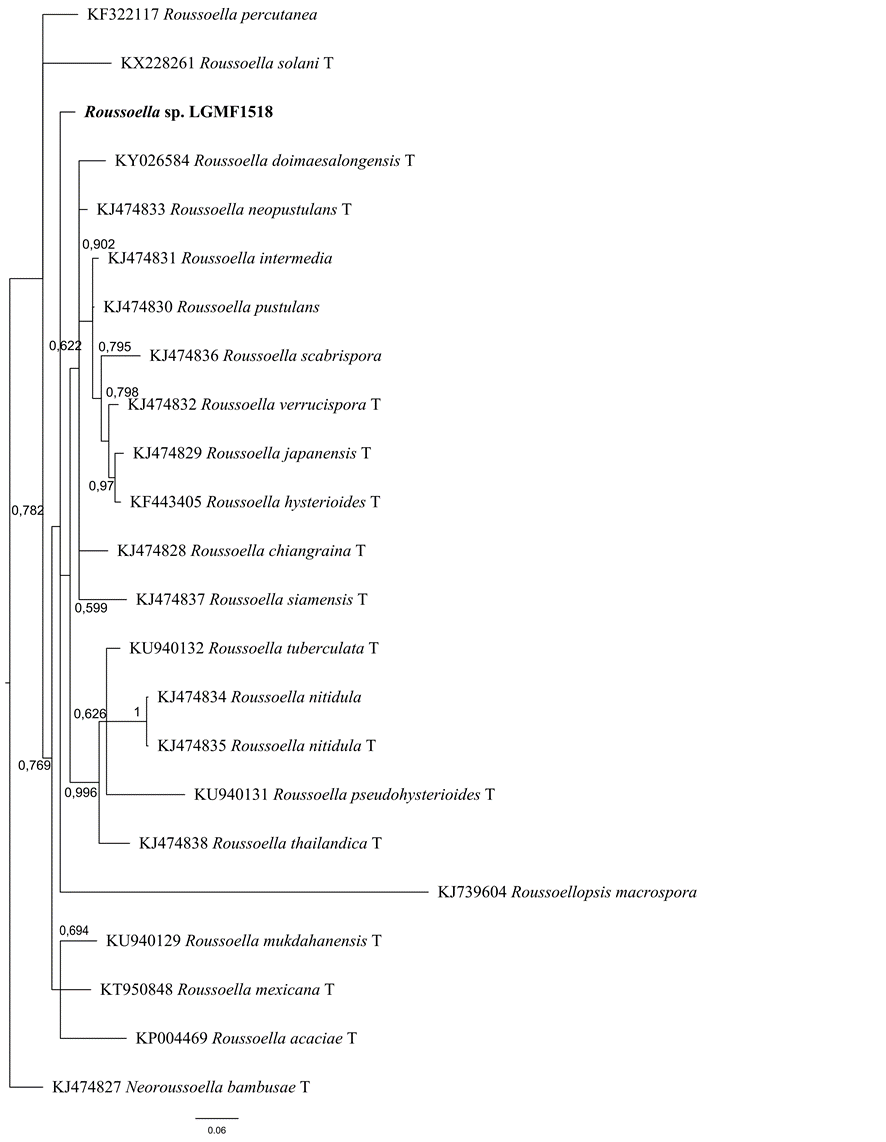


Figure S27-Bayesian phylogenetic tree based on ITS partial sequence of LGMF1518 (bold) identified as *Roussoella* sp. and sequences of all accepted species from *Roussoella* genus. The data matrix had 23 taxa and 388 characters. The tree was rooted to *Neoroussoella bambusae* (KJ474827). Scale bar shows 0.06 changes and Bayesian posterior probability values are indicated at the nodes. T: type strain.


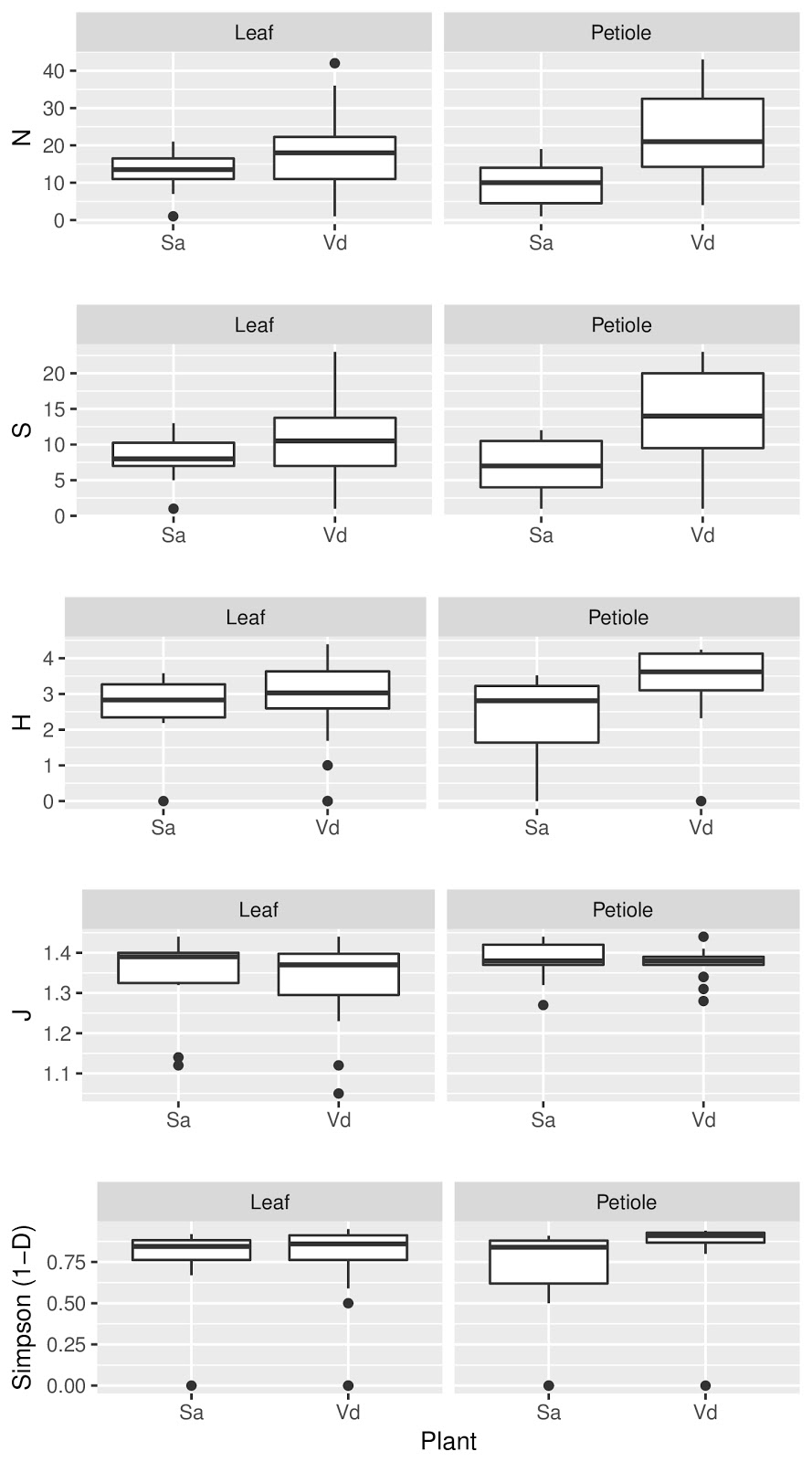

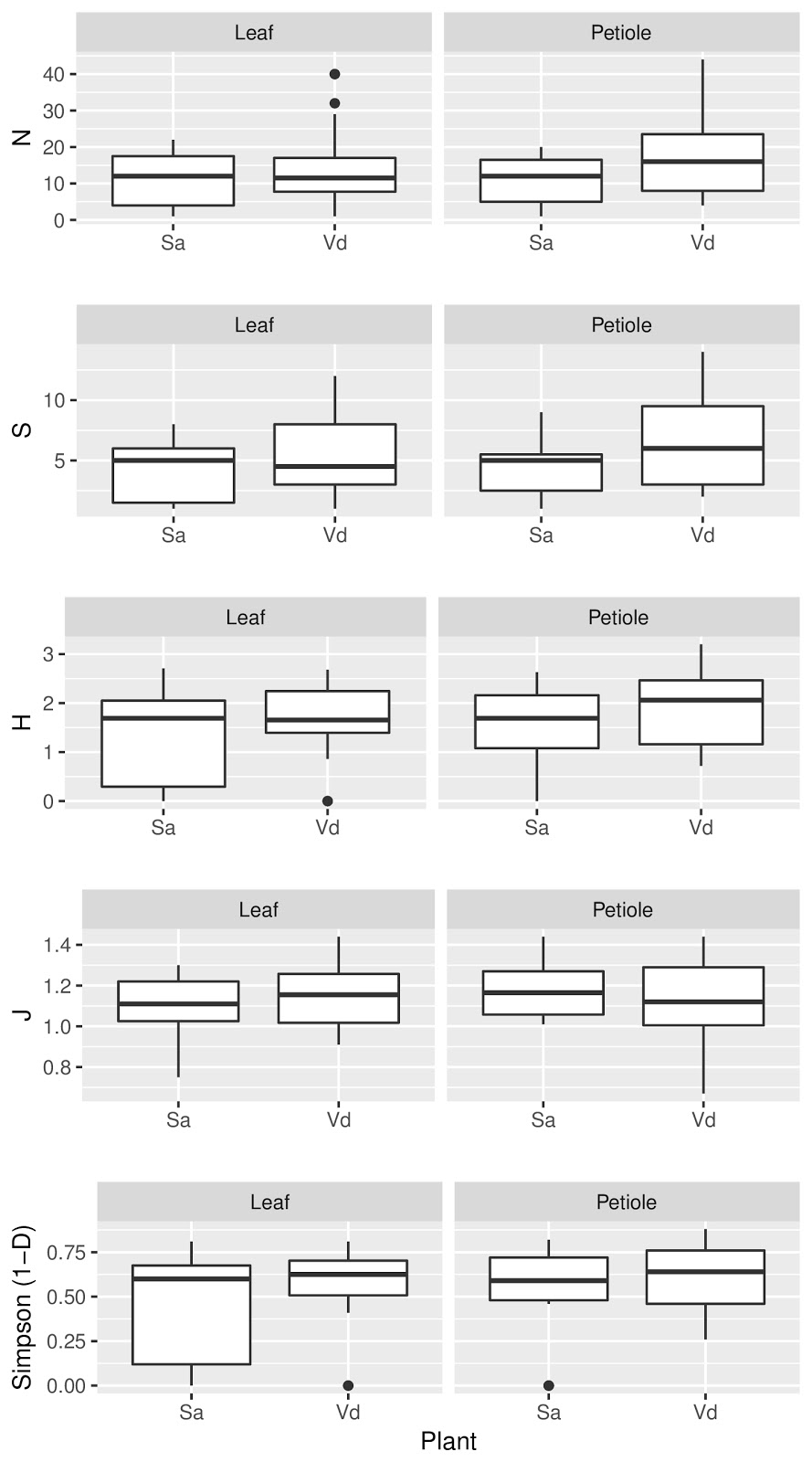


**Figure S28-**Boxplot graphic showing median, quartiles and outliers for diversity indexes, abundance and richness A) in Morphotype and B) in Genera. Note: Vd for *Vochysia divergens* and Sa for *Stryphnodendron adstringens.*


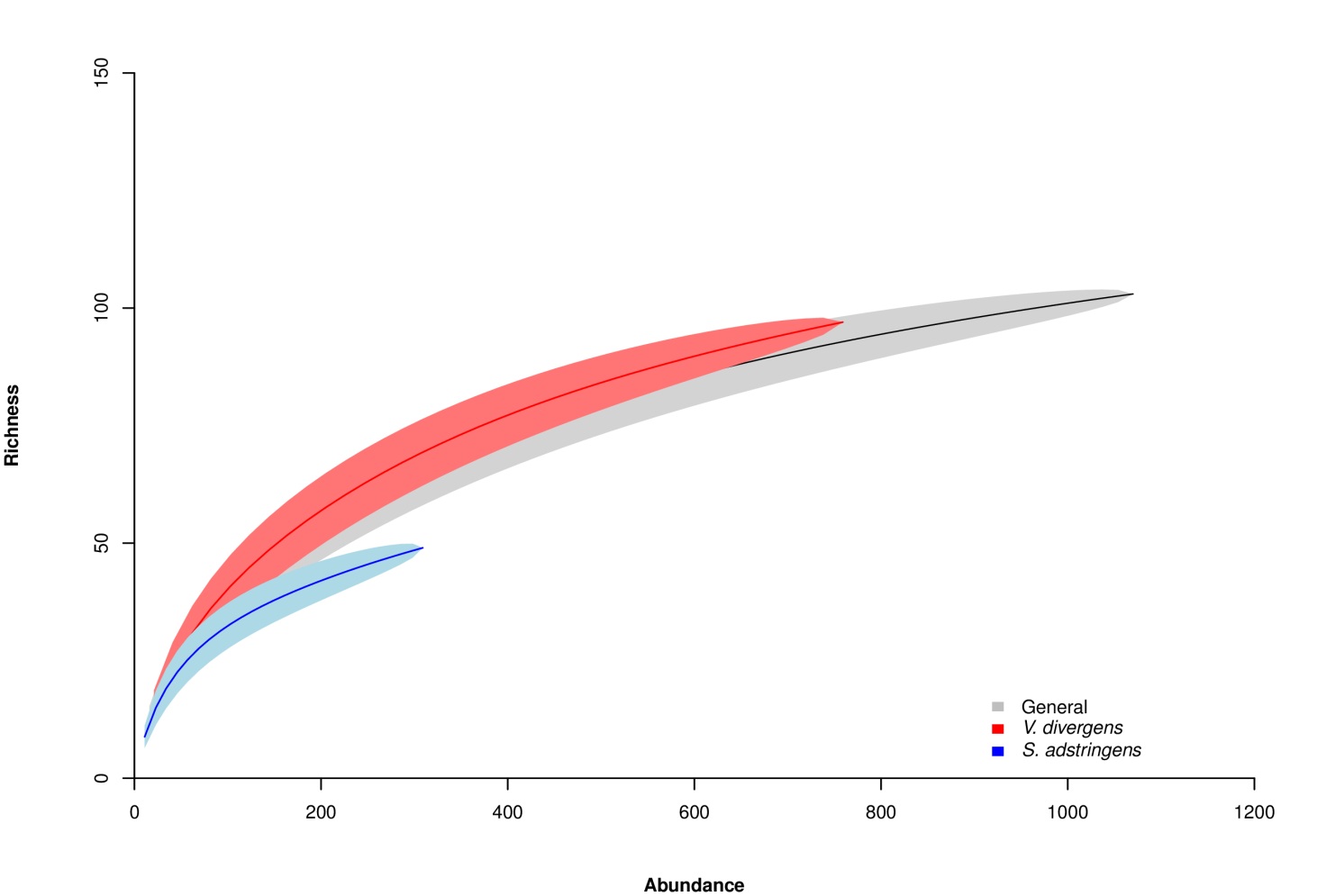


**Figure S29-**Rarefaction curve of richness and abundance per morphotype of endophytic fungi associated with *Vochysia divergens* (blue), *Stryphnodendron adstringens* (green) and both (grey).


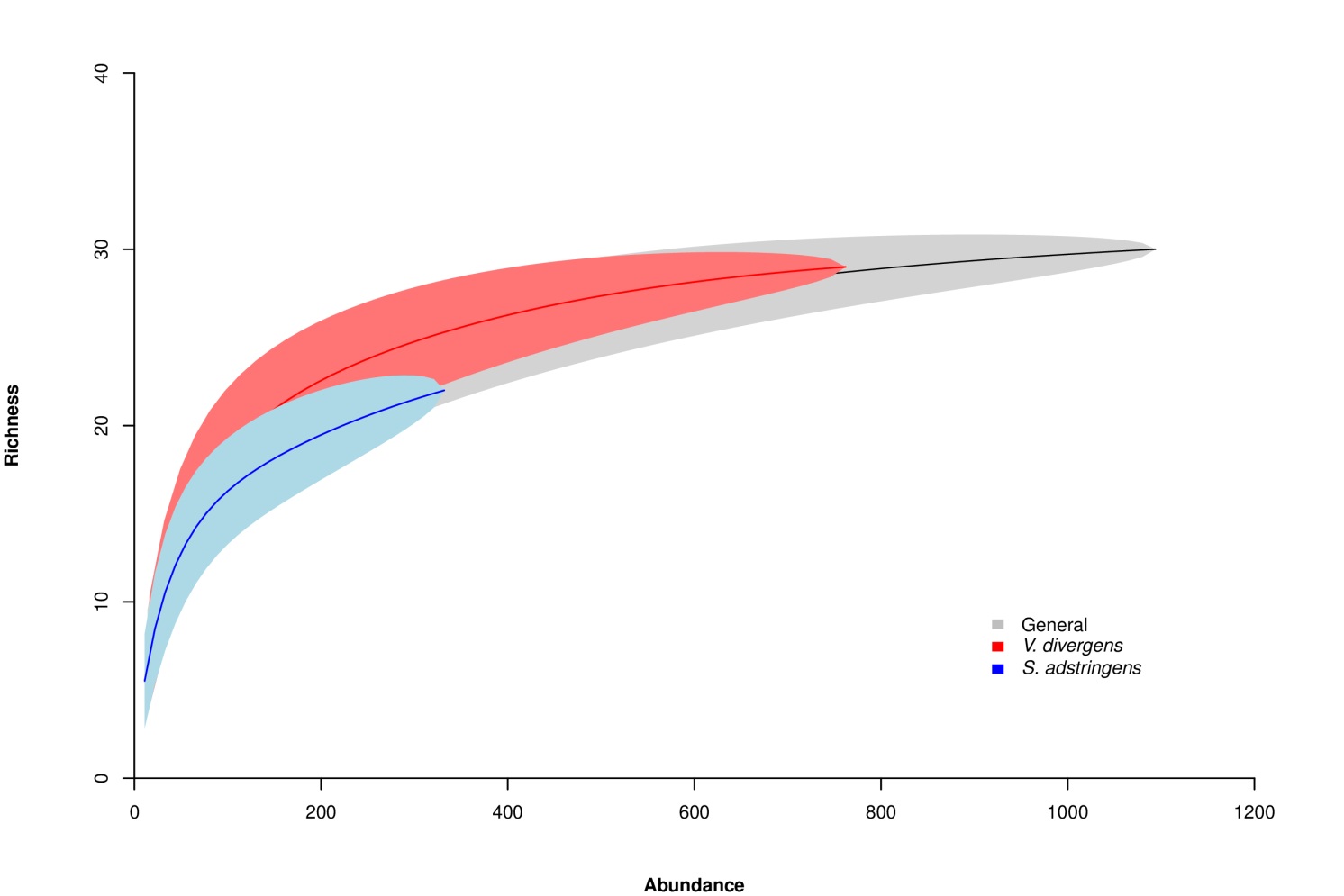


**Figure S30-**Rarefaction curve of richness and abundance per genus of endophytic fungi associated with *Vochysia divergens* (blue), *Stryphnodendron adstringens* (green) and both (grey).

.
